# Copper(II) Complexes with Isomeric Morpholine-Substituted 2-Formylpyridine Thiosemicarbazone Hybrids as Potential Anticancer Drugs Inhibiting Both Ribonucleotide Reductase and Tubulin Polymerization: The Morpholine Position Matters

**DOI:** 10.1021/acs.jmedchem.4c00259

**Published:** 2024-05-21

**Authors:** Miljan N. M. Milunovic, Katerina Ohui, Iuliana Besleaga, Tatsiana V. Petrasheuskaya, Orsolya Dömötör, Éva A. Enyedy, Denisa Darvasiova, Peter Rapta, Zuzana Barbieriková, Daniel Vegh, Szilárd Tóth, Judit Tóth, Nóra Kucsma, Gergely Szakács, Ana Popović-Bijelić, Ayesha Zafar, Jóhannes Reynisson, Anatoly D. Shutalev, Ruoli Bai, Ernest Hamel, Vladimir B. Arion

**Affiliations:** † Institute of Inorganic Chemistry, University of Vienna, Vienna A-1090, Austria; ‡ Department of Molecular and Analytical Chemistry, Interdisciplinary Excellence Centre, University of Szeged, Dóm tér 7-8, Szeged H-6720, Hungary; § MTA-SZTE Lendület Functional Metal Complexes Research Group, University of Szeged, Dóm tér 7, Szeged H-6720, Hungary; ∥ Institute of Physical Chemistry and Chemical Physics, Faculty of Chemical and Food Technology, Slovak University of Technology in Bratislava, Bratislava SK-81237, Slovakia; ⊥ Institute of Organic Chemistry, Faculty of Chemical and Food Technology, Slovak University of Technology in Bratislava, Bratislava SK-81237, Slovakia; # Institute of Molecular Life Sciences, HUN-REN Research Centre for Natural Sciences, Hungarian Research Network, Magyar Tudósok körútja 2, Budapest H-1117, Hungary; ∇ Center for Cancer Research, Medical University of Vienna, Vienna A-1090, Austria; ○ Faculty of Physical Chemistry, University of Belgrade, Belgrade 11158, Serbia; ◆ School of Chemical Sciences, University of Auckland, Private Bag 92019, Auckland 1142, New Zealand; ¶ School of Pharmacy and Bioengineering, Keele University, Newcastle-under-Lyme, Staffordshire ST5 5BG, United Kingdom; & N. D. Zelinsky Institute of Organic Chemistry, Russian Academy of Sciences, Moscow 119991, Russian Federation; ● Molecular Pharmacology Branch, Developmental Therapeutics Program, Division of Cancer Diagnosis and Treatment, National Cancer Institute, Frederick National Laboratory for Cancer Research, National Institutes of Health, Frederick, Maryland 21702, United States; ◊ Inorganic Polymers Department, “Petru Poni” Institute of Macromolecular Chemistry, Aleea Gr. Ghica Voda 41 A, Iasi 700487, Romania

## Abstract

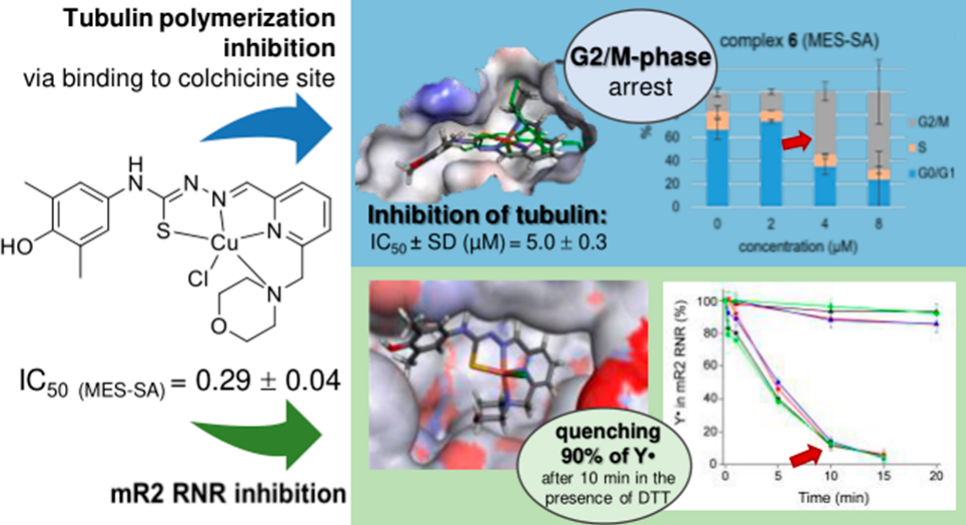

The development of copper(II) thiosemicarbazone complexes as potential anticancer agents, possessing dual functionality as inhibitors of R2 ribonucleotide reductase (RNR) and tubulin polymerization by binding at the colchicine site, presents a promising avenue for enhancing therapeutic effectiveness. Herein, we describe the syntheses and physicochemical characterization of four isomeric proligands **H**_**2**_**L**^**3**^–**H**_**2**_**L**^**6**^, with the methylmorpholine substituent at pertinent positions of the pyridine ring, along with their corresponding Cu(II) complexes **3**–**6**. Evidently, the position of the morpholine moiety and the copper(II) complex formation have marked effects on the *in vitro* antiproliferative activity in human uterine sarcoma MES-SA cells and the multidrug-resistant derivative MES-SA/Dx5 cells. Activity correlated strongly with quenching of the tyrosyl radical (Y^•^) of mouse R2 RNR protein, inhibition of RNR activity in the cancer cells, and inhibition of tubulin polymerization. Insights into the mechanism of antiproliferative activity, supported by experimental results and molecular modeling calculations, are presented.

## 1 Introduction

Effective treatment of metastatic cancers usually requires the use of chemotherapy, but anticancer therapy has low selectivity and serious side effects.^1−10^ Moreover, multiple drugs are used, due to frequent resistance to single agents, and the overexpression of multidrug resistance (MDR) transporters such as P-glycoprotein (Pgp), which efflux drugs from the cells, thus keeping their concentrations below a cell-killing threshold.^11,12^ The search for more efficient, safer anticancer agents unaffected by MDR is warranted.

Malignant cells rapidly divide, relying extensively on the synthesis of DNA building blocks through the *de novo* production of deoxyribonucleotide triphosphates (dNTPs). The pivotal role in this process belongs to the oxygen-dependent, iron-containing enzyme ribonucleotide reductase (RNR), making it an important biomolecular target in anticancer treatment. RNRs catalyze the reduction of nucleoside 5′-diphosphates (NDPs) to their deoxy derivatives (dNDPs). The active form of the enzyme consists of the two subunits α (R1) and β (R2), forming a tetrameric quaternary structure α_2_β_2_ in *Escherichia coli* or more complex α_*n*_β_*m*_ (*n* = 2, 4, 6 and *m* = 1–3) structures in eukaryotes.^13,14^ RNR activity requires the presence of the sufficiently stable diferric-tyrosyl radical cofactor [(Fe^III^–O–Fe^III^)–Y^•^], located in the R2 subunit. The cofactor is assembled *in vivo* from apo-R2 and Fe^II^ (from intracellular pools) in the presence of O_2_. Once produced, the tyrosyl radical (Y^•^) is able to carry out many turnovers of NDPs to dNDPs.^13^ Besides its classical role in DNA replication and repair, RNRs have diverse functions in other biological processes, including mitochondrial DNA replication, cell cycle regulation, and apoptosis.^15^

Tubulin, the repeating subunit of microtubules (MTs), is another validated molecular target for antitumor drugs.^16^ MTs play crucial roles in cell division,^17^ cell shape, and cell motility^18−20^ and in other vital cellular processes, such as cell signaling and intracellular transport.^19,21,22^ Interference with MT dynamics by MT-targeted agents (MTAs) during mitosis affects chromosome separation, disrupting cell cycle progression, and this results in cell death.^23,24^

Thiosemicarbazones (TSCs) with α-*N*-heterocyclic backbone are excellent iron chelators and can be potent R2 RNR inhibitors.^25^ They are 100–2000-fold more potent than hydroxyurea, 3,4-dihydroxybenzohydroxamic acid (Didox), 3,4,5-trihydroxybenzamidoxime (Trimidox), pyrogallol, and *p*-alkoxyphenols acting as Y^•^ scavengers, and they also show marked anticancer activity.^26^

TSCs also have remarkable tubulin-inhibitory activity.^27^ Recently, we reported that copper(II) complexes with indolobenzazocine-based Schiff bases, which share with TSCs a similar tridentate binding motif, show significant antiproliferative activity and act as colchicine site inhibitors.^28^ The combination of MTAs with anticancer drugs with other mechanisms of action is widely recognized as one of the best strategies to mitigate MDR. Tubulin inhibitors targeting the colchicine site are candidates for better clinical outcomes,^29,30^ being less vulnerable to Pgp overexpression than other antitubulin agents^31,32^ and being active against cancer cells overexpressing β3-tubulin.^29^

**Chart 1:**
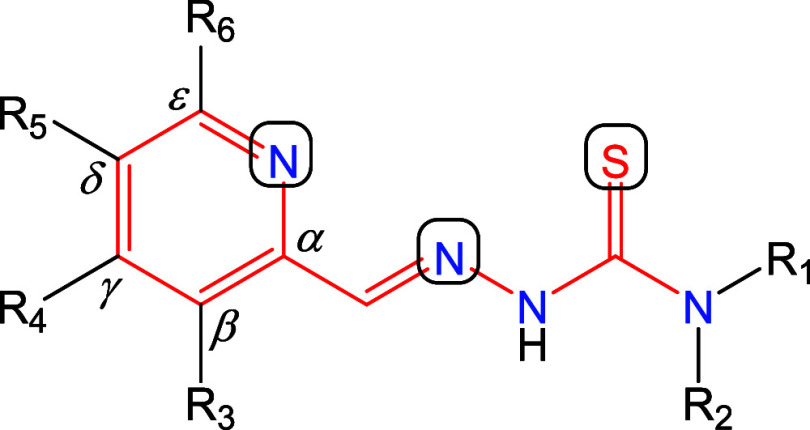
Line drawings of TSCs with an α-*N*-heterocyclic backbone (red), NNS binding site, R_1_, R_2_ substituents at the terminal N atom, R_3_–R_6_ substituents at the pyridine ring labeled with the Greek letters (β–ε)

The versatility of the α-*N*-heterocyclic TSC scaffold allows for it to be designed with better pharmacological profiles, i.e., enhanced cytotoxic potency, equal or elevated toxicity against MDR cells,^33−35^ tuned solubility, and improved bioavailability of the prodrug, by (i) structural modification at the *N*-terminal atom of the thiosemicarbazide moiety (R_1_, R_2_) and/or (ii) introduction of polar functional groups R_3_–R_6_ at the pyridine ring in the β–ε positions (Chart 1).

Current efforts to further optimize the pharmacological profile of 2-formylpyridine TSCs and their copper(II) complexes is well documented in the literature.^36−44^ Recently, we reported that TSC proligands with a morpholine moiety attached at the δ-position of the pyridine ring and their copper(II) complexes display stronger antiproliferative activity than Triapine, even though their mouse R2 (mR2) RNR inhibitory activity in the presence of dithiothreitol (DTT) was only moderate.^45^ In addition, Triapine analogues with a redox active amino-dimethylphenol moiety at the terminal nitrogen atom of the TSC scaffold showed cytotoxicity in cancer cells with low μM IC_50_ values. The lead analogue (pyridine-2-carboxaldehyde 4-(4-hydroxy-3,5-dimethylphenyl)thiosemicarbazone) quenched Y^**•**^ in mR2 RNR almost as potently as Triapine in the presence of DTT as the reductant.^46^

**Chart 2:**
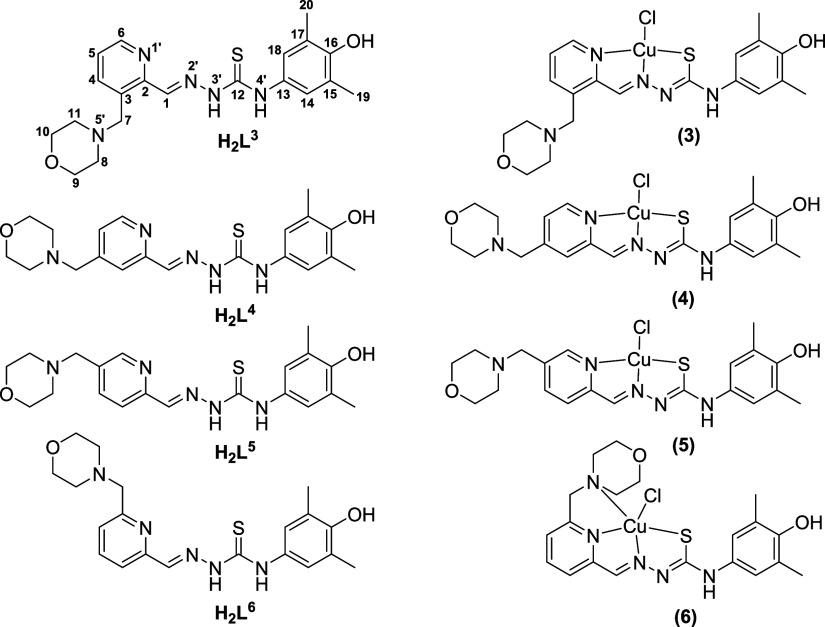
Line drawings of the proligands in neutral form **H**_**2**_**L**^**3**^–**H**_**2**_**L**^**6**^ (the numbers indicate the position of the *N*-methylpmorpholine substituent on the pyridine ring) and their corresponding copper(II) complexes **3**–**6** in solution. The C atom (1–20) and N atom (1′–5′) labeling in the proligands is used for assignment of NMR resonances. It is noteworthy that in the solid-state complexes **3** and **4** exist as [Cu_2_(L^3^)_2_(μ-Cl)_2_]_0.5_[Cu(L^3^)Cl] and [Cu_2_(L^4^)_2_(μ-Cl)_2_], respectively, as crystallographically elucidated

The morpholine unit, which enhances the aqueous solubility of the proligands and shows an improved pharmacological profile,^47,48^ is present in many clinically useful anticancer drugs, e.g., gefitinib and carfilzomib.^49,50^ A synthetic retinoid derivative (fenretinide) bearing an aminophenol moiety was clinically investigated as an anticancer treatment due to its ability to scavenge radicals.^45^ Thus, the α-*N*-heterocyclic TSCs described here and bearing both the morpholine unit and the redox active amino-dimethylphenol unit, as well as the NNS metal binding site in the same molecule, are of particular interest in the search for more efficient anticancer drugs (Chart 2).

This work aimed at (i) the synthesis of new TSCs with the morpholine moiety at each of four available positions of the pyridine ring and incorporating a redox active 2,6-dimethyl-4-aminophenol moiety (Chart 2); (ii) the investigation of the redox behavior and speciation of the proligands **H**_**2**_**L**^**3**^**–H**_**2**_**L**^**6**^, as well as of the thermodynamic stability of copper(II) complexes **3**–**6** in aqueous solution; (iii) the evaluation of the effect of the position of the *N*-methylmorpholine substituent at the pyridine ring (Chart 2) and the impact of copper(II) coordination on the *in vitro* antiproliferative activity in MDR cells; (iv) the investigation of the direct quenching ability of the compounds on tyrosyl radical Y^•^ of mR2 RNR protein and on RNR in cells and on the effect of copper(II) complexes with TSCs on tubulin polymerization; (v) the elucidation of new structure–activity relationships; and (vi) insights into the underlying mechanism of the antiproliferative activity supported by the experimental data and molecular modeling calculations.

## 2 Results and Discussion

### 2.1 Synthesis and Characterization of the Proligands H_2_L^3^–H_2_L^6^

**Scheme 1:**
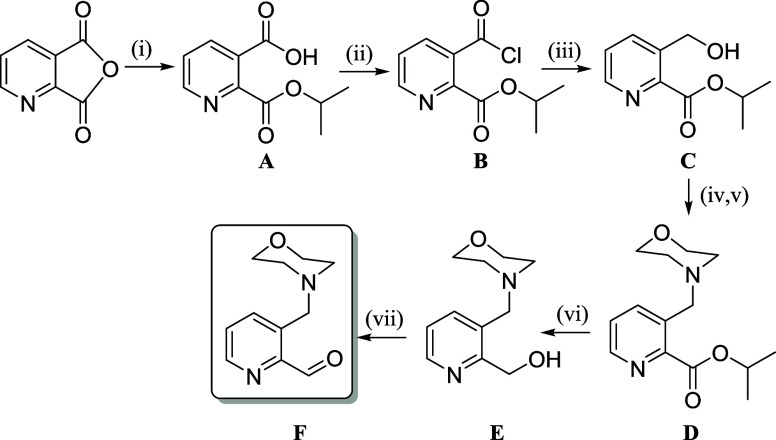
Synthetic Route to 3-(Morpholinomethyl)pyridine-2-carboxaldehyde Reagents: (i) ^*i*^PrOH; (ii) SOCl_2_, DMF, THF; (iii) NaBH_4_, THF (ice bath); (iv) MeSO_2_Cl, Et_3_N, CH_2_Cl_2_; (v) morpholine, CH_2_Cl_2_, CH_3_CN (vi) NaBH_4_, EtOH (ice bath); (vii) SeO_2_, dioxane.

The morpholine-TSC hybrids **H**_**2**_**L**^**3**^**–H**_**2**_**L**^**6**^ were obtained by the condensation reaction of the (appropriate) aldehyde and 4-(4-hydroxy-3,5-dimethylphenyl)thiosemicarbazide^51^ in 58–89% yields. The key precursors in the synthesis, 4-, 5-, and 6-(morpholinomethyl)pyridine-2-carboxaldehyde, were prepared as described previously,^52,45^ while a new seven-step synthetic pathway was used for the synthesis of 3-(morpholinomethyl)pyridine-2-carboxaldehyde (Scheme 1), to avoid lactonization at the pyridine ring. After selective opening of the 2,3-pyridinedicarboxylic anhydride ring in isopropanol (^*i*^PrOH), the carboxyl group in the β-position was converted into the appropriate acyl chloride and reduced to the alcohol. The replacement of the OH group by a morpholine moiety using pretreatment with mesyl chloride, followed by consecutive reduction and oxidation of the α-ester group, afforded the final precursor (Scheme 1 and Experimental Section in the Supporting Information).

^1^H and ^13^C NMR spectra of **H**_**2**_**L**^**3**^**–H**_**2**_**L**^**6**^ in DMSO-*d*_6_ confirmed the formation of the structures shown in Chart 2. The existence of *E* and *Z* isomers was observed for all proligands in solution. This type of isomerism is well documented for similar TSCs^46,53^ and does not affect their pharmacological properties.^54^ The *E*- and Z-isomers of the ligands in solution were identified by the hydrazinic proton chemical shifts: δ 11.39–11.89 for the *E*-isomer and δ 14.23–14.51 for the *Z*-isomer. They are also identifiable by the chemical shift of the proton on the α-carbon on the pyridine ring (adjacent to the pyridyl nitrogen), C6–H: δ 8.49–8.55 for the *E*-isomer and δ 8.69–8.71 for the *Z*-isomer. The *E*/*Z* isomer ratio was estimated by comparison of the integrals of the N^3‘^–protons for **H**_**2**_**L**^**3**^ (2:1), **H**_**2**_**L**^**4**^ (20:1), **H**_**2**_**L**^**5**^ (50:1), and **H**_**2**_**L**^**6**^ (20:1) (Figures S1A–S4E, Supporting Information). The presence of the thione (thio-keto) tautomer is readily observed in ^13^C NMR spectra by the downfield chemical shift of the thiocarbonyl carbon atom, which in this series of ligands lies in the range δ176.14–177.18, comparable with those reported in the literature.^39^

Electrospray ionization mass spectra (ESI-MS) of isomeric proligands **H**_**2**_**L**^**3**^**–H**_**2**_**L**^**6**^ in positive ion mode showed peaks with *m*/*z* 400 and 422, attributed to [M + H]^+^ and [M + Na]^+^, respectively, while in the negative ion mode, the peak with *m*/*z* 398 was assigned to the [M–H^+^]^−^ ion. The 3D structures of **H**_**2**_**L**^**3**^, **H**_**2**_**L**^**5**^, **H**_**2**_**L**^6^, and the doubly protonated proligand **H**_**2**_**L**^**4**^, i.e., **[H**_**4**_**L**^**4**^**]**^**2+**^, were determined by single-crystal X-ray analyses (vide infra).

### 2.2 Synthesis and Characterization of Copper(II) Complexes **3**–**6** (Cocrystallized Solvent Is Omitted in the Formulas Given in the Text)

Complexes **3**–**6** were obtained in good yields (up to 70%) by the reaction of the appropriate TSC proligand with CuCl_2_·2H_2_O in the presence of Et_3_N in a 1:1:1 molar ratio in methanol. The formation of **3**–**6** was confirmed by ESI mass spectra and elemental analysis and, for complexes **4** and **6**, in addition, by HPLC-HR MS (see Figures S5A and S6B in the Supporting Information). In the positive ion mode, a characteristic peak with *m*/*z* 461 was attributed to the [Cu(HL)]^+^ ion (H_2_L = **H**_**2**_**L**^**3**^**–H**_**2**_**L**^**6**^), while in the negative ion mode, the peak with *m*/*z* 495 was attributed to the ion [Cu^II^(L)Cl]^−^. The disappearance of one of the two ν(N–H) absorption bands present in the spectra of **H**_**2**_**L**^**3**^**–H**_**2**_**L**^**6**^ at 3225 and 3188, 3284 and 3216, 3415 and 3165, 3197, and 3123 cm^–1^ indicates the base-assisted tautomerization and deprotonation of the TSC ligands upon coordination to the copper(II) ion in **3**–**6**. The crystals obtained by vapor diffusion of Et_2_O into dimethylformamide (DMF) solution of **3**–**5** or by slow evaporation of methanolic solution of **6** were suitable for SC-XRD analysis.

### 2.3 X-ray Crystallography of the Proligands and Copper(II) Complexes

**Figure 1:**
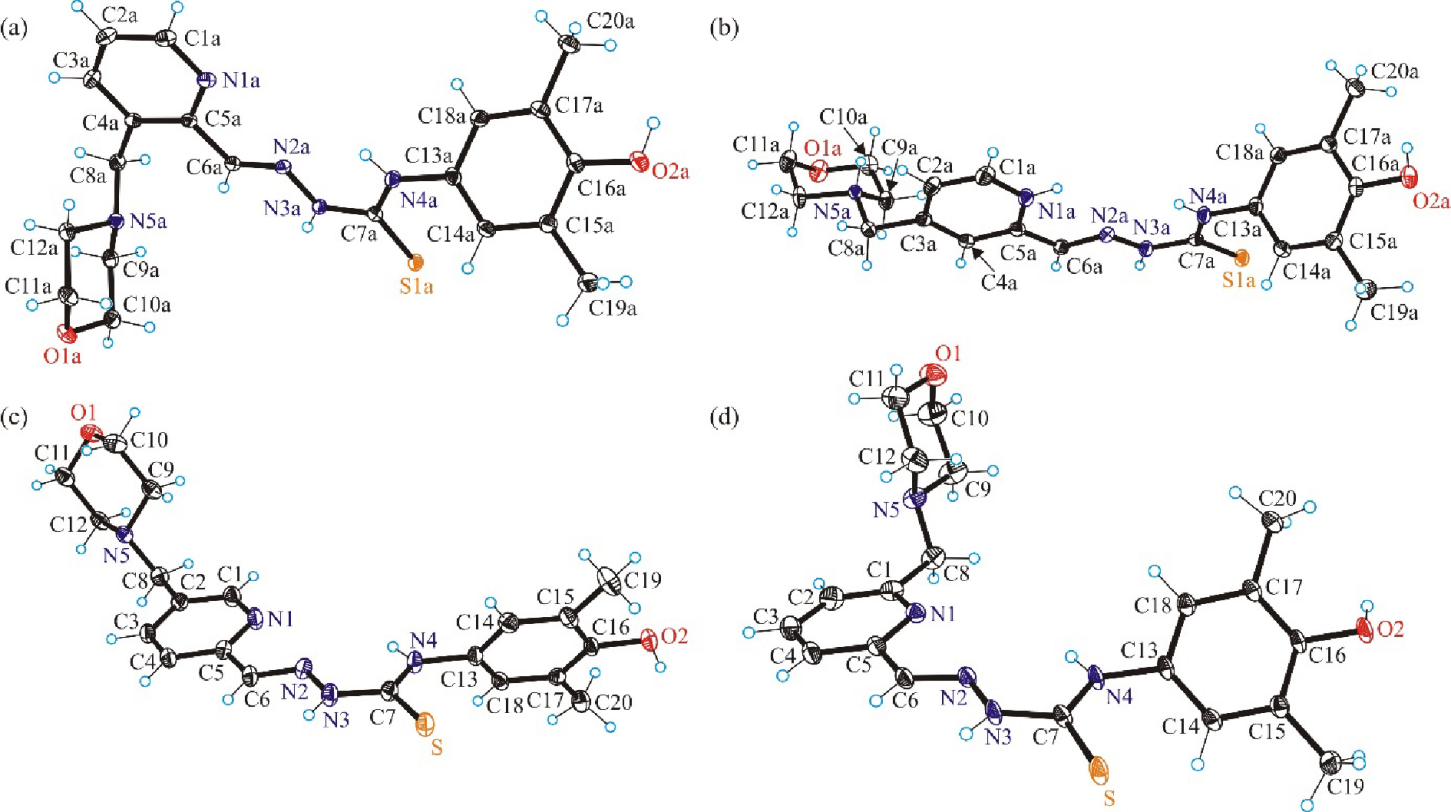
ORTEP views of (a) **H**_**2**_**L**^**3**^, (b) dipositive cation **[H**_**4**_**L**^**4**^**]**^**2+**^; chloride counteranions were omitted, (c) **H**_**2**_**L**^**5**^ and (d) **H**_**2**_**L**^**6**^ with thermal ellipsoids at the 50% probability level. Interstitial solvent molecules present in all four crystal structures were omitted for clarity.

**Figure 2:**
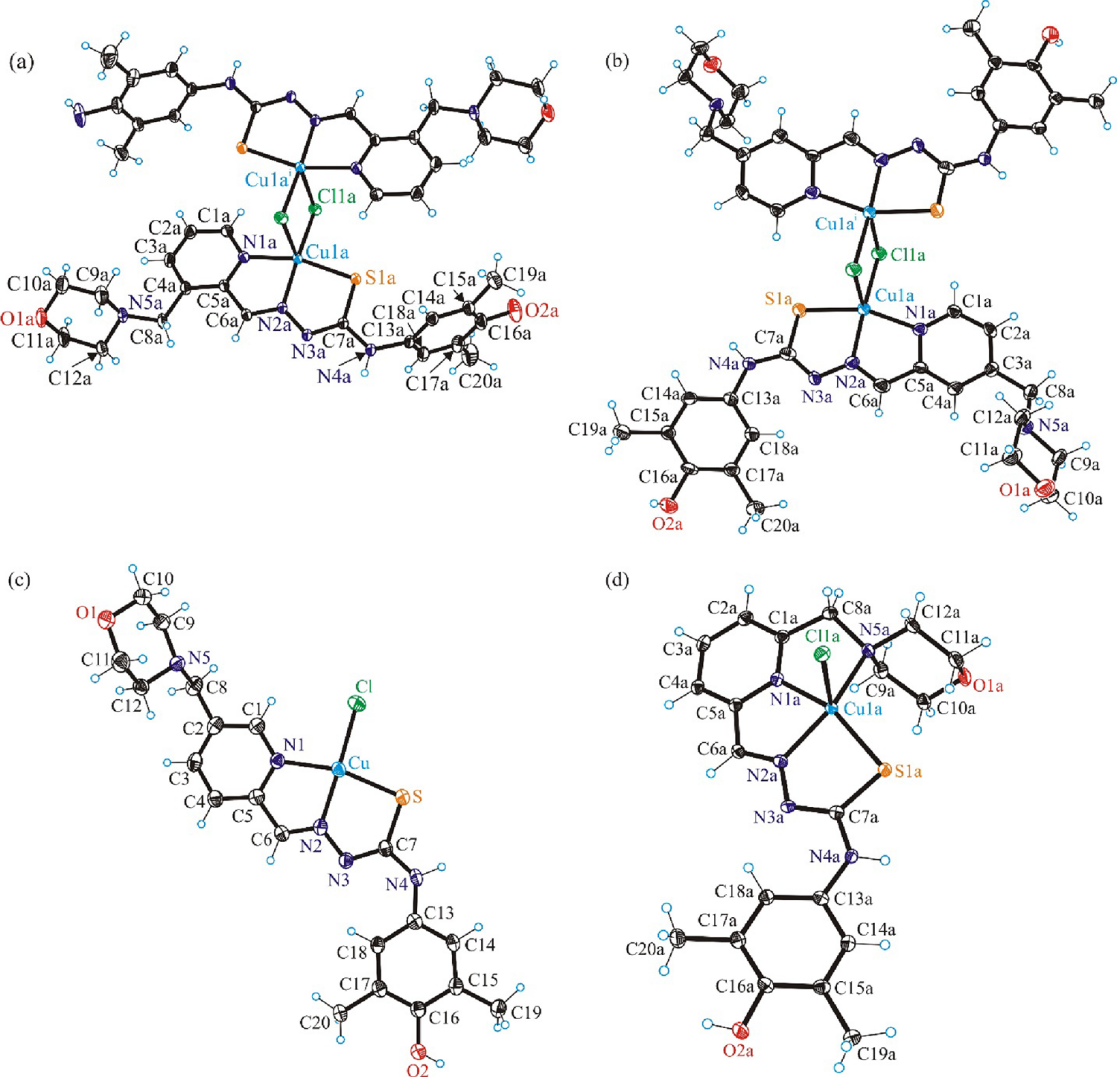
ORTEP views of copper(II) complexes (a) **3**, (b) **4**, (c) **5**, and (d) **6** with thermal ellipsoids at the 50% probability level. Symmetry codes for **3**: (i) −*x* + 1, −*y* + 2, −*z* + 1, and **4**: (i) −*x* + 1, −*y*, −*z* + 2. Interstitial solvent molecules were omitted for clarity. (a) and (b) represent dimers generated from one of the two crystallographically independent mononuclear molecules of **3** and **4**, respectively, while (d) shows one of the two crystallographically independent mononuclear molecules of complex **6**, which are similar (see Table 1 for metrical parameters).

The results of X-ray crystallographic analysis of the hybrids **H**_**2**_**L**^**3**^, **[H**_**4**_**L**^**4**^**]Cl**_**2**_, **H**_**2**_**L^5^**, and **H**_**2**_**L**^**6**^ as well as of their copper(II) complexes **3**–**6** are presented in Figures 1 and 2, while details of data collection and refinement are summarized in Tables S1 and S2 in the Supporting Information. Selected bond lengths and bond angles in **3**–**6** are given in Table 1. The crystallographic asymmetric unit of **H**_**2**_**L**^**3**^ comprises two similar discrete molecules. Likewise, the asymmetric unit of **[H**_**4**_**L**^**4**^**]Cl**_**2**_ consists of two ligand cations and four chloride counteranions.

**Table 1:** Selected Bond Lengths (Å) around Central Cu(II) Ion in Complexes **3**–**6**^a^

complex	**3**		**4**		**5**	**6**	
	molecule A	molecule B	molecule A	molecule B		molecule A	molecule B
bond lengths							
Cu–N1	2.0229(11)	2.0297(12)	2.062(4)	2.038(4)	2.035(3)	1.9573(17)	1.9415(18)
Cu–N2	1.9732(11)	1.9659(11)	1.968(4)	1.965(4)	1.964(3)	1.9933(18)	1.9960(18)
Cu–N5						2.1790(18)	2.1604(18)
Cu–S	2.2442(4)	2.2452(4)	2.2901(14)	2.2658(14)	2.2545(12)	2.2775(6)	2.2565(6)
Cu–Cl	2.2572(4)	2.2102(4)	2.2476(13)	2.2574(13)	2.2151(13)	2.4834(6)	2.6672(6)
Cu–Cl^i^	2.6742(4)		2.7102(15)	2.7378(14)			
Bond angles							
N1–Cu–S	162.09(3)	164.11(4)	159.21(12)	162.49(12)	163.43(9)		
N2–Cu–Cl	170.35(3)	178.41(4)	171.96(13)	167.52(13)	178.03(9)		
N1–Cu–N2	80.88(4)	80.49(5)	80.27(16)	80.82(16)	80.95(12)	78.91(7)	79.85(7)
N1–Cu–Cl	98.21(3)	98.95(4)	98.00(11)	96.90(12)	97.23(9)	96.96(5)	87.64(5)
N2–Cu–S	84.26(3)	84.44(3)	82.07(12)	83.48(12)	83.89(9)		
S–Cu–Cl	94.755(15)	96.238(17)	97.97(5)	96.75(5)	98.06(5)	102.04(2)	108.284(19)
N1–Cu–N5						78.96(7)	79.53(7)
N2–Cu–N5						152.32(7)	155.15(7)
N2–Cu–Cl						105.49(5)	104.32(5)
N5–Cu–Cl						93.52(5)	88.53(5)

a Symmetry code: for molecule **3A**—(i) −*x* + 1, −*y* + 2, −*z* + 1; for molecule **4A**—(i) −*x* + 1, −*y*, −*z* + 2; for molecule **4B**—(i) −*x* + 1, −*y*, −*z* + 1.

All TSCs adopt in the solid state the *E* configuration relative to the C6=N2 (or C6a=N2a) double bond. The range of the Schiff-base (imine) C=N distances is 1.278–1.286 Å, as expected.^55−57^ The ligands were isolated in the thione (thio-keto) tautomeric form as demonstrated by the C=S distances, which range from 1.668 to 1.695 Å, in agreement with literature values.^58,59,45^ In contrast to **H**_**2**_**L**^**3**^, **H**_**2**_**L**^5^, and **H**_**2**_**L**^**6**^, **H**_**2**_**L**^**4**^ was crystallized as the fully protonated species **[H**_**4**_**L**^**4**^**]Cl**_**2**_ with two additional protons, one at the morpholine nitrogen atom N5a and the second at the pyridine nitrogen N1a, in agreement with speciation in aqueous solution. The studies in solution showed that the neutral molecules **H**_**2**_**L**^**3**^**–H**_**2**_**L**^**6**^ are protonated with decreasing pH at the pyridine and morpholine nitrogens (see Section 2.5). In all four ligands, the thiocarbonyl group points away from the pyridyl and imine nitrogen donor atoms. The morpholine moiety adopts the chair conformation.

The asymmetric unit of complex **3** consists of two crystallographically independent molecules A and B of the copper(II) complex of the general formula [Cu(L^3^)Cl]. In the crystal, molecule **3A** forms a centrosymmetric dimer, the structure of which is shown in Figure 2a. Each copper(II) ion has a distorted square-pyramidal coordination geometry (τ_5_ = (β–α)/60° = 170.35–162.09/60 = 0.14, τ_5_ = 0 for square-pyramidal polyhedron, and τ_5_ = 1 for a trigonal–bipyramidal coordination geometry).^60^ In contrast, the molecule **3B** is four-coordinate and is not involved in any bonding interactions. The structure of molecule **3B** is similar to that of complex **5** in Figure 2c and is shown in Figure S7 in the Supporting Information. The calculated τ′_4_ parameter^61^ for **3B** is 0.08, which is consistent with a slightly distorted square-planar coordination geometry. The best empirical formula for **3** in the solid state consistent with CIF and checkCIF can be written as follows: [Cu_2_(L^3^)_2_(μ-Cl)_2_]_1/2_[Cu(L^3^)Cl]. The asymmetric unit of complex **4** also consists of two crystallographcally independent molecules of complexes [Cu(L^4^)Cl] A and B. In the crystal, in contrast to complex **3**, each of these two molecules forms centrosymmetric dimers, and, therefore, the best formula of **4** in agreement with CIF and checkCIF is [Cu_2_(L^4^)_2_(μ-Cl)_2_]. In dichlorido-bridged complexes of molecules **3A**, and also in complex **4**, the copper(II) ions adopt the five-coordinate geometry (τ_5_ = 0.21 for **4A** and 0.08 for **4B**),^60^ in which two shared chloride coligands occupy at the same time the axial position of the one Cu(II) ion and one of the four equatorial positions of another Cu(II) ion, as shown on the left side of Figure S8.^62−64^

It should also be noted that the apical intradimer Cu–Cl bond(s) is (are) significantly longer than the equatorial Cu–Cl bond(s), indicating that the association is not very strong and the complexes should not be robust in solution. This was indeed confirmed by speciation studies in solution (vide infra), which showed that the dimers dissociate into monomeric entities. Hence, from the observed solution chemistry and the medicinal chemistry perspective, complexes **3** and **4** can be represented as four-coordinate mononuclear structures in solution (Chart 2).

The asymmetric units of complexes **5** and **6** consist of one square-planar molecule (Figure 2c) and two crystallographically independent square-pyramidal molecules, one of which is shown in Figure 2d.

The TSC-morpholine hybrids **H**_**2**_**L**^**3**^–**H**_**2**_**L**^**5**^ in complexes **3**–**5** act as tridentate monoanionic ligands binding to Cu(II) via pyridine nitrogen atom N1 (N1a), hydrazinic nitrogen N2 (N2a), and the thiolato sulfur atom, while the donor capacity of **H**_**2**_**L**^**6**^ is increased by additional coordination of the morpholine nitrogen atom N5a to Cu(II). The slightly distorted square-planar coordination geometry of Cu(II) in **5** is completed by a chlorido coligand (τ′_4_ = 0.09).^61^ The coordination number 5 in complex **6** is achieved by additional coordination of one chlorido coligand. The coordination polyhedron is approximately described as square-pyramid. For the two crystallographically independent square-pyramidal molecules of **6** τ_5_ = 0.08 and 0.07,^60^ respectively.

In all four copper(II) complexes, the TSCs have undergone tautomerization accompanied by deprotonation to produce the uninegative thio-enolato anionic ligands. This is evidenced by the lengthening of the carbon–sulfur bonds [C–S bonds: 1.731–1.767 Å].

Characterization of the new TSCs and their copper(II) complexes by ^1^H and ^13^C NMR spectroscopy, ESI mass spectrometry, and SC-XRD confirmed their expected composition and structure both in the solid state and in solutions of organic solvents. However, the reactivity of the prepared compounds and their behavior in aqueous solution along with their redox properties are of importance for understanding their pharmacological potential. Therefore, the behavior in aqueous solution of selected TSC–morpholine hybrids and of their copper(II) complexes were studied in detail.

### 2.4 UV–vis and EPR Spectra of Complexes **3**–**6**

The electronic absorption spectra of **3**–**6** in methanol showed high intensity bands in the UV region at 250–300 nm due to π → π* and *n*–π transitions and also an intense band at 420–428 nm, which is assigned to the S → Cu^II^ transitions (LMCT).^40^ Spin-allowed but Laporte-forbidden d–d transitions are seen in the visible region of the spectrum at 610–650 nm with molar absorptivities around 300 M^–1^cm^–1^ as a shoulder of the mentioned LMCT band. The spectra measured in DMSO and water/DMSO 2:1 showed only small shifts of absorption maxima due to solvatochromic effects (see Figure S9 for complexes **6** and **3**).

The UV–vis spectra of **3**–**6** in methanol are shown in Figure S10a, while the X-band EPR spectra of **3**–**6** in methanol at 100 K are shown in Figure S10b in the Supporting Information. The axial signals of **3**–**5** were simulated with very similar Spin Hamiltonian parameters, which are summarized in Table S3. According to Hathaway’s analysis, such a spectrum indicates elongated octahedral or square-planar symmetry with a half-occupancy of the copper(II) d_*x*2–*y*2_ orbital in the ground state.^65^ Close similarity of the spectra of **3**–**5** indicates that, in solution, **3A** and **4** do not preserve their dimeric solid-state structure, in accordance with optical spectra for these complexes, in which the main absorption bands have comparable extinction coefficients. Complex **6** under analogous conditions revealed slightly smaller hyperfine coupling constants. EPR spectra of **6** measured in methanol, DMSO, and/or water indicate that this mononuclear complex remains intact in the presence of these coordinating solvents (see Figure S11).

### 2.5 Proton Dissociation Processes and Lipophilicity of the Proligands

The fully protonated forms of the proligands **H**_**2**_**L**^**3**^**–H**_**2**_**L**^**6**^ possess four proton dissociable groups (with general formula **H**_**4**_**L**^**2+**^), namely, the pyridinium-NH^+^ and hydrazonic-NH of the TSC scaffold, in addition to the morpholinium–NH^+^ and phenolic–OH function, as also revealed in the SC-XRD structure of **[H**_**4**_**L**^**4**^**]Cl**_**2**_ (Figure 1b). Accurate determination of proton dissociation constants was hindered by (i) moderate solubility of the TSC-morpholine hybrids in water, (ii) pH-dependent isomerization of the proligands in solution, (iii) side reactions in the basic pH range, e.g., oxidation of the 2,6-dimethyl-4-aminophenol moiety at pH > 10, and (iv) overlapping deprotonation of the phenolic–OH and hydrazonic–NH groups at pH > 10. Nevertheless, proton dissociation of the hybrids studied in 30% (v/v) DMSO/H_2_O by a combination of pH–potentiometric titration and ^1^H NMR and UV–vis spectroscopies (for details, see the Supporting Information and Figures S12 and S13 therein) delivered the proton dissociation constants (p*K*_a_) quoted in Table 2.

**Table 2:** Proton Dissociation Constants (p*K*_a_) of the Studied Proligands by Different Methods (30% (v/v) DMSO/H_2_O; *I* = 0.1 M (KCl); *t* = 25 °C)

	**method**	**H**_**2**_**L**^**3**^	**H**_**2**_**L**^**4**^	**H**_**2**_**L**^**5**^	**H**_**2**_**L**^**6**^
p*K*_a1_	pH–potentiometry	<2	<2	<2	<2
p*K*_a2_	pH–potentiometry	5.62 ± 0.06	5.16 ± 0.04^a^	5.57 ± 0.05	5.64 ± 0.03
p*K*_a3_	pH–potentiometry	>10.5	∼ 10.5	∼10.5	∼10.6
p*K*_a1_	UV–vis spectrophotometry	1.53 ± 0.01	1.48 ± 0.01	1.16 ± 0.01	<0.8

a p*K*_a2_ = 5.2 ± 0.1 determined by ^1^H NMR titrations (*c*_L_ = 1 mM; *I* = 0.1 M (KCl); *t* = 25 °C; 30% (v/v) DMSO-*d*_6_/H_2_O).

**Scheme 2:**
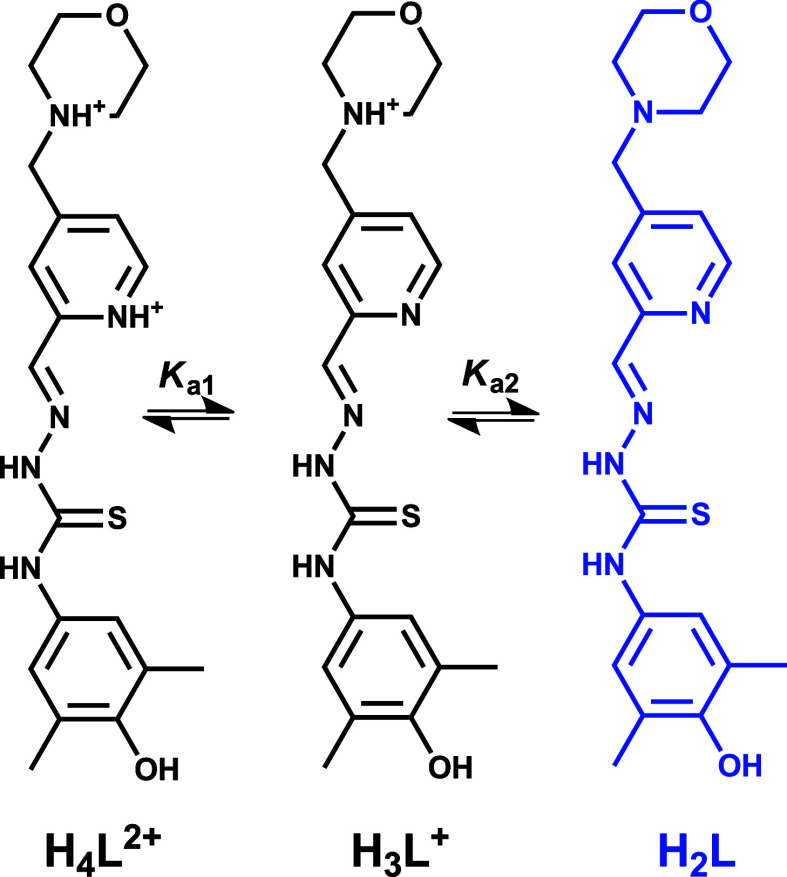
First Two Deprotonation Steps of **[H**_**4**_**L**^**4**^**]**^**2+**^ at pH < 10

Based on these data, the deprotonation steps at pH < 10 for **[H**_**4**_**L**^**4**^**]Cl**_**2**_ shown in Scheme 2 seem reasonable.

In addition, we concluded that all proligands studied in this work are present in solution in their neutral form (H_2_L) at physiological pH. Deprotonation of the morpholinium–NH^+^ moiety at physiological pH contributes markedly to their fairly lipophilic character. Even though attempts to accurately determine the distribution coefficients (*D*_7.4_) by the traditional *n*-octanol/water partitioning failed, a lower limit (log *D*_7.4_ >+2) was estimated, as all of the proligands remained in the nonpolar phase.

### 2.6 Solution Speciation and Stability of Copper(II) Complexes

The solution speciation of copper(II) complexes with **H**_**2**_**L**^**3**^**–H**_**2**_**L**^**6**^ was studied primarily by UV–vis spectrophotometric titrations in a 30% (v/v) DMSO/H_2_O solvent mixture as a function of pH. The determined p*K*_a_ values for complexes **3**–**6** are shown in Table 3. It was also found that between pH 5.5 and 8.7 (for the Cu(II)–**H**_**2**_**L**^**3**^ system) and at about pH 7 (in the case of the other three proligands), predominant formation of the monocationic complexes [Cu(HL)]^+^ was observed. Other details of this investigation can be found in the Supporting Information (see also Figure S14 and Chart S1).

**Table 3:** Conditional Stability Constants (log *K*′_5.9_) Determined from EDTA Displacement Studies in Water or in 30% (v/v) DMSO/H_2_O by UV–vis Spectrophotometry and p*K*_a_ Values of the Copper(II) Complexes in 30% (v/v) DMSO/H_2_O; *t* = 25 °C, pCu (=–log[Cu(II)]) Values Calculated at pH 5.9 (*c*_L_ = *c*_Cu(II)_ = 1 μM) in Addition to the Observed Rate Constants (*k*_obs_) Obtained for the Redox Reaction of the Complexes with GSH (pH = 7.4 (50 mM HEPES); *c*_complex_ = 25 μM; *c*_GSH_ = 1.25 mM; Pure Water) (*I* = 0.1 M (KCl); *t* = 25 °C)

	**method**	**3**	**4**	**5**	**6**
log*K*′_5.9_^a^(H_2_O)	UV–vis spectrophotometry	13.82 ± 0.04	13.35 ± 0.02	13.39 ± 0.01	13.73 ± 0.02
log*K*′_5.9_^b^ (DMSO/H_2_O)	UV–vis spectrophotometry	10.33 ± 0.01	10.07 ± 0.01	9.99 ± 0.01	10.09 ± 0.04
p*K*_a_**[Cu(H**_**2**_**L)]**^**2+**^	UV–vis spectrophotometry	4.23 ± 0.01	4.48 ± 0.01	4.75 ± 0.01	n.d.
p*K*_a_**[Cu(H**_**2**_**L)]**^**2+**^	pH potentiometry	n.d.	4.60 ± 0.02	4.95 ± 0.04	n.d.
pCu_5.9_	calcd.	8.20	8.06	8.02	8.07
*k*_obs_ (min^–1^)	UV–vis spectrophotometry	0.059 ± 0.004	0.079 ± 0.019	0.058 ± 0.010	0.165 ± 0.011

a p*K*_a_ of EDTA and logβ of its Cu(II) complex were taken from the literature,^66^ and log β′_5.9_ = 13.89 was calculated for [Cu(EDTA)]^2–^ in pure water.

b Log β′_5.9_ = 10.19 for the Cu(II)–EDTA complex in the 30% (v/v) DMSO/H_2_O mixture was determined via Triapine displacement reaction.^67^

**Figure 3:**
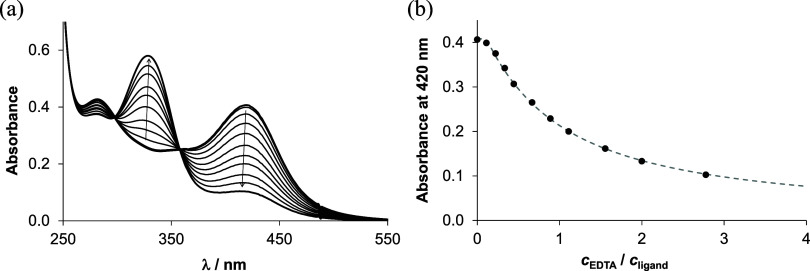
(a) UV–vis spectra for the copper(II)–**H**_**2**_**L**^**4**^ (1:1) system with an increasing concentration of EDTA. (b) Measured (filled circle) and fitted (dashed line) absorbances at 420 nm plotted against the EDTA-to-ligand ratio (*c*_L_ = 25 μM, *c*_Cu_ = 25 μM, *c*_EDTA_ = 0–62.5 μM, 30% (v/v) DMSO/H_2_O; pH = 5.90, *I* = 0.1 M (KCl), *t* = 25 °C).

The stability constants of **3**–**6** were determined by EDTA competition experiments monitored by UV–vis spectrophotometry at pH 5.9, where the [Cu(HL)]^+^ species is dominant. Since the displacement of the TSC ligand by EDTA is relatively slow, a 2 h reaction time was used to reach equilibrium. Upon increasing the concentration of EDTA, the absorbance of the characteristic S → Cu charge transfer band at 400–416 nm decreased (see, for example, Figure 3 for the copper(II)–**H**_**2**_**L**^**4**^ (1:1) system).^68^ The experiments were performed in both aqueous solution and the 30% (v/v) DMSO/H_2_O solvent mixture. The determined conditional (apparent) formation constants (log *K*′_5.9_) are rather similar (see Table 2) in both media. However, the values are lower in the DMSO/H_2_O solvent mixture than in aqueous solution, presumably due to coordination of DMSO to copper(II). An analogous observation was reported for the copper(II) complex of Triapine.^67^ It should also be mentioned that the Cu(II) complexes **3**–**6** showed somewhat higher log *K*′_5.9_ values compared with the Triapine complex in 30% (v/v) DMSO/H_2_O (log *K*′_5.9_ = 9.47 was calculated by using experimental data reported previously^69^). To compare the copper(II) binding ability of the studied TSCs **H**_**2**_**L**^**3**^–**H**_**2**_**L**^**6**^ at pH 5.9 (at which the conditional constants were determined), pCu (−log [Cu(II)]) values were also computed using the experimentally determined equilibrium constants (Table 2). The higher pCu value indicates a stronger metal ion binding ability of the ligand under given circumstances. The ligands form complexes with fairly similar stability, in agreement with the conditional stability constants. Interestingly, the additional coordination of the morpholine nitrogen in the copper(II) complex of **H**_**2**_**L**^**6**^ does not result in higher thermodynamic stability of **6** compared to **3**–**5**. A similar behavior was reported for aqueous solutions of the *N*-terminally dimethylated derivative of the 2-pyridinecarboxaldehyde TSC (PTSC)^69^ and for the corresponding morpholine-hybrid Morph-dm-FTSC.^52^ In particular, the calculated pCu values were also very close, 13.17 for PTSC vs 13.08 for Morph-dm-FTSC calculated at pH 7.4. Thus, the increase of ligand denticity did not lead to enhanced thermodynamic stability.

Since the anticancer activity of the copper(II) complexes of TSCs is often related to their redox reaction with cellular thiols such as glutathione (GSH),^70^ we further investigated the reactions of **3**–**6** with this reductant.

### 2.7 Reduction of Copper(II) Complexes by Glutathione

**Figure 4:**
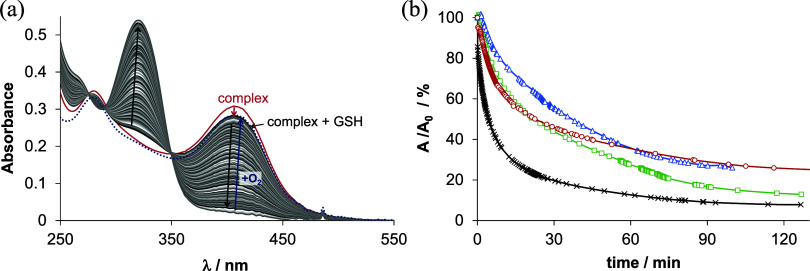
(a) Time-dependent UV–vis absorption spectra of complex **6** (copper(II)–**H**_**2**_**L**^**6**^ (1:1) system) in the presence of GSH (50 equiv) before (red line) and after mixing the solutions (black/gray lines) in a tandem cuvette under anoxic conditions. The absorbance changes after bubbling oxygen into the reaction mixture (blue line). (b) Plot of the time-dependent absorbance changes at 406 nm for **6** (cross, black line), at 396 nm for **4** (square, green line), at 398 nm for **5** (circle, red line), and at 398 nm for **3** (triangle, blue line) (50 mM HEPES, pH = 7.4); solvent: water; *c*_Cu_ = *c*_L_ = 25 μM; *c*_GSH_ = 1.25 mM; *I* = 0.1 M (KCl); *t* = 25 °C).

The direct reduction of the copper(II) complexes with the antioxidant GSH was investigated in anoxic aqueous solution at pH 7.4 by UV–vis spectrophotometry. At this pH, the complex [Cu(HL)]^+^ is assumed to predominate according to the speciation studies (*vide supra*). The spectral changes were monitored in the wavelength range 250–550 nm by using a large excess of GSH (50 equiv). In this wavelength range, the spectral changes are due to the absorption of the metal complex and the ligand. In addition, ascorbic acid, a weaker reductant than GSH, was also tested, but the reaction was very slow, suggesting that these copper(II) complexes cannot be reduced efficiently by ascorbic acid. In contrast, remarkable spectral changes were detected in the case of GSH, as can be seen for complex **6** (copper(II)–**H**_**2**_**L**^**6**^ (1:1) system) in Figure 4a.

The first recorded spectrum after mixing the complex **6** and GSH showed minor shifts of the absorbance bands most likely due to the formation of a ternary complex with GSH, as also reported for other TSC complexes.^71,72^ A significant decrease in absorbance was observed at λ_max_ 406 nm, while the absorbance increased at the λ_max_ of the metal-free ligand (∼316 nm). These changes imply that, after reduction in the presence of excess GSH, the generated copper(I) complex is not stable and liberates the free TSC ligand. Upon purging the solution with oxygen, the copper(II) complex was regenerated in all cases (see Figure 4a for **6**), which suggests a reversible redox process. In order to obtain comparable data, the recorded absorbance–time curves were further analyzed, primarily at the λ_max_ of the complex. The time-dependent absorbance changes are shown for all the studied systems in Figure 4b. The observed rate constants (*k*_obs_) were calculated (Table 2) as a semiquantitative description of the reaction kinetics. These collected data indicate that the studied copper(II) complexes can be reduced by GSH at a similar rate, except that complex **6** can be reduced somewhat faster compared to **3**–**5**. The obtained *k*_obs_ values fall into the range for Triapine (0.10 min^–1^), 2-pyridinecarboxaldehyde TSC (0.041 min^–1^), and *N*-monomethylated *N*-methyl-Triapine (0.077 min^–1^), measured under similar conditions.^67^ To find out whether this slightly different kinetics for **6** and **3**–**5** is in accordance with their reduction potentials, cyclic voltammetry was performed.

### 2.8 Electrochemistry and Spectroelectrochemistry

**Figure 5:**
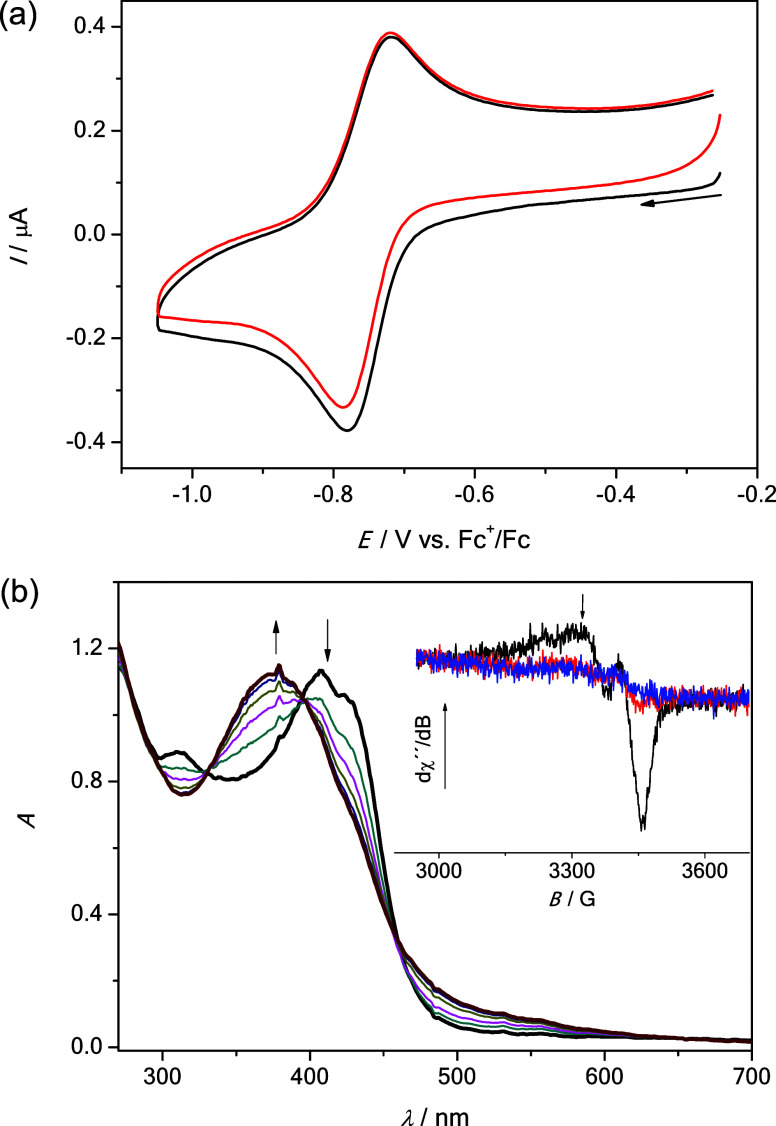
(a) CVs of **6** (black trace—first scan, red trace—second scan) in DMSO/*n*-Bu_4_NPF_6_ at the Pt working electrode at a scan rate of 100 mV s^–1^. (b) UV–vis spectra measured upon cathodic reduction of **6** at the first reduction peak by using a honeycomb Pt working electrode (inset: (black trace) EPR spectrum of **6** in DMSO; (red and blue trace) EPR spectra of **6** in DMSO after cathodic reduction at the first cathodic peak.)

The redox properties of the copper(II) complexes **3**–**6** were investigated by electrochemical, EPR, and UV–vis spectroelectrochemical measurements. Cyclic voltammograms (CVs) of copper(II) complexes in dimethyl sulfoxide (DMSO) with platinum or glassy-carbon working electrode at a scan rate of 100 mV s^–1^ were very similar in the cathodic part exhibiting one reversible reduction peak, as shown in Figure 5a for **6**. While the half-wave reduction potentials *E*_1/2_^red^ for complexes **3**–**5** were almost the same (−0.82 V vs Fc^+^/Fc), the *E*_1/2_^red^ for **6** is by 0.07 V more positive (−0.75 V vs Fc^+^/Fc), as summarized in Table 4. The corresponding proligands are not redox active in the cathodic part (not shown).

**Table 4:** Redox Potentials (vs Fc^+^/Fc) of the Copper(II) Complexes **3**–**6** in DMSO/*n*-Bu_4_NPF_6_ at a Scan Rate of 100 mV s^–1^^a^

Cu(II) complexes	*E*_1/2_^red^ (V)	*E*_pa_^1^ (V)	*E*_pa_^2^ (V)
**3**	–0.83	+0.27	+0.79
**4**	–0.82	+0.31	+0.77
**5**	–0.82	+0.29	+0.69
**6**	–0.75	+0.26	+0.66

a *E*_1/2_^red^—the first reduction half-wave potential, *E*_pa_^1^—the first anodic peak potential, and *E*_pa_^2^—the second anodic peak potential.

Therefore, this slightly more positive reduction potential for complex **6** indicated that it can be reduced more easily, in agreement with the GSH reduction reaction kinetics described previously.

To confirm that the biologically accessible reduction is metal-centered, the nearly reversible one-electron reduction of **6** in DMSO was studied *in situ* by UV–vis spectroelectrochemistry. A new absorption band at 375 nm arose upon cathodic reduction of **6** in DMSO at the first electron transfer with a simultaneous decrease of the initial optical band at 407 nm *via* isosbestic points at 395 and 460 nm (Figure 5b). A strong decrease of the initial EPR signal originating from Cu(II) (*S* = 1/2) was observed in the analogous spectroelectrochemical experiment directly in the EPR cavity by using a large platinum working electrode and a flat spectroelectrochemical cell (see inset in Figure 5b), thus confirming the reduction of Cu(II) with formation of a diamagnetic d^10^ EPR-inactive Cu(I) (*S* = 0) species. It should be noted that these spectral changes are different from those that resulted upon addition of GSH to **6** (Figure 4), since in the latter case the Cu(I)-GSH complex is formed, while in the case of the cyclic voltammetric studies the reduction was induced electrochemically.

In the anodic part of the CVs, two dominating oxidation waves were observed for all copper(II) complexes, with the data shown for **3** as an example (Figure S15a). The first oxidation peak at around +0.32 V vs Fc^+^/Fc was attributed to the two-electron oxidation of the TSC scaffold, as reported recently for Triapine analogues,^46^ and the height of the anodic peak was approximately twice that of the cathodic peak found upon one-electron cathodic reduction (see Figure S15b). The irreversible oxidation of the ligand in **3** was confirmed by UV–vis spectroelectrochemistry, where the irreversible changes of UV–vis spectra were observed at the first oxidation peak with a decrease of the initial optical band at 430 nm and increase of the band at 290 nm *via* the isosbestic point at 386 nm in DMSO (Figure S16). The second oxidation process at around +0.8 V vs Fc^+^/Fc (see Figure S15a) indicated further irreversible multielectron oxidation of the ligand, which likely leads to the formation of a species similar to those reported for Triapine analogues.^46^

In general, to exhibit an antiproliferative effect, the Cu-TSC complexes must be reduced by intracellular reductants to be able to redox cycle between two oxidation states (Cu^II^↔Cu^I^) in the biologically accessible window of potentials (−1.04 to +0.16 V vs Fc^+^/Fc). As the Cu^II^/Cu^I^ redox activity of the investigated complexes (*E*_1/2_^red^ around −0.8 V vs Fc^+^/Fc) fits this window, their antiproliferative activity in cancer cell lines and their ability to generate reactive oxygen species (ROS) were further investigated.

### 2.9 Cytotoxicity of the Proligands and Their Copper(II) Complexes

The cytotoxic activity of proligands **H**_**2**_**L**^**3**^**–H**_**2**_**L**^**6**^ and their copper(II) complexes **3**–**6** was investigated in the MES-SA (human uterine sarcoma) and in its MDR counterpart (MES-SA/Dx5) cell lines by a fluorescent protein-based assay.^73^ The IC_50_ values obtained are summarized in Table 5.

**Table 5:** *In Vitro* Cytotoxicity Data (IC_50_ μM ± SD; *n* = 3) Determined for the Proligands and Their Copper(II) Complexes in MES-SA and MES-SA/Dx5 Cells in the Absence and Presence of 0.4 μM Tariquidar (TQ)^a^

**IC**_**50**_**/μM**	**MES-SA**	**MES-SA/Dx5**	**MES-SA + TQ**	**MES-SA/Dx5 + TQ**	**SR**	**SR (TQ)**
**H**_**2**_**L**^**3**^	2.9 ± 0.4	13.1 ± 3.2	3.5 ± 0.8	3.9 ± 2	0.22	0.89
**3**	15 ± 3	5.5 ± 0.6	11.6 ± 3.1	3.2 ± 1	2.76	3.58
**H**_**2**_**L**^**4**^	5.2 ± 0.8	9.9 ± 0.4	5.3 ± 0.8	4.2 ± 1	0.49	1.25
**4**	19.2 ± 1.7	5.8 ± 0.1	17.4 ± 3.7	4.9 ± 1	3.33	3.53
**H**_**2**_**L**^**5**^	4.0 ± 0.7	8.6 ± 0.9	4.9 ± 0.3	5.2 ± 1	0.46	0.94
**5**	31.0 ± 6.2	8.1 ± 2	29.0 ± 2.7	7.7 ± 3	3.81	3.76
**H**_**2**_**L**^**6**^	0.25 ± 0.05	4.2 ± 0.8	0.27 ± 0.04	0.39 ± 0.05	0.06	0.69
**6**	0.29 ± 0.04	1.4 ± 0.2	0.29 ± 0.04	0.39 ± 0.1	0.21	0.75
**Triapine**	0.52 ± 0.1	2.8 ± 0.5	0.49 ± 0.02	0.67 ± 0.2	0.18	0.72
**Cu(II)-Triapine**^b^	2.4	16.9	n.d.	n.d.	0.14	–
**Doxorubicin**	0.04 ± 0.01	1.9 ± 0.6	0.03 ± 0.01	0.02 ± 0.01	0.02	1.50

a SR and SR (TQ): selectivity ratio IC_50 (MES-SA)_/IC_50 (MES-SA/Dx5)_ in the absence and in the presence of TQ, respectively

b Taken from Hager et al.^67^

Since the resistance of MES-SA/Dx5 cells is mainly mediated by Pgp,^67^ experiments were also performed in the presence of Pgp inhibitor tariquidar (TQ). As compared to Triapine, **H**_**2**_**L**^**3**^– **H**_**2**_**L**^**5**^ were five- to ninefold less toxic to the parental MES-SA cells, showing even weaker activity against MDR MES-SA/Dx5 cells. Interestingly, MES-SA/Dx5 cells proved to be more sensitive to **3**–**5** (SR > 2) than MES-SA cells. Characterization of **H**_**2**_**L**^**6**^ revealed a different pattern. As compared with Triapine, this ligand proved to be more toxic in both the parental and MES-SA/Dx5 cells, without any significant effect of complex formation with copper(II). Taken together, the *in vitro* cytotoxicity assays revealed that the proligands possess significant antiproliferative activity, which is, however, blunted in MDR cells. Strikingly, MDR cells proved to be overly sensitive to **3**–**5**, but assays performed in the presence of the Pgp inhibitor TQ indicated that this paradoxical hypersensitivity is not dependent on the function of Pgp.

Given the results of electrochemical investigations showing that complexes **3**–**6** can be reduced and reoxidized in the biologically accessible redox potential range in cells and the ability of the compounds to be reduced by GSH, it was plausible to assume that Cu(I) would reoxidize to Cu(II) and thereby promote Fenton reactions intracellularly to cause ROS accumulation.^74^ Therefore, ROS generation of the copper(II) complexes was investigated in cell-free conditions and in live cells.

### 2.10 Investigation into the Mechanism of Action

#### 2.10.1 Cell-Free ROS Generation by the Copper(II) Complexes

First, we studied the ability of complexes **3**–**6** to generate ROS in solution in the presence of H_2_O_2_. ROS generation was monitored by an EPR spin trapping technique.^75^ As formation of hydroxyl radicals *via* the Fenton reaction requires H_2_O_2_, the aqueous solution of the complexes was mixed with 5,5-dimethyl-1-pyrroline-*N*-oxide (DMPO, a spin trapping agent) under aerobic conditions and the EPR spectra were recorded 2 min after the addition of the H_2_O_2_. The results showed that even low concentrations of the complexes (8 μM) initiated the generation of ROS, as confirmed by the immediate and continuous increase of the characteristic four-line EPR signal assigned to the ^•^DMPO–OH spin adduct (Figure S17, Supporting Information).

#### 2.10.2 ROS Generation in Cells

ROS accumulation was evaluated in both the MES-SA and MES-SA/Dx5 cell lines by using 2′,7′-dichlorodihydrofluorescein diacetate (H_2_DCF-DA) as a probe. This compound enters the cells by passive diffusion, and after oxidation, it is converted to the highly fluorescent 2′,7′-dichlorofluorescein (DCF), enabling the estimation of intracellular ROS levels. ROS generation was measured after a 4 h treatment with 25 μM **H**_**2**_**L**^**6**^ or **6**; *tert*-butylhydroperoxide (TBHP, 25 μM) was used as a positive control.

**Figure 6:**
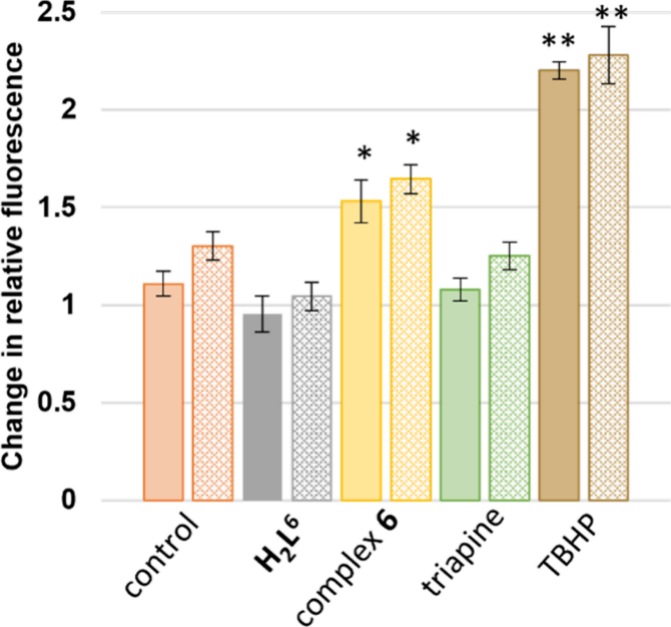
Intracellular ROS generation measured by the DCF-DA assay in MES-SA (filled bars) and MES-SA/Dx5 (lattice bars) cells, after a 4 h treatment with the indicated compounds at 25 μM: **H**_**2**_**L**^**6**^ (gray), complex **6** (yellow), Triapine (green), and TBHP (brown). Values indicate fold change relative to the fluorescence of the cells treated with fluorescent probe alone (orange bars), following normalization to the initial fluorescence at the beginning of the incubation. Paired *t* test to the medium control was calculated; *P < 0.5; **P < 0.01. Values were calculated from at least three independent experiments.

Similar to Triapine, **H**_**2**_**L**^**6**^ failed to induce ROS, suggesting that ROS are not involved in the toxicity of the ligands (Figure 6). In contrast, treatment with **6** resulted in significant ROS induction in both cell lines in accord with its slightly lower reduction potential (by 70 mV) and faster reduction kinetics as compared to **3**–**5**.

#### 2.10.3 mR2 RNR Inhibition by the Proligands and Their Copper(II) Complexes

**Figure 7:**
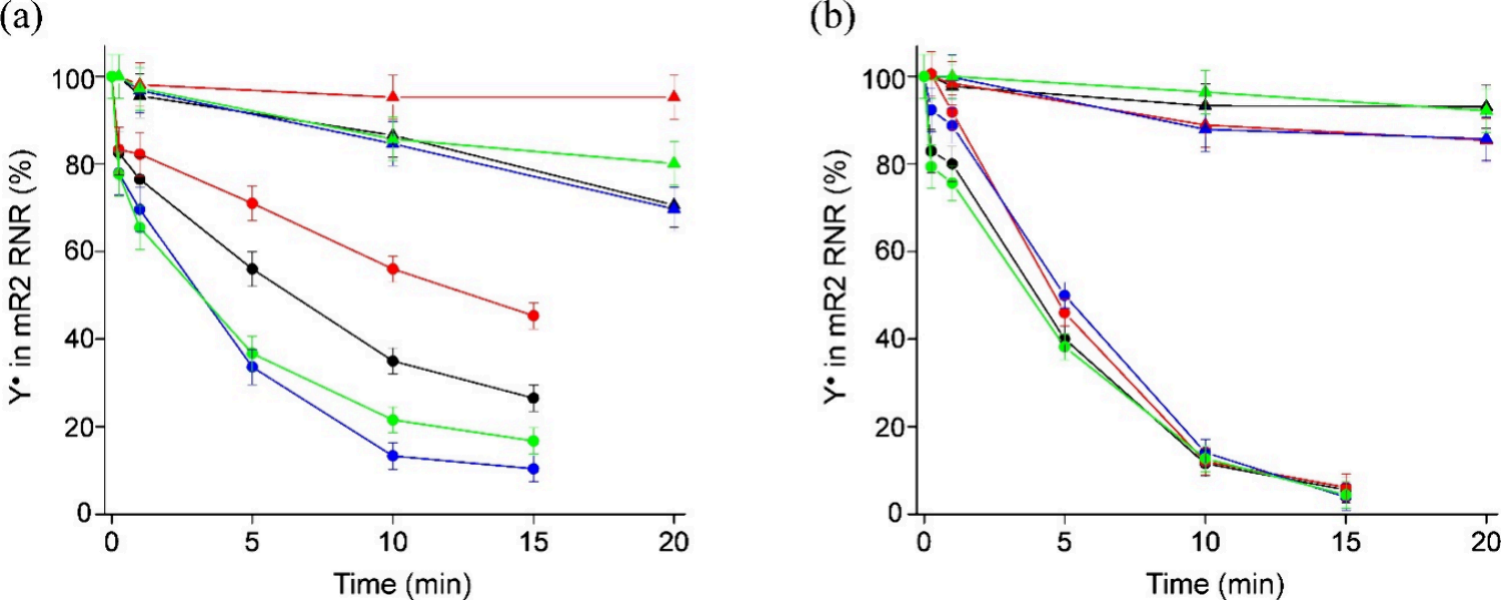
Tyrosyl radical reduction kinetics of mR2 RNR (20 μM) in the absence (triangles) and in the presence (circles) of an external reductant (2 mM DTT) at the 1:1 protein-to-drug ratio, measured by EPR spectroscopy at 30 K, for (a) **H**_**2**_**L**^**3**^ (black), **H**_**2**_**L**^**4**^ (red), **H**_**2**_**L**^**5**^ (blue), and **H**_**2**_**L**^**6**^ (green) and for (b) **3** (black), **4** (red), **5** (blue), and **6** (green).

Since α-*N*-heterocyclic TSCs are very potent R2 RNR inhibitors, the time-dependent Y^•^ reduction of the mR2 RNR protein was measured. The effect of equimolar concentrations of the proligands **H**_**2**_**L**^**3**^–**H**_**2**_**L**^**6**^ and their copper(II) complexes **3**–**6**, in the absence and presence of an external reductant DTT, is shown in Figure 7.

The proligands **H**_**2**_**L**^**4**^, **H**_**2**_**L**^**3**^, **H**_**2**_**L^6^**, and **H**_**2**_**L**^**5**^ reduced ca. 55, 70, 80, and 90%, respectively, of Y^•^ in the mR2 protein after 15 min in the presence of DTT. Without DTT, **H**_**2**_**L**^**4**^ had no effect on quenching of Y^•^, while **H**_**2**_**L**^**3**^, **H**_**2**_**L**^**5**^, and **H**_**2**_**L**^**6**^ reduced ca. 20% (Figure 7a). The Y^•^ reduction efficiency of the investigated proligands was increased upon their coordination to copper(II) and in the presence of DTT. Complexes **3**–**6** showed comparable reducing potency, by quenching ca. 90% of Y^•^ in the presence of DTT after 10 min (Figure 7b). It can be concluded that the investigated proligands are not as efficient Y^•^ reductants as Triapine, which is able to reduce 100% of mR2 Y^•^ in 3 min.^76^ Nevertheless, the coordination to Cu(II) results in complexes with strong mR2 RNR inhibition ability.

#### 2.10.4 Complex **6** Induces Significant Changes in the Size and Balance of dNTP Pools in Cells

**Figure 8:**
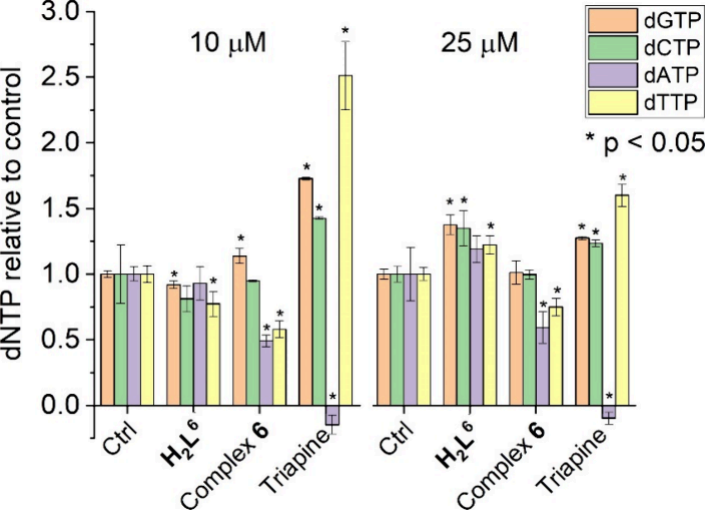
Measurement of dNTP pools in MES-SA cell extracts following a 24 h treatment with proligand **H**_**2**_**L**^**6**^, complex **6**, or Triapine at the indicated concentrations. Three independent experiments were performed; error bars represent standard error; stars indicate significant changes compared to the respective control (Ctrl) at *p* < 0.05.

RNR reduces NDPs to their corresponding deoxy derivatives (dNDPs), which are further converted into dNTPs.^77^ Therefore, we investigated the effect of the most toxic proligand **H_2_L^6^** and its copper(II) complex **6** on cellular dNTP levels, using Triapine as a control. In response to the treatment with proligand **H_2_L^6^**, we observed a small and uniform decrease in all dNTP levels at 10 μM and a small uniform increase at 25 μM (Figure 8). In the case of complex **6**, however, the dNTP pool balance is affected with a marked decrease in the dATP and deoxythymidine triphosphate (dTTP) levels at both concentrations. These results are consistent with studies showing that the knockdown of either the R2 or R1 RNR subunit results in asymmetric changes in the dNTP pools.^78^ Triapine also elicits a dATP pool decrease while increasing the pools of the three other dNTP species. Decreasing the dATP pool to zero can give rise to the observed cytotoxic effect upon treatment with Triapine. Complex **6** seems to affect dNTP pools by a more complex mechanism, which is nevertheless consistent with R2 inhibition.^79^

#### 2.10.5 Interference with Tubulin Polymerization

Intrigued by the submicromolar IC_50_ values of **H**_**2**_**L**^**6**^ and the ability of recently reported TSCs to act as tubulin-targeting agents,^27^ we also tested the effect of proligands **H**_**2**_**L**^**3**^**–H**_**2**_**L**^**6**^ and the copper(II) complexes **3**–**6** on the polymerization of purified tubulin. As a reference for comparison, combretastatin A-4 (CA-4) was used. As shown in Table 6, significant inhibition was only observed with the complexes while all tested TSC ligands showed IC_50_ values >20 μM. The most active was complex **6**, followed by **3** and then by complexes **4** and **5**, indicating that the position of the morpholine unit has a significant effect on the inhibition of tubulin polymerization. The two most active complexes **3** and **6** were further studied for their abilities to inhibit the binding of [^3^H]colchicine to tubulin at two different concentrations (5 and 25 μM), with tubulin and colchicine at 0.5 and 5 μM concentrations, respectively (Table 6).^80,81^ The data obtained show that these two compounds showed different potency in their ability to inhibit the binding of [^3^H]colchicine to tubulin but are 4.5-fold and 17-fold less potent than CA-4 at the 5 μM concentration.

**Table 6:** Inhibition of Tubulin Polymerization and Colchicine Binding by **H**_**2**_**L**^**3**^**–H**_**2**_**L**^**6**^ and **3**–**6**^a^

	inhibition of tubulin	inhibition of colchicine binding		
	IC_50_ ± SD (μM)	% inhibition ± SD		
compound		0.5 μM inhibitor	5 μM inhibitor	25 μM inhibitor
**C-A4**^b^	0.91 ± 0.1	85 ± 2	98 ± 0.5	
**3**	7.0 ± 0.3		22 ± 2	40 ± 3
**4**	15 ± 1			
**5**	15 ± 0.4			
**6**	5.0 ± 0.3		5.8 ± 5	33 ± 2
**H**_**2**_**L**^**3**^–**H**_**2**_**L**^**6**^	>20			

a Each experiment was performed two to three times, and SDs are presented.

b Combretastatin A-4.

Nevertheless, comparison of the IC_50_ values for antiproliferative activity of **6** and **3** in cancer cells MES-SA and MES-SA/Dx5 (0.29 and 15 μM, and 1.4 and 5.5 μM, respectively) and inhibition of pure tubulin (5 and 7 μM, respectively) shows that all are mainly in the low micromolar range. This might indicate that the mode of action of these two compounds, in addition to RNR inhibition, involves inhibition of tubulin assembly. Interestingly, complex formation with Cu(II) resulted in significant enhancement of the ability of proligands to inhibit tubulin by binding to the colchicine site.

#### 2.10.6 Cell Cycle

**Figure 9:**
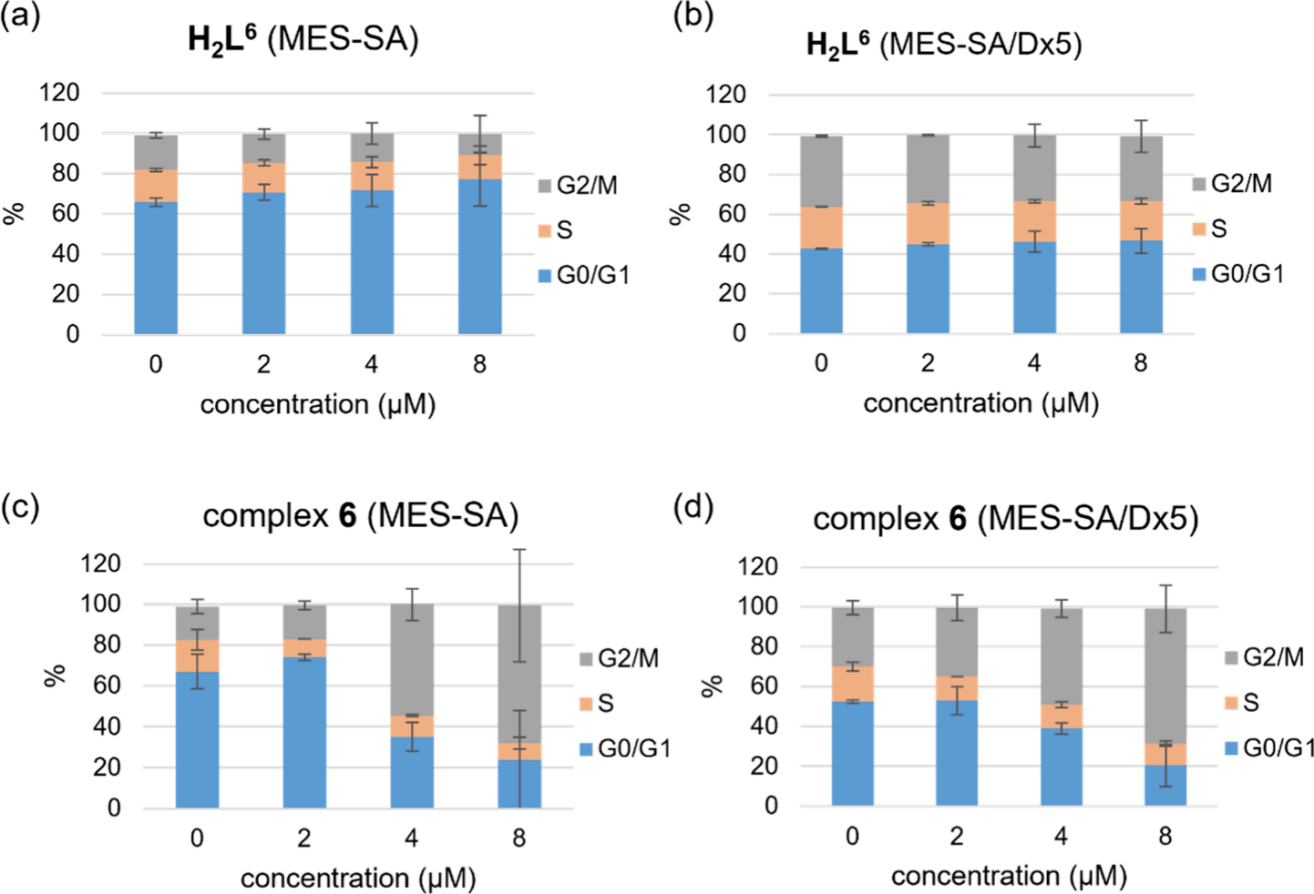
Cell cycle arrest for proligand **H**_**2**_**L**^**6**^ in (a) the MES-SA and in (b) MES-SA/Dx5 cells, as well as for complex **6** in (c) the MES-SA and in (d) MES-SA/Dx5 cancer cell lines.

Cell cycle arrest in S-phase has been observed for Triapine analogues at concentrations ranging from 0.25 to 5.0 μM,^82,83^ in line with a mechanism of action relying on RNR inhibition. Whereas **H**_**2**_**L**^**4**^ arrested the cells in S-phase, treatment with **4** gradually increased the G2/M ratio in a concentration-dependent manner (Supporting Information Figure S18 and Table S4). Similarly, **6** induced G2/M-phase arrest at concentrations ≥4 μM while no effect was observed for **H**_**2**_**L**^**6**^ (Figure 9, Table S5 in the Supporting Information).

Downregulation of R2 and the consequent reduction of dATP levels, as well as tubulin inhibition, are in accordance with cell cycle arrest in the G2/M phase.^79^

#### 2.10.7 Molecular Docking Study of Proligands and Copper(II) Complexes

The binding pocket for Triapine in the mR2 RNR protein is established.^76^ To estimate the possibility of binding of proligands **H**_**2**_**L**^**3**^–**H**_**2**_**L**^**6**^ and their corresponding copper(II) complexes **3**–**6** to mR2 RNR (PDB ID: 1W68), docking studies were conducted using the GOLD software.^84^ Reasonable scores were predicted for the proligands **H**_**2**_**L**^**3**^–**H**_**2**_**L**^**6**^ for all the scoring functions used, suggesting a good binding to R2 RNR, i.e., 53–55 for GS (Gold Score), 55–61 for ChemPLP (Chem Piecewise Linear Potential), 26–29 for CS (ChemScore), and 28–33 for ASP (Astex Statistical Potential) (Table S6, Supporting Information). To predict the binding of **3**–**6**, only the GS scoring function was used because the others are not parametrized for metal complexes. GS parameters were modified for copper(II) complexes since they are not included in GOLD’s database.^85^ The scores produced were comparable (50–52) to those obtained with the proligands. Furthermore, according to mainstream calculated molecular descriptors (Table S7, Supporting Information), all compounds lie in the drug-like space indicating good biocompatibility.^86^

**Figure 10:**
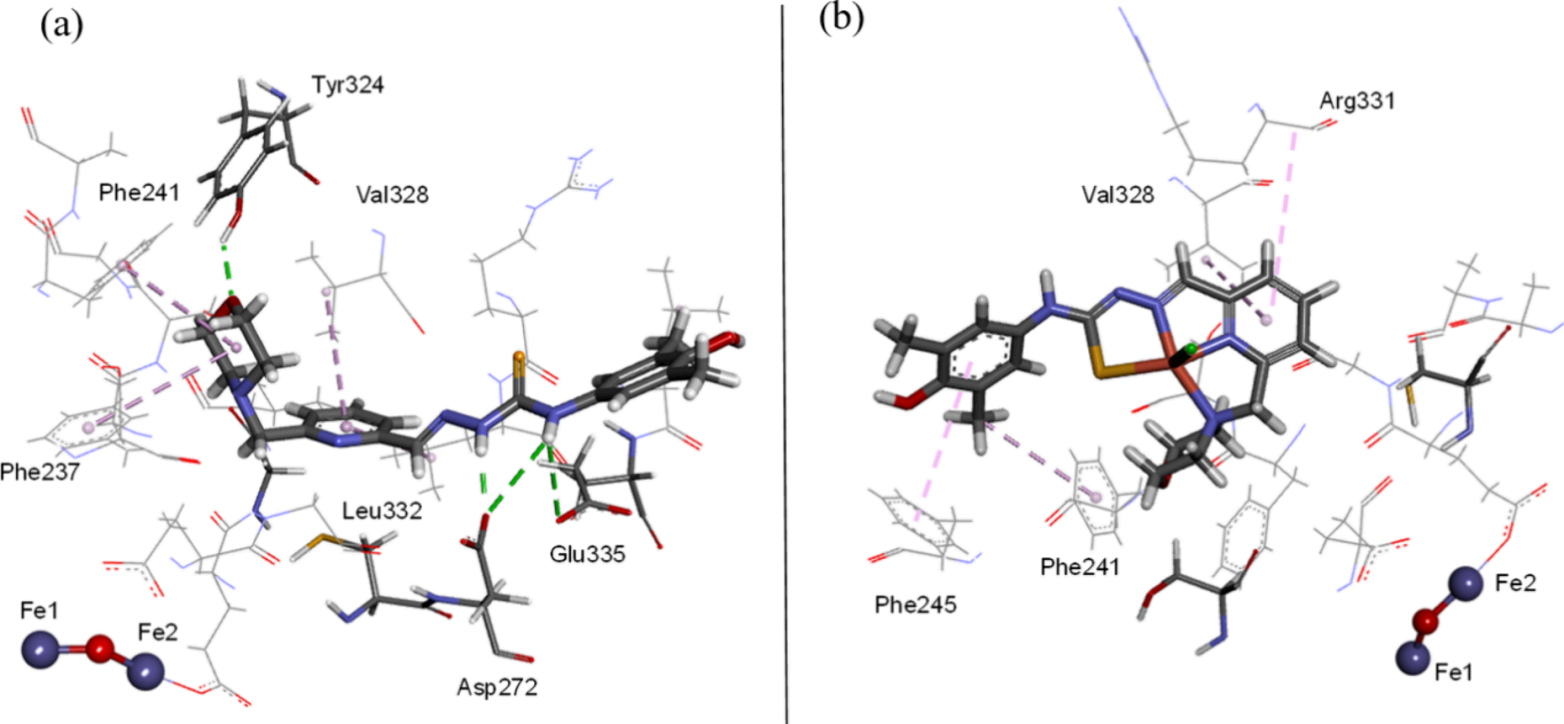
Docked conformation of (a) proligand **H**_**2**_**L**^**6**^ and (b) its copper(II) complex **6** in the binding site of mR2 RNR (PDB ID: 1W68). The hydrogen bond interactions are depicted as green lines, and lipophilic contacts are shown as purple dashed lines.

**Figure 11:**
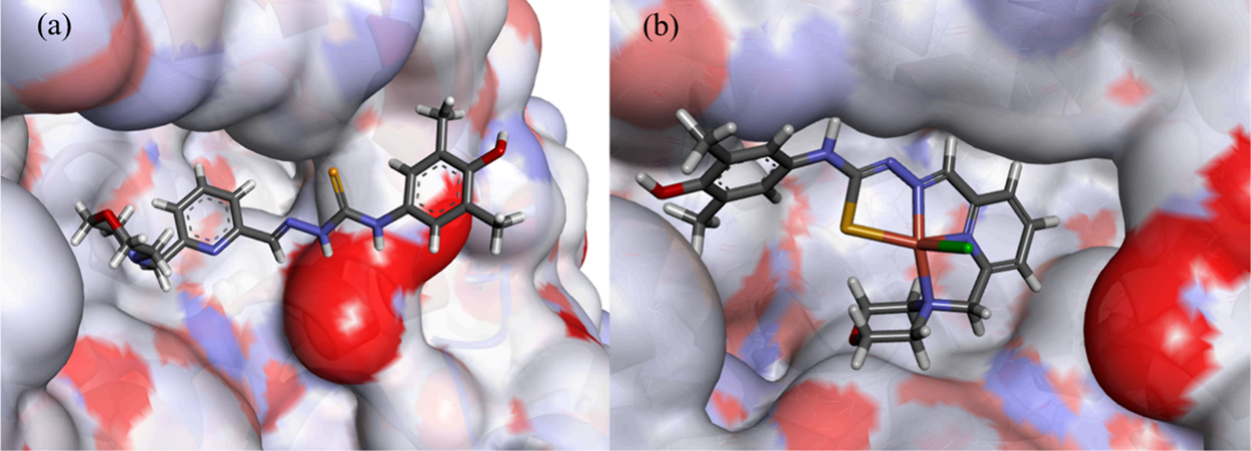
(a) Docked conformation of proligand **H**_**2**_**L**^**6**^ and (b) its copper(II) complex **6** in the binding site of mR2 RNR. The surface is rendered; blue and red depict positive and negative charges, respectively.

Docking of **3**–**6** resulted in a predicted pose across the binding pocket, where the lipophilic core of the complexes was embedded deep in the pocket of the R2 subunit across several lipophilic contacts. In general, similar docking results were observed for all the active proligands and their complexes (Table S7), suggesting a plausible binding to the mR2 RNR protein. The docked conformation of the lead proligand **H**_**2**_**L**^**6**^ and its complex **6** into the binding site is shown in Figures 10 and 11. The complex **6** displayed lipophilic contacts with the F_237_, F_241_, F_245_, R_331_, and V_328_ amino acid residues, in near proximity to the Fe_2_O cofactor.

The essential step required for RNR activity is the transfer of the electron from Y^•^ in the active cofactor of the R2 subunit to the cysteine (C) in the active site of the R1 subunit, generating a putative thiyl radical (in the case of mR2 RNR, from Y^•^_137_ to C_439_).^13,87^ This process occurs *via* proton-coupled electron transfer (PCET), an intersubunit pathway consisting of a chain of hydrogen bonded amino acid residues.^13,14^ It is worth highlighting that the docking calculations indicated that the proligand **H**_**2**_**L**^**3**^ and its copper(II) complex **3** are in lipophilic contact with arginine R_265_. Recent findings suggest that amino acid residue R_265_ (Table S6, Supporting Information), which has been suggested to participate in the PCET pathway in mR2 RNR, acts as a proton mediator during catalysis.^87^

In addition, Cu(II) complexes **3**–**6** and their proligands **H**_**2**_**L**^**3**^–**H**_**2**_**L**^**6**^ were docked into the colchicine site of tubulin (PDB ID: 1SA0, resolution 3.58 Å).^88^ The robustness of the model was previously established, and the docking protocol is available.^28^ The predicted binding scores are given in Table S8; all ligands and complexes show good scores indicating reasonable binding. When the cocrystallized ligand *N*-deacetyl-*N-*(2-mercaptoacetyl)-colchicine (DAMA-colchicine) was redocked and scored, it yielded a somewhat lower value than for our ligands and was in a similar range as for the complexes.

**Figure 12:**
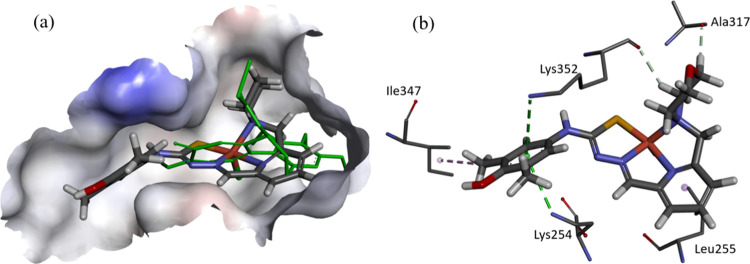
Docked pose of **6** using GS in the tubulin colchicine site: (a) The cocrystallized ligand DAMA-colchicine is shown in green line format, but its hydrogens are not shown for clarity. The predicted configuration is shown in stick format. The protein surface is rendered; blue depicts regions with a partial positive charge on the surface; red depicts regions with a partial negative charge; and gray shows neutral areas. (b) The predicted interactions are shown as dashed lines with the corresponding amino acid residues. The weak hydrogen bonds are colored gray, lipophilic contacts are purple, and π – NH are green.

The modeling of complex **6** showed a good fit within the pocket and an extensive overlap with the DAMA-colchicine cocrystallized ligand; the copper-chlorido vector is pointing into the pocket, and the metal center is not in the proximity of any chelating residues (Figure 12a). Furthermore, the morpholine moiety is placed deep within the pocket and the phenyl ring is sitting in a lipophilic groove. Only weak interactions between the complex and tubulin are predicted rather than classical hydrogen bonding or metal chelating (Figure 12b). The other complexes are predicted to bind in different configurations than **6**. The ligands adopt different poses within the binding pocket, albeit they have a good overlap with the DAMA-colchicine-cocrystallized ligand and complex **6**.

## 3 Conclusions

This work led to four isomeric TSC hybrids **H**_**2**_**L**^**3**^–**H**_**2**_**L**^**6**^, each bearing a redox active *para*-amino-dimethylphenol unit, and with a morpholine moiety at the four available positions of the pyridine ring and to a series of four Cu(II) complexes (**3**–**6**) of these TSC hybrids. SC-XRD revealed that TSCs **H**_**2**_**L**^**3**^–**H**_**2**_**L**^**5**^ acted in the Cu(II) complexes **3**–**5** as monoanionic tridentate NNS ligands, while **H**_**2**_**L**^**6**^ in **6** acted as a monoanionic tetradentate NNNS ligand. Solution speciation studies showed that the proligands were present at physiological pH in their neutral forms in 30% (v/v) DMSO, while complexes **3**–**6** were monocations [Cu(HL)]^+^ (H_2_L = **H**_**2**_**L**^**3**^–**H**_**2**_**L**^**6**^). It is of particular note that the position of attachment of the morpholine moiety at the pyridine ring of TSC is important. The dimeric centrosymmetric associates were found in the crystals of **3** and **4**, even though they do not remain intact in solution and dissociate in square-planar monomeric species. All four ligands formed stable Cu(II) complexes, in agreement with the conditional stability constants. Increase of the denticity of the ligand to four in **6** did not afford higher thermodynamic stability as compared to **3**–**5**, in which the respective ligands acted as tridentate. However, **6** was found to be reduced more easily by 70 mV. In accordance with this finding, **6** was reduced by GSH somewhat faster than were **3**–**5**. The compounds showed antiproliferative activity in the MES-SA and its MDR counterpart (MES-SA/Dx5) cell lines with IC_50_ values varying from 0.25 to 31.0 μM and from 1.4 to 13.1 μM, respectively. The MDR cells were found to be more sensitive to Cu(II) complexes **3**–**5**, but the increased sensitivity was not dependent on the function of Pgp, as indicated by the Tariquidar controls. The hit compounds **H**_**2**_**L**^**6**^ in **6** proved to be more cytotoxic than Triapine in both cell lines, and **6** was threefold more cytotoxic in MDR cells than **H**_**2**_**L**^**6**^. The proligands reduced from 55 to 90% the tyrosyl radical in the mR2 protein in the presence of DTT, while complexes **3**–**6** all quenched Y^•^ by 90%. Moreover, **6** was found to affect dNTP pools at 10 μM, also consistent with R2 inhibition. Studies to evaluate the ability of the proligands and Cu(II) complexes to inhibit tubulin polymerization showed good activity of **3**–**6**, with the lowest IC_50_ value of 5.0 μM for **6**, which also inhibited colchicine binding to tubulin, while **H**_**2**_**L**^**3**^–**H**_**2**_**L**^**6**^ were markedly less active as inhibitors of tubulin assembly (IC_50_ > 20 μM). Comparison of the IC_50_ values of antiproliferative activity of **3**–**6** with their ability to reduce the tyrosyl radical of the mR2 protein and to affect dNTP pools, as well as their IC_50_ values for inhibition of tubulin assembly, leads to the conclusion that both R2 RNR and tubulin might be targets of these Cu(II) complexes and cause their antiproliferative activity. The involvement of both targets was consistent with molecular docking calculations. The complexes **3**–**6** are the first reported transition metal complexes of TSCs that bind to tubulin in the colchicine site. Finally, the potential of developing copper(II) complexes of TSCs as single drugs with dual action as R2 RNR and tubulin polymerization inhibitors has been demonstrated. This would fit with current practice to combine MTAs with other anticancer drugs to enhance therapeutic results.^89,90^ A single agent with such dual action should induce cell cycle arrest and might lead to other unexpected advantages.^91^ We have yet to determine whether the presence of the redox active amino-dimethylphenol moiety is playing a role in quenching the tyrosyl radical in the R2 protein by its reduction. The work on the synthesis of Cu(II) complexes with 2e oxidized TSCs at the redox unit are ongoing in our laboratory. In addition, **3** and **6** are suitable platforms for further structural optimization, guided by molecular modeling calculations, followed by synthesis and assay of their antitubulin activity to obtain better lead drug candidates.

## 4 Experimental Section

### 4.1 Chemicals

The detailed seven-step synthesis of 3-(morpholinomethyl)-2-formylpyridine is described in Section 4.2 below. 4-, 5-, and 6-(Morpholinomethyl)-2-formylpyridine, as well as 4-(4-hydroxy-3,5-dimethylphenyl)thiosemicarbazide, were synthesized as described previously.^46,52,45,51^

All solvents and CuCl_2_·2H_2_O were purchased from commercial suppliers and used as received. KCl, KOH, HCl, and DMSO were obtained from Reanal (Hungary). KH_2_PO_4_, Na_2_HPO_4_, GSH, ascorbic acid, 2-(*N*-morpholino)ethanesulfonic acid (MES), and 4-(2-hydroxyethyl)-1-piperazinethanesulfonic acid (HEPES) were purchased from Sigma-Aldrich and used without further purification. Copper(II) stock solution was prepared by the dissolution of CuCl_2_ in water, and its concentration was determined by complexometry with ethylenediaminetetraacetic acid (EDTA).

### 4.2 Synthesis of 3-(Morpholinomethyl)-2-formylpyridine (F in Scheme 1)

#### 4.2.1 Compound **A**

A white suspension of 2,3-pyridinedicarboxylic anhydride (10.0 g, 67.0 mmol) was refluxed in ^*i*^PrOH (21 mL) for 20 h. The solvent was removed under reduced pressure to give an ocher-yellow residue, which was recrystallized from ethyl acetate (300 mL). The crystalline product was isolated by filtration, washed with ethyl acetate (5–10 mL), and dried *in vacuo* for 3 h. Yield: 7.3 g, 52%. ^1^H NMR (500 MHz, DMSO-*d*_6_), δ,ppm: 13.86 (s, 1H, COOH); 8.75 (dd, 1H, Ar–H); 8.27 (dd, 1H, Ar–H); 7.65 (dd, 1H, Ar–H); 5.14 (heptet, 1H, C–H); 1.30 (d, 6H, 2 × CH_3_). ESI-MS (negative mode): *m*/*z* = 208 [M–H^+^]^−^.

#### 4.2.2 Compound **C**

To a suspension of compound **A** (7.1 g, 33.8 mmol, 1.0 equiv) in CH_2_Cl_2_ (36 mL) were added thionyl chloride (3.6 mL, 49.1 mmol, 1.5 equiv) and a catalytic amount of DMF (0.8 mL, 10.2 mmol, 0.3 equiv). The reaction mixture was stirred under reflux for 3 h. The solvent was removed under reduced pressure to give an orange oil. THF (40 mL) was added, and the reaction mixture was concentrated under reduced pressure. This process was repeated three times to remove unreacted thionyl chloride. The orange oily residue of **B** was diluted with THF (30 mL) and cooled to 0 °C, and NaBH_4_ (1.7 g, 44.9 mmol) was added. The mixture was stirred at 0 °C for 2 h, and the reaction was quenched by addition of ice directly to the orange suspension until foaming was complete. The product was extracted with CH_2_Cl_2_ (2 × 400 mL) and dried over anhydrous Na_2_SO_4_. The solvent was evaporated under reduced pressure and dried *in vacuo* to give an orange oil, which crystallized after 3 days. Yield: 5.7 g, 87%. ^1^H NMR (500 MHz, DMSO-*d*_6_), δ, ppm: 8.52 (dd, 1H, Ar–H); 8.05 (dd, 1H, Ar–H); 7.58 (dd, 1H, Ar–H); 5.44 (m, 1H, OH); 5.13 (m, 1H, C–H); 1.30 (d, 6H, 2 × CH_3_). ESI-MS (positive mode): *m*/*z* 196 [M + H]^+^, 218 [M + Na]^+^.

#### 4.2.3 Compound **D**

To a solution of species **C** (5.7 g, 29.0 mmol, 1.0 equiv) in CH_2_Cl_2_ (50 mL), triethylamine (6.1 mL, 43.5 mmol, 1.5 equiv) was added. The reaction mixture was cooled to 0 °C, and MeSO_2_Cl (2.5 mL, 31.9 mmol, 1.1 equiv) was added dropwise and stirred for 1 h. The reaction mixture was refluxed overnight. Acetonitrile (50 mL) was added, and the reaction mixture was refluxed for ca. 1.5 h until CH_2_Cl_2_ (ca. 35 mL) was removed to give an intermediate. Morpholine (5.2 mL, 58.0 mmol, 2.0 equiv) was added, and the reaction mixture was refluxed for 4 h. The dark-red solution had a strong smell of triethylamine and was concentrated under reduced pressure and poured into H_2_O (50 mL). The product was extracted with ethyl acetate (2 × 100 mL). The solvent was evaporated under reduced pressure, and the product **D** was dried *in vacuo* for 4 h. Yield: 5.4 g, 70%. ^1^H NMR (500 MHz, DMSO-*d*_6_) δ 8.50 (dd, 1H, Ar–H); 7.85 (dd, 1H, Ar–H); 7.48 (dd, 1H, Ar–H); 5.14 (m, 1H, C–H); 3.60 (s, 2H, CH_2_); 3.51 (dd, 4H, morph); 2.29 (m, 4H, morph); 1.33 (m, 6H, 2 × CH_3_); signals at 3.56 and 2.40 ppm from morpholine are also observed. ESI-MS (positive mode): *m*/*z* 265 [M + H]^+^, 287 [M + Na]^+^.

#### 4.2.4 Compound **E**

A solution of species **D** (5.4 g, 20.3 mmol, 1.0 equiv) in EtOH (40 mL) was cooled in an ice bath and stirred for 10 min. NaBH_4_ (1.5 g, 40.6 mmol, 2.0 equiv) was added. The reaction mixture turned from brown to orange and was stirred at 0 °C for 1.5 h. Ethyl acetate (80 mL) was added, and the reaction mixture was stirred for 15 min. Addition of H_2_O (140 mL) caused the formation of a white precipitate. The product was extracted with ethyl acetate (4 × 100 mL). Yellow-orange organic phases were combined and concentrated under reduced pressure. The orange oil was dried *in vacuo* overnight. Yield: 2.5 g, 59%. ^1^H NMR (500 MHz, DMSO-*d*_6_), δ, ppm: 8.42 (dd, 1H, Ar–H); 7.72 (dd, 1H, Ar–H); 7.29 (dd, 1H, Ar–H); 5.46 (m, 1H, OH); 4.46 (s, 2H, CH_2_); 3.58 (dd, 4H, morph); 3.54 (s, 2H, CH_2_); 2.38 (m, 4H, morph). ESI-MS (positive mode): *m*/*z* 209 [M + H]^+^, 231 [M + Na]^+^.

#### 4.2.5 Compound **F**

To a solution of species **E** (4.1 g, 20.0 mmol, 1.0 equiv) in dioxane (150 mL), SeO_2_ (2.4 g, 22.0 mmol, 1.1 equiv) was added, and the reaction mixture was refluxed for 4 h. A clear orange solution and a black precipitate on the walls of the flask were formed. The reaction mixture was stirred at room temperature overnight, filtered through Celite, and washed with dioxane (30 mL). The orange solution was concentrated under reduced pressure to give an orange oil with white precipitate. This residue was extracted with Et_2_O (2 × 50 mL), the organic phase filtered and concentrated under reduced pressure to give a yellow-orange oil. Yield: 2.4 g, 59%. ^1^H NMR (500 MHz, DMSO-*d*_6_), δ, ppm: 10.13 (s, 1H, HC = O); 8.71 (dd, 1H, Ar–H); 8.06 (dd, 1H, Ar–H); 7.64 (dd, 1H, Ar–H); 3.88 (s, 2H, CH_2_); 3.56 (m, 4H, morph); 2.40 (m, 4H, morph). ESI-MS (positive mode): *m*/*z* 207 [M + H]^+^, 229 [M + Na]^+^.

### 4.3 The Syntheses of the Proligands (**H**_**2**_**L**^**3**^–**H**_**2**_**L**^**6**^)

#### General Method

To a hot yellow solution of the corresponding aldehyde (0.5 mmol) in EtOH (10 mL), a solution of 4-(4-hydroxy-3,5-dimethylphenyl)thiosemicarbazide (0.55 mmol) in EtOH (40 mL) was added. The clear yellow reaction mixture was refluxed for 4 h and cooled to room temperature, concentrated under reduced pressure (until *ca.* 5 mL), and stored at 4 °C overnight. The pale-yellow crystalline product was removed by filtration, washed with EtOH (5 mL) and Et_2_O (5 mL), and dried in air for 20 min.

##### H_2_L^3^·1.1H_2_O

Yield: 139 mg, 66%. Mp 212–213 °C. Elem. anal. calcd for C_20_H_25_N_5_O_2_S·1.1H_2_O (*M*_r_ = 419.33), %: C, 57.28; H, 6.54; N, 16.70; S, 7.65. Found, %: C, 57.08; H, 6.46; N, 16.32; S, 7.57. ^1^H NMR (600 MHz, DMSO-*d*_6_) for *E*-isomer, δ,ppm:11.39 (s, 1H, N^3′^H); 9.45 (s, 1H, N^4’^H); 8.55 (d, *J* = 7.6 Hz, 1H, C^6^H); 8.47 (s, 1H, HC^1^=N); 8.22 (s, 1H, OH); 7.88 (d, *J* = 7.6 Hz, 1H, C^4^H); 7.38 (dd, *J* = 7.6, 4.7 Hz). 1H, C^5^H); 7.10 (s, 2H, C^14^H and C^18^H); 3.78 (s, 2H, C^7^H_2_); 3.61–3.49 (m, 4H, C^9^H_2_ and C^10^H_2_); 2.46–2.32 (m, 4H, C^8^H_2_ and C^11^H_2_); 2.16 (s, 6H, C^19^H_3_ and C^20^H_3_). ^13^C NMR (151 MHz, DMSO-*d*_6_) for *E*-isomer, δ, ppm: 176.14 (C^12^); 150.96 (C^16^); 150.57 (C^2^); 147.99 (C^6^); 142.69 (C^1^); 138.00 (C^4^); 133.40 (C^3^); 129.91 (C^13^); 125.00 (C^14^ and C^18^); 124.02 (C^15^ and C^17^); 123.43 (C^5^); 66.16 (C^9^ and C^10^); 58.86 (C^7^); 53.20 (C^8^ and C^11^); 16.65 (C^19^ and C^20^). ^1^H NMR (600 MHz, DMSO- *d*_6_) for *Z*-isomer, δ, ppm: 14.50 (s, 1H, N^3′^H); 10.18 (s, 1H, N^4’^H); 8.69 (s, 1H, C^6^H); 8.21 (s, 1H, OH); 7.92 (d, *J* = 7.6 Hz, 1H, C^4^H); 7.80 (s, 1H, C^1^H); 7.50 (dd, *J* = 5.8, 5.4 Hz, 1H, C^5^H); 7.09 (s, 2H, C^14^H and C^18^H); 3.71 (s, 2H, C^7^H_2_); 3.61–3.49 (m, 4H, C^9^H_2_ and C^10^H_2_); 2.46–2.32 (m, 4H, C^8^H_2_ and C^11^H_2_); 2.16 (s, 6H, C^19^H_3_ and C^20^H_3_). ^13^C NMR (151 MHz, DMSO-*d*_6_) for *Z*-isomer, δ,ppm: 176.51 (C^12^); 151.03 (C^16^); 150.63 (C^2^); 146.93 (C^6^); 140.26 (C^4^); 134.09 (C^3^); 130.44 (C^1^); 130.07 (C^13^); 125.33 (C^14^ and C^18^); 124.19 (C^5^); 123.85 (C^15^ and C^17^); 66.13 (C^9^ and C^10^); 58.88 (C^7^); 52.93 (C^8^ and C^11^); 16.66 (C^19^ and C^20^). ESI-MS (positive mode): *m*/*z* 400 [H_2_L^3^+H]^+^, 422 [H_2_L^3^+Na]^+^. ESI-MS (negative mode): *m*/*z* 398 [HL^3^]^−^. IR (ATR, selected bands υ̃_max_): 3225, 3189, 2939, 2857, 2824, 2778, 1743, 1686, 1603, 1537, 1487, 1442, 1304, 1251, 1217, 1191, 1115, 1085, 1003, 928, 900, 857, 757, 688 cm^–1^. X-ray diffraction quality single crystals were obtained from ethanolic solution at 4 °C.

##### H_2_L^4^·0.25H_2_O

Yield: 175 mg, 87%. Mp 213-214 °C. calcd for C_20_H_25_N_5_O_2_S·0.25H_2_O (*M*_r_ = 404.01), %: (%) C, 59.47; H, 6.36; N, 17.33; S, 7.94. Found, %: C, 59.54; H, 6.37; N, 16.92; S, 7.88. ^1^H NMR (500 MHz, DMSO-*d*_6_) for *E*-isomer, δ, ppm: 11.87 (s, 1H, N^3′^H); 9.97 (s, 1H, N^4’^H); 8.51 (d, *J* = 5.0 Hz, 1H, C^6^H); 8.28 (s, 1H, C^3^H); 8.25 (s, 1H, OH); 8.16 (s, 1H, C^1^H); 7.36 (dd, *J* = 5.1, 1.4 Hz, 1H, C^5^H); 6.99 (s, 2H, C^14^H and C^18^H); 3.58 (m, C^9^H_2_ and C^10^H_2_); 3.52 (s, 2H, C^7^H_2_); 2.36 (s, 4H, C^8^H_2_ and C^11^H_2_); 2.17 (s, 6H, C^19^H_3_ and C^20^H_3_). ^13^C NMR (126 MHz, DMSO-*d*_6_) for *E*-isomer, δ, ppm: 177.18 (C^12^); 153.75 (C^2^); 151.73 (C^16^); 149.75 (C^6^); 148.06 (C^4^); 143.13 (C^1^); 130.63 (C^13^); 127.12 (C^14^ and C^18^); 124.37 (C^15^ and C^17^); 124.58 (C^5^); 121.02 (C^3^); 66.57 (C^9^ and C^10^); 61.70 (C^7^); 53.72 (C^8^ and C^11^); 17.10 (C^19^ and C^20^). ^1^H NMR (500 MHz, DMSO-*d*_6_) for *Z*-isomer, δ, ppm: 14.28 (s, 1H, N^3′^H); 10.18 (s, 1H, N^4’^H); 8.71 (s, 1H, C^6^H); 8.23 (s, 1H, OH); 7.77 (s, 1H, C^3^H); 7.49 (s, 2H, C^14^H and C^18^H); 7.07 (s, 1H, C^5^H); 3.61 (m, 4H, C^9^H_2_ and C^10^H_2_; overlapped by the signal of *E*-isomer); n.d (C^7^H_2_); 2.40 (s, 4H, C^8^H_2_ and C^11^H_2_); 2.15 (s, 6H, C^19^H_3_ and C^20^H_3_). ^13^C NMR (126 MHz, DMSO-*d*_6_) for *Z*-isomer, δ, ppm: n.d (C^12^); 153.75 (C^2^); n.d. (C^16^); 148.85 (C^6^); n.d. (C^4^); n.d. (C^1^); n.d. (C^13^); 126.74 (C^3^); 125.93 (C^5^); 124.97 (C^14^ and C^18^); n.d (C^15^ and C^17^); 61.25 (C^7^); 56.49 (C^9^ and C^10^); 53.66 (C^8^ and C^11^); 17.16 (C^19^ and C^20^). ESI-MS (positive mode): *m*/*z* 400 [H_2_L^4^+H]^+^, 422 [H_2_L^4^+Na]^+^. ESI-MS (negative mode): *m*/*z* 398 [HL^4^]^−^. IR (ATR, selected bands υ̃_max_): 3285, 3216, 2949, 2911, 2861, 2813, 1592, 1519, 1453, 1422, 1388, 1344, 1301, 1193, 1148, 1103, 1072, 1032, 986, 954, 917, 859, 793, 751, 725 cm^–1^. X-ray diffraction quality single crystals of **[H**_**4**_**L**^**4**^**]Cl**_**2**_ were obtained from ethanolic solution of **H**_**2**_**L**^**4**^ upon addition of conc. HCl (2 equiv) at 4 °C after 7 days.

##### H_2_L^5^·EtOH·0.4H_2_O

Yield: 202 mg, 89%. Mp 174–175 °C. calcd for C_20_H_25_N_5_O_2_S·EtOH·0.4H_2_O (*M*_r_ = 452.79), %: C, 58.36; H, 7.08; N, 15.47; S, 7.08. Found, %: C, 58.66; H, 6.90; N, 15.59; S, 7.25. ^1^H NMR (600 MHz, DMSO-*d*_6_) for *E*-isomer, δ, ppm: 11.84 (s, 1H, N^3′^H); 9.97 (s, 1H, N^4’^H); 8.49 (d, *J* = 0.9 Hz, 1H, C^6^H); 8.39 (d, *J* = 8.1 Hz, 1H, C^3^H); 8.21 (s, 1H, OH); 8.15 (s, 1H, C^1^H); 7.74 (dd, *J* = 9.3, 1.1 Hz, 1H, C^4^H); 7.01 (s, 2H, C^14^H and C^18^H); 3.62–3.57 (m, 4H, C^9^H_2_ and C^10^H_2_); 3.51 (s, 2H, C^7^H_2_); 2.36 (s, 4H, C^8^H_2_ and C^11^H_2_); 2.17 (s, 6H, C^19^H_3_ and C^20^H_3_). ^13^C NMR (151 MHz, DMSO-*d*_6_) for *E*-isomer, δ, ppm: 176.47 (C^12^); 152.28 (C^2^); 151.07 (C^16^); 149.68 (C^6^); 142.40 (C^1^); 136.96 (C^4^); 133.79 (C^5^); 130.16 (C^13^); 126.24 (C^14^ and C^18^); 123.81 (C^15^ and C^17^); 120.08 (C^3^); 66.12 (C^9^ and C^10^); 59.30 (C^7^); 53.04 (C^8^ and C^11^); 16.61 (C^19^ and C^20^). ESI-MS (positive mode): *m*/*z* 400 [H_2_L^5^+H]^+^, 422 [H_2_L^5^+Na]^+^. ESI-MS (negative mode): *m*/*z* 398 [HL^5^]^−^. IR (ATR, selected bands υ̃_max_): 3415, 3165, 2968, 2915, 2851, 2819, 1742, 1694, 1533, 1484, 1349, 1177, 1113, 1089, 1048, 1009, 927, 869, 759, 727, 681 cm^–1^. Crystals suitable for X-ray diffraction were obtained from ethanol at 4 °C.

##### H_2_L^6^·0.75H_2_O

Yield: 120 mg, 58%. Mp 145–146 °C. calcd for C_20_H_25_N_5_O_2_S·0.75H_2_O (*M*_r_ = 413.02), %: C, 58.16; H, 6.47; N, 16.96; S, 7.76. Found, %: C, 58.36; H, 6.07; N, 16.90; S, 7.84. ^1^H NMR (500 MHz, DMSO-*d*_6_) for *E*-isomer, δ, ppm: 11.88 (s, 1H, N^3′^H); 10.00 (s, 1H, N^4’^H); 8.33 (d, *J* = 7.9 Hz, 1H, C^3^H); 8.23 (s, 1H, OH); 8.11 (s, 1H, C^1^H); 7.80 (t, *J* = 7.7 Hz, 1H, C^4^H); 7.42 (d, *J* = 7.5 Hz, 1H, C^5^H); 7.01 (s, 2H, C^14^H and C^18^H); 3.59 (s, 6H, C^7^H_2_, C^9^H_2_ and C^10^H_2_); 2.41 (s, 4H, C^8^H_2_ and C^11^H_2_); 2.17 (s, 6H, C^19^H_3_ and C^20^H_3_). ^13^C NMR (126 MHz, DMSO) for *E*-isomer, δ, ppm: 176.96 (C^12^); 158.33 (C^6^); 153.21 (C^2^); 151.58 (C^16^); 142.99 (C^1^); 137.32 (C^4^); 130.63 (C^13^); 126.79 (C^14^ and C^18^); 124.29 (C^15^ and C^17^); 123.61 (C^5^); 119.39 (C^3^); 66.66 (C^9^ and C^10^); 64.39 (C^7^); 53.80 (C^8^ and C^11^); 17.12 (C^19^ and C^20^). ^1^H NMR (500 MHz, DMSO-*d*_6_) for *Z*-isomer, δ, ppm: 14.51 (s, 1H, N^3′^H); 10.16 (s, 1H, N^4’^H); 8.05 (t, *J* = 7.9 Hz, 1H, C^4^H); 7.69 (d, *J* = 7.8 Hz, 1H, C^3^H or C^5^H); 7.59 (d, *J* = 7.8 Hz, 1H, C^3^H or C^5^H); 7.45 (s, 1H, not attributed); 7.06 (s, 2H, C^14^H and C^18^H); 3.72 (s, 4H, C^8^H_2_ and C^11^H_2_ or C^9^H_2_ and C^10^H_2_); 3.45 (s, 2H, C^7^H_2_); 2.15 (s, 6H, C^19^H_3_ and C^20^H_3_), some of the signals are overlapped by the signals of *E*-isomer. ^13^C NMR (126 MHz, DMSO) for *Z*-isomer, δ, ppm: 139.24 (not attributed); 125.95 (not attributed); 125.40 (not attributed); 64.09 (C^7^); 53.67 (C^8^ and C^11^); 17.17 (C^19^ and C^20^). ESI-MS (positive mode): *m*/*z* 400 [H_2_L^6^+H]^+^, 422 [H_2_L^6^+Na]^+^. ESI-MS (negative mode): *m*/*z* 398 [HL^6^]^−^. IR (ATR, selected bands υ̃_max_): 3197, 3122, 3059, 2952, 2864, 2811, 2765, 1543, 1487, 1452, 1313, 1210, 1159, 1114, 1089, 1029, 1001, 961, 919, 863, 823, 786, 751 cm^–1^. X-ray diffraction quality single crystals of **H**_**2**_**L**^**6**^ were obtained from ethanol at 4 °C.

### 4.4 Synthesis of Copper(II) Complexes (**3**–**6**)

#### General Method

To a hot yellow solution of the appropriate TSC (0.2 mmol) in degassed MeOH (30 mL), Et_3_N (0.2 mmol) was added, and, after 10–15 min, a solution of CuCl_2_·2H_2_O (0.2 mmol) in MeOH (2 mL) was added dropwise. The brown reaction mixture was refluxed under Ar for *ca.* 10 min and at room temperature overnight. The precipitate was removed by filtration, washed with MeOH (2 × 5 mL) and Et_2_O (2 × 5 mL), and dried in air for 30 min and *in vacuo* for 4 h.

##### [Cu(HL^3^)Cl]·H_2_O (**3**)

Yield: 71 mg, 69%. Elem. anal. calcd for C_20_H_24_ClCu N_5_O_2_S·H_2_O (*M*_r_ = 515.52), %: C, 46.60; H, 5.08; N, 13.59; S, 6.22. Found, %: C, 46.71; H, 4.66; N, 13.35; S, 6.28. ESI-MS in positive mode (MeCN*/*MeOH + 1% H_2_O): *m*/*z* 461 [Cu(HL^3^)]^+^, 501 [Cu(HL^3^)MeCN]^+^. ESI-MS in negative mode (MeCN*/*MeOH + 1% H_2_O): *m*/*z* 495 [Cu(L^3^)Cl]^−^. UV–vis, λ_max_ [nm] (ε [M^–1^ cm^–1^]): 262 sh, 280 (10,788), 422 (11,050), ∼615 sh. IR (ATR, selected bands υ̃_max_): 3270, 2953, 2910, 2871, 2804, 1754, 1696, 1599, 1536, 1458, 1426, 1352, 1301, 1208, 1140, 1104, 1003, 902, 859, 722, 632 cm^–1^. Single crystals suitable for X-ray diffraction measurement were obtained by vapor diffusion of Et_2_O into a solution of the complex in DMF.

##### [Cu(HL^4^)Cl]·1.5H_2_O (**4**)

Yield: 76 mg, 72%. Elem. anal. calcd for C_20_H_24_ClCuN_5_O_2_S·1.5H_2_O (*M*_r_ = 524.52), %: C, 45.80; H, 5.19; N, 13.35; S, 6.11. Found, %: C, 45.68; H, 4.91; N, 13.05; S, 6.08. ESI-MS in positive mode (MeCN*/*MeOH + 1% H_2_O): *m*/*z* 461 [Cu(HL^4^)]^+^. ESI-MS in negative mode (MeCN*/*MeOH + 1% H_2_O): 495 [Cu^II^(L^4^)Cl]^−^. UV–vis, λ_max_ [nm] (ε [M^–1^ cm^–1^]): 258 sh, 275 (10,215), 420 (10,850), ∼625 sh. IR (ATR, selected bands υ̃_max_): 3431, 3253, 3077, 2957, 2859, 2832, 1613, 1562, 1487, 1453, 1409, 1300, 1267, 1207, 1112, 1012, 956, 911, 867, 790 cm^–1^. Crystals suitable for X-ray diffraction measurement were obtained by vapor diffusion of Et_2_O into a solution of the complex in DMF.

##### [Cu(HL^5^)Cl]·2.5H_2_O (**5**)

Yield: 79 mg, 73%. Elem. anal. calcd for C_20_H_24_ClCuN_5_O_2_S·2.5H_2_O (*M*_r_ = 542.54), %: C, 44.28; H, 5.39; N, 12.91; S, 5.91. Found, %: C, 44.44; H, 4.78; N, 12.61; S, 5.71 ESI-MS in positive mode (MeCN*/*MeOH + 1% H_2_O): *m*/*z* 461 [Cu(HL^5^)]^+^, 501 [Cu(HL^5^)MeCN]^+^, 860 [Cu(HL^5^)(H_2_L^5^)]^+^. ESI-MS in negative mode (MeCN*/*MeOH + 1% H_2_O): 495 [Cu^II^(L^5^)Cl]^−^. UV–vis, λ_max_ [nm] (ε [M^–1^ cm^–1^]): 282 (12,065), 421 (10,085), ∼650 sh. IR (ATR, selected bands υ̃_max_): 3261, 3080, 2917, 2852, 2819, 1656, 1603, 1552, 1488, 1443, 1300, 1204, 1127, 1112, 1069, 1034, 1011, 957, 894, 862, 801, 788, 661, 625, 612 cm^–1^. Crystals suitable for X-ray diffraction measurement were obtained by vapor diffusion of Et_2_O into a solution of the complex in DMF.

##### [Cu(HL^6^)Cl]·H_2_O (**6**)

Yield: 65 mg, 63%. Elem. anal. calcd for C_20_H_24_ClCuN_5_O_2_S·H_2_O (*M*_r_ = 515.52), %: C, 46.60; H, 5.08; N, 13.59; S, 6.22. Found, %: C, 46.74; H, 4.79; N, 13.17; S, 6.06. ESI-MS in positive mode (MeCN*/*MeOH + 1% H_2_O): 461 [Cu^II^(HL^6^)]^+^. UV–vis, λ_max_ [nm] (ε [M^–1^ cm^–1^]): 278 (14,500), 320 sh, 428 (11,850), ∼610 sh. IR (ATR, selected bands υ̃_max_): 3240, 3057, 2903, 2868, 2854, 1604, 1533, 1485, 1455, 1398, 1274, 1210, 1142, 1114, 1071, 1025, 993, 955, 914, 871, 809, 782, 736 cm^–1^. Crystals suitable for X-ray diffraction measurement were obtained by slow evaporation of MeOH from the reaction mixture.

### 4.5 Physical Measurements

All compounds are >95% pure by elemental analysis. Elemental analysis was carried out in a Carlo-Erba microanalyzer at the Microanalytical Laboratory at the Faculty of Chemistry, University of Vienna. In addition, >95% purity of hit compounds **3** and **6** was confirmed by HPLC combined with HR ESI mass spectrometry (see Figures S5A and S6B). Analytical HPLC measurements were conducted on a Thermo Scientific Vanquish Horizon UHPLC system using a reverse-phase C18 column (Acclaim Thermo Scientific C18, 120 Å, 2.1 × 150 mm, 3 μm). Milli-Q water containing 0.1% TFA and acetonitrile containing 0.1% TFA were used as eluents with a gradient of 5–100% over 5 min with a flow rate of 0.45 mL min^–1^, with column temperature maintained at 40 °C. HRMALDI MS spectra were acquired on a timsTOF flex ESI/MALDI dual source–trapped ion mobility separation–Qq-TOF mass spectrometer (Bruker Daltonics, Bremen, Germany) in the positive ion mode. The sum formulas of the detected ions were determined using Bruker Compass Data Analysis 5.3 based on the mass accuracy (Δ*m*/*z* ≤5 ppm) and isotopic pattern matching (SmartFormula algorithm). The samples for low-resolution electrospray ionization mass spectrometry (ESI-MS) were measured on an amaZon speed ETD Bruker instrument. Expected and experimental isotope distributions were compared. Optical spectra of **3**–**6** were measured on a Shimadzu 3600 UV–vis–NIR spectrometer (Japan). EPR measurements were performed using an X-band Bruker EMX spectrometer (Germany). IR spectra were recorded on a Bruker Vertex 70 Fourier transform IR spectrophotometer (600–4000 cm^–1^) using the attenuated total reflection (ATR) technique. 1D (^1^H, ^13^C) and 2D (^1^H–^1^H COSY, ^1^H–^13^C HSQC, ^1^H–^13^C HMBC) NMR spectra were acquired on a Bruker AV NEO 500 or AV III 600 spectrometers in DMSO-*d*_*6*_ at 25 °C.

### 4.6 Crystallographic Structure Determination

X-ray diffraction measurements of **H**_**2**_**L**^**3**^, **[H**_**4**_**L**^**4**^**]Cl**_**2**_, **H**_**2**_**L**^**5**^, **H**_**2**_**L**^**6**^, **3**, **4**, **5**, and **6** were performed on Bruker X8 APEX-II CCD and Bruker D8 Venture diffractometers. Single crystals were positioned at 24, 27, 24, 24, 40, 35, 30, and 27 mm from the detector, and 1290, 1632, 454, 1378, 1060, 1138, 2506, and 1235 frames were measured, each for 60, 10, 7, 30, 20, 50, 15, and 8 s over 0.5, 0.5, 0.5, 0.5, 1.5, 0.7, 0.360, and 0.5° scan widths, respectively. Crystal data, data collection parameters, and structure refinement details are given in Tables S1 and S2. The structures were solved by direct methods and refined by full-matrix least-square techniques. Non-H atoms were refined with anisotropic displacement parameters. H atoms were inserted in calculated positions and refined with a riding model. The following computer programs and hardware were used: structure solution, *SHELXS-2014* and refinement, *SHELXL-2014*;^92^ molecular diagrams, *ORTEP*;^93^ computer, Intel Core Duo. CCDC nos.: 2322653 (**H**_**2**_**L**^**3**^), 2322654 (**[H**_**4**_**L**^**4**^**]Cl**_**2**_), 2322655 (**H**_**2**_**L**^**5**^), 2322656 (**H**_**2**_**L**^**6**^), 2322657 (**3**), 2322658 (**4**), 2322659 (**5**), and 2322660 (**6**).

### 4.7 Spectroscopic Studies: UV–vis and ^1^H NMR Titrations, Kinetic Measurements, and Lipophilicity Determination

An Agilent Carry 8454 diode array spectrophotometer was used to record the UV–vis spectra in the interval 200–800 nm. The path length was 1 cm. Proton dissociation constants (p*K*_a_) of the proligands and complexes were calculated by the computer program PSEQUAD.^94^ Spectrophotometric titrations were performed on samples containing the proligands at a 50–100 μM concentration with a KOH solution in the presence of 0.1 M KCl at 25.0 ± 0.1 °C in the pH range from 2 to 11.5 in a 30% (v/v) DMSO/H_2_O solvent mixture, and the metal-to-ligand ratios were 1:0, 1:1, and 1:2. An Orion 710A pH meter equipped with a Metrohm combined electrode (type 6.0234.100) and a Metrohm 665 Dosimat buret were used for the titrations. The electrode system was calibrated to the pH = −log[H+] scale by means of blank titrations (HCl vs KOH) according to the method suggested by Irving et al.^95^ The average water ionization constant (p*K*_w_) is 14.52 ± 0.05 in 30% (v/v) DMSO/H_2_O, which corresponds well to literature data.^59^ Ar was also passed over the solutions during the titrations. Measurements were carried out in the range *ca*. 1.0–2.0 by preparing individual samples, in which KCl was partially or completely replaced by HCl, and pH values were calculated from the strong acid content. The conditional stability constants (*K*′) of the copper(II) complexes were calculated at pH 5.90 based on the spectral changes *via* the displacement reaction with EDTA in the presence 50 mM MES and 0.1 M KCl in water or in 30% (v/v) DMSO/H_2_O. In the competition experiments, the samples contained 25 μM copper(II) and 25 μM proligand, and the concentration of EDTA was varied from 0 to 400 μM. The conditional stability constants of the metal complexes (*K*′) and the individual spectra of the species were calculated by the computer program PSEQUAD.^94^

The redox reaction of the copper(II) complexes with GSH and ascorbic acid was studied at 25.0 ± 0.1 °C on a Hewlett-Packard 8452A diode array spectrophotometer using a special, tightly closed tandem cuvette (Hellma Tandem Cell, 238-QS). The reactants were separated until the reaction was triggered. Both isolated pockets of the cuvette were completely deoxygenated by bubbling a stream of Ar for 10 min before mixing the reactants. Spectra were recorded before and then immediately after the mixing, and changes were followed until no further absorbance change was observed. One of the isolated pockets contained the reducing agent, and the other contained the complex. Their final concentrations were 1250 and 25 μM, respectively. The pH of all the solutions was adjusted to 7.40 by 50 mM HEPES buffer. The ionic strength of the solution was 0.1 M (KCl). The stock solutions of the reducing agents and the complexes were freshly prepared every day. During the calculations, the absorbance (*A*) – time (*t*) curves were fitted and analyzed at the λ_max_ of the complex. (*A*_0_ – *A*_final_) × e^(−*a*×*t*)^ + *A*_final_ was the fitting equation used, where the *A*_0_, *A*_final_, and *a* parameters were refined and accepted at the minimal value of the weighted sum of squared residuals (difference between the measured and calculated absorbance values) at the given wavelength. Then, observed rate constants (*k*_obs_) of the redox reaction were obtained from the data points of the simulated absorbance–time curves as the slope of the ln(*A*/*A*_0_) vs time plots.

^1^H NMR spectroscopic studies were carried out on a Bruker Avance III HD instrument. All spectra were recorded with the WATERGATE water suppression pulse scheme using sodium trimethylsilylpropanesulfonate as internal standard.

We determined distribution coefficient (*D*_7.4_) values by the traditional shake-flask method in *n*-octanol/buffered aqueous solution at pH 7.40 (20 mM phosphate-buffered saline (PBS)) at 25.0 ± 0.2 °C, as described previously in our work.^96^ The stock solution was prepared in *n*-octanol, and the UV–vis spectra of the octanolic solutions were compared.

### 4.8 Cyclic Voltammetry and Spectroelectrochemistry

Cyclic voltammetric experiments with 0.5 mM solutions of copper(II) complexes **3**–**6** in 0.1 M *n*Bu_4_NPF_6_ (puriss quality from Fluka; dried under reduced pressure at 70 °C for 24 h before use) as supporting electrolyte in DMSO (SeccoSolv max. 0.025% H_2_O, Merck) were performed under Ar using a three-electrode arrangement with Pt wire as the counter electrode and Ag wire as the pseudoreference electrode. Glassy carbon or Pt wire served as the working electrode. Ferrocene purchased from Sigma-Aldrich was used as the internal potential standard without further purification. All potentials in voltammetric studies were quoted vs ferricenium/ferrocene (Fc^+^/Fc) as the redox couple. A Heka PG310USB (Lambrecht, Germany) potentiostat with a PotMaster 2.73 software package served for the potential control in voltammetric studies. *In situ* ultraviolet–visible-near-infrared (UV–vis–NIR) spectroelectrochemical measurements were performed on a spectrometer (Avantes, Model AvaSpec-2048 × 14-USB2 in the spectroelectrochemical cell kit (AKSTCKIT3) with the Pt-microstructured honeycomb working electrode, purchased from Pine Research Instrumentation. The cell was positioned in the CUV-UV Cuvette Holder (Ocean Optics) connected to the diode array UV–vis-NIR spectrometer by optical fibers. UV–vis-NIR spectra were processed using the AvaSoft 7.7 software package. Halogen and deuterium lamps were used as light sources (Avantes, Model AvaLight-DH-S-BAL).

### 4.9 ROS Generation

The application of EPR spin trapping experiments involved the spin trapping agent 5,5-dimethyl-1-pyrroline-*N*-oxide (DMPO, Sigma-Aldrich), which was distilled prior to use (70 Pa; 80–90 °C). The solution of DMPO and the studied Cu(II) complex was mixed with H_2_O_2_ to initiate the Fenton-like reaction. EPR spectra were measured 2 min after the addition of H_2_O_2_. The standard settings during EPR spin trapping experiments were microwave frequency ∼9.424 GHz; power of the microwave radiation ∼21.2 mW; sweep width 100 G; center field 3355.6 G; modulation amplitude 1 G; time constant 10.24 ms; and sweep time 20.48 s.

### 4.10 Cell Culture and Fluorescent Protein-Based Viability Assay

The human uterine sarcoma cell lines MES-SA and the doxorubicin selected MES-SA-Dx5 were obtained from ATCC (MES-SA: No. CRL-1976, MES-SA/Dx5: No. CRL-1977) and were used for the intracellular ROS generation studies. For cytotoxicity, we used the fluorescent cell lines MES-SA mCherry and MES-SA/Dx5 eGFP, which were created by lentiviral transductions of the respective genes.^73^ The phenotype of the resistant cells was maintained by 500 nM doxorubicin treatment a week before use and was verified using cytotoxicity assays. Cells were cultivated at 37 °C, 5% CO_2_ in Dulbecco’s modified Eagle's Medium (DMEM, Sigma-Aldrich), supplemented with 10% fetal bovine serum, 5 mM glutamine, and 50 unit/mL penicillin and streptomycin (Thermo Fisher).

### 4.11 Fluorescent Protein-Based Cytotoxicity Assay

MES-SA mCherry and MES-SA/Dx5 eGFP cell suspensions were seeded after trypsinization at 2500 cells/20 μL density on 384-well plates, containing 20 μL of completed medium. The next day, serial dilution of the compounds was prepared and added in an additional 20 μL, with or without 0.4 μM Tariquidar. Liquid handling steps were performed by a Hamilton StarLet robot. After 120 h, the fluorescent intensity of the cells was measured by a PerkinElmer EnSpire plate reader (GFP: 485ex/510em; mCherry: 585ex/610em). pIC_50_ values were computed by our custom program written in C# by calculating the intersection of the cytotoxicity curve and the 50% viability line. The mean and standard deviation of the pIC_50_ values were calculated and converted to IC_50_ values.

### 4.12 dNTP Extraction and Quantification

After a 24 h incubation with or without drugs, MES-SA cells were washed twice with PBS and trypsinized for 5 min. Ice-cold PBS was added to the suspension, and a sample was removed for counting the cells. 10^7^ cells/sample were collected by centrifugation at 3000 rcf at 4 °C for 10 min. The supernatant was replaced with 500 μL of ice-cold 60% methanol. Cells were kept in 60% methanol at least for one night at −20 °C before isolation of the nucleotides. Pellets were resuspended and subjected to a heat shock at 95 °C for 5 min. Samples were centrifuged at 11,000 × *g* for 10 min at 4 °C. The supernatant was transferred into Eppendorf tubes, followed by complete evaporation of the solvent in a vacuum concentrator (Eppendorf Vacufuge Concentrator System) at 45 °C. The pellet containing dNTPs was redissolved in 50 μL of RNase-free water and stored at −20 °C. dNTP-containing samples were measured by a nucleotide incorporation-based fluorescent method.^97^ We used a long synthetic oligonucleotide (197 nt) as a template, which contains a dNTP-detection site, the sole complementary base to that of the dNTP to be quantified directly adjacent to the annealed primer. The rest of the DNA stretch to be amplified serves as a signal amplification sequence. In addition to the limiting amount of dNTP to be quantified, the polymerization reaction mix contained the other three dNTPs in large excess. The double-stranded DNA product is detected by the EvaGreen Dye. The 2X master mix for the DNA synthesis reaction contained the following: 0.275 μM primer (Merck KGaA, Darmstadt, Germany), 0.25 μM template (IDT, Coralville, Iowa, USA), 50 μM dNTP mix (excluding the dNTP to be measured in the given experiment to limit its source to the sample), and 1.25 μM EvaGreen (Biotium). 20 U/mL Q5 High-Fidelity DNA polymerase (New England Biolabs) was added to measure dATP and dTTP, while 10 U/mL was used to measure dGTP and dCTP. The final reaction volume was 10 μL (5 μL sample and 5 μL 2X master mix). We used FrameStar 96 Well Skirted PCR Plates, White Wells, Black Frame. DNA synthesis was performed in a Bio-Rad CFX96 qPCR instrument according to the step-by-step protocol in the Supplementary file of ref (10). Fluorescence amplitude data were extracted from the Bio-Rad CFX Maestro Software and analyzed using Excel and Origin. Data points represent three biological replicates. Each measurement was done in three technical repeats. Significance levels were calculated using the one-way ANOVA method.

### 4.13 Tyrosyl Radical Reduction in mR2 RNR Protein

The tyrosyl radical reduction in mR2 protein by **H**_**2**_**L**^**3**^–**H**_**2**_**L**^**6**^ and **3**–**6** was monitored using EPR spectroscopy at 30 K on a Bruker Elexsys II E540 EPR spectrometer with an Oxford Instruments ER 4112HV helium cryostat, as described previously.^45^ mR2 protein was expressed, purified, and iron-reconstituted, as described previously,^98^ and passed through a 5 mL HiTrap desalting column (GE Healthcare) to remove excess iron. The purified, iron-reconstituted mR2 protein resulted in the formation of 0.76 tyrosyl radical/polypeptide. Samples containing 20 μM mR2 in 50 mM HEPES buffer, pH 7.5/100 mM NaCl, 20 μM compound in 1% (v/v) DMSO/H_2_O, in the absence or presence of 2 mM DTT, were incubated for indicated times and quickly frozen in cold isopentane. The same samples were used for repeated incubations at room temperature. The experiments were performed in duplicate.

### 4.14 Molecular Docking

The compounds were docked into the crystal structure of the R2 subunit of RNR (PDB ID: 1W68; resolution 2.2 Å)^99^ and into the colchicine site of tubulin (PDB ID: 1SA0, resolution 3.58 Å).^88^ The Scigress version FJ 2.6 program^100^ was used to prepare the crystal structure for docking; hydrogen atoms were added, and the crystallographic water molecules were removed. The software was also used to prepare the compounds for docking using the MM2^101^ force field or by entering crystallographic coordinates. The center of the binding pocket was defined (*x* = 102.276, *y* = 87.568, *z* = 80.588)^98^ close to Fe_2_O and the enzymatically essential tyrosyl residue (Tyr177) with a 10 Å radius. The basic amino acids lysine and arginine were defined as protonated, and aspartic and glutamic acids were assumed to be deprotonated. In the case of tubulin, the docking center for the binding pocket was defined as the position of the cocrystallized ligand with 10 Å radius. The GoldScore (GS),^84^ ChemScore (CS),^102,103^ Chem Piecewise Linear Potential (ChemPLP),^104^ and Astex Statistical Potential (ASP)^105^ scoring functions were used to validate the predicted binding modes and relative energies of the ligands using the GOLD v5.4 software suite. The parameter file for GS was augmented for Cu according to Sciortino et al.^85^ The QikProp 4.6^106^ (for the ligands) and Marvin^107^ (for the metal complexes) software packages were used to calculate the molecular descriptors of the compounds. The reliability of QikProp is established for the molecular descriptors.^108^

## Supporting Information Available

NMR spectroscopic data (Figures S1A–S4E), HPLC-HR-MS data (Figures S5A–S6B), crystal data and details of data collection for proligands and Cu(II) complexes (Tables S1 and S2), details of crystal structures (Figures S7 and S8), UV–vis spectra and EPR spectra of complexes **3**–**6** (Figures S9–S11 and Table S3), details of solution speciation studies of **H**_**2**_**L**^**4**^ and complexes **3**–**6** (Figures S12–S14 and Chart S1), electrochemical and spectroelectrochemical details (Figures S15 and S16), ROS generation identified by EPR spectroscopy (Figure S17), cell cycle analysis (Figure S18 and Tables S4 and S5), and details of molecular docking results (Tables S6–S8) (PDF)
PDB IDs for the complexes and their proligands (PDB)
PDB IDs for the complexes and their proligands (PDB)
PDB IDs for the complexes and their proligands (PDB)
PDB IDs for the complexes and their proligands (PDB)
CIF file for the compounds (ZIP)
Molecular formula strings (CSV)

## Supplementary Material

jm4c00259_si_001.pdf

jm4c00259_si_002.pdb

jm4c00259_si_003.pdb

jm4c00259_si_004.pdb

jm4c00259_si_005.pdb

## Notes

This research was supported in part by the Developmental Therapeutics Program in the Division of Cancer Treatment and Diagnosis of the National Cancer Institute, which includes federal funds under Contract No. HHSN261200800001E. The content of this publication does not necessarily reflect the views or policies of the Department of Health and Human Services, nor does mention of trade names, commercial products, or organizations imply endorsement by the U.S. Government.

## Abbreviations

CVs: cyclic voltammograms
DAMA-colchicine: *N*-deacetyl-*N-*(2-mercaptoacetyl)-colchicine
dNDP(s): deoxynucleoside diphosphate(s)
dNTP(s): deoxynucleotide triphosphate(s)
dTTP: deoxythymidine triphosphate
H_2_DCF-DA: 2′,7′-dichlorodihydrofluorescein diacetate
DMPO: 5,5-dimethyl-1-pyrroline-*N*-oxide
DpC, DMSO: dimethyl sulfoxide; di-2-pyridylketone 4-cyclohexyl-4-methyl-3-thiosemicarbazone
EPR: electron paramagnetic resonance
ESI-MS: electrospray ionization mass spectrometry
GSH: glutathione
5-HP: 5-hydroxy-2-formylpyridine thiosemicarbazone
MDR: multidrug resistance
MT(s): microtubule(s)
MTAs: microtubule-targeting agents
PCET: proton-coupled electron transfer
Pgp: P-glycoprotein
mR2 RNR: mouse R2 RNR
RNR: ribonucleotide reductase
SC-XRD: single-crystal X-ray diffraction
TBHP: *tert*-butyl hydroperoxide
TSCs: thiosemicarbazones.
