# Supplementary material for: Copper(II) Complexes with Isomeric Morpholine-Substituted 2-Formylpyridine Thiosemicarbazone Hybrids as Potential Anticancer Drugs Inhibiting Both Ribonucleotide Reductase and Tubulin Polymerization: The Morpholine Position Matters
Source: J Med Chem. 2024 May 21;67(11):9069–90. doi: 10.1021/acs.jmedchem.4c00259 (PMC11181322; doi:10.1021/acs.jmedchem.4c00259)
Supplement: Supplementary file 1 — jm4c00259_si_001.pdf [file jm4c00259_si_001.pdf]

# Supporting Information
# for
# Copper(II) complexes with isomeric morpholine-substituted 2-formylpyridine thiosemicarbazone hybrids as potential anticancer drugs inhibiting both ribonucleotide reductase and tubulin polymerization: the morpholine position matters

Miljan N. M. Milunovic,[a] Kateryna Ohui,[a] Iuliana Besleaga,[a] Tatsiana V. Petrasheuskaya,[b,c] Orsolya Dömötör,[b,c] Éva A. Enyedy,*[,b,c] Denisa Darvasiova,[d] Peter Rapta,[d] Zuzana Barbieriková,[d] Daniel Vegh,[e] Szilárd Tóth,[f] Judit Tóth,[f] Nóra Kucsma,[f] Gergely Szakács,*[,f,g] Ana Popović-Bijelić,[h] Ayesha Zafar,[i] Jóhannes Reynisson,[j] Anatoly D. Shutalev,[k] Ruoli Bai,[l] Ernest Hamel,[l] Vladimir B. Arion*[,a,m]

*[a]Institute of Inorganic Chemistry, University of Vienna, A-1090 Vienna, Austria; [m]Petru Poni Institute of Macromolecular Chemistry*

*[b]Department of Molecular and Analytical Chemistry, Interdisciplinary Excellence Centre, University of Szeged, Dóm tér 7-8, H-6720 Szeged, Hungary*

*[c]MTA-SZTE Lendület Functional Metal Complexes Research Group, University of Szeged, Dóm tér 7, H-6720 Szeged, Hungary*

*[d]Institute of Physical Chemistry and Chemical Physics, Faculty of Chemical and Food Technology, Slovak University of Technology in Bratislava, SK-81237 Bratislava, Slovakia*

*[e]Institute of Organic Chemistry, Faculty of Chemical and Food Technology, Slovak University of Technology in Bratislava, SK-81237 Bratislava, Slovakia*

*[f]Institute of Molecular Life Sciences, HUN-REN Research Centre for Natural Sciences, Hungarian Research Network, Magyar Tudósok körútja 2, H-1117 Budapest, Hungary*

*[g]Center for Cancer Research, Medical University of Vienna, A-1090 Vienna, Austria*

*[h]Faculty of Physical Chemistry, University of Belgrade, 11158 Belgrade, Serbia*

*[i]School of Chemical Sciences, University of Auckland, Private Bag 92019, Auckland 1142, New Zealand*

*[j]School of Pharmacy and Bioengineering, Keele University, Newcastle-under-Lyme, Staffordshire ST5 5BG, United Kingdom*

*[k]N. D. Zelinsky Institute of Organic Chemistry, Russian Academy of Sciences, 119991 Moscow, Russian Federation*

[l]*Molecular Pharmacology Branch, Developmental Therapeutics Program, Division of Cancer Diagnosis and Treatment, National Cancer Institute, Frederick National Laboratory for Cancer Research, National Institutes of Health, Frederick, Maryland 21702, United States*
[m]*Inorganic Polymers Department, "Petru Poni" Institute of Macromolecular Chemistry, Aleea Gr. Ghica Voda 41 A, Iasi 700487, Romania*

*Corresponding authors:
E-mail: enyedy@chem.u-szeged.hu
E-mail: gergely.szakacs@meduniwien.ac.at
E-Mail: vladimir.arion@univie.ac.at

**Table of Contents**

1. NMR Data ........ 4
2. HPLC-HRMS analysis ........ 14
3. Crystallographic data ........ 17
4. UV-vis and EPR spectra of copper(II) complexes ........ 20
5. Details of the investigation of proton dissociation processes of $H_2L^3$–$H_2L^6$ by pH potentiometric and spectroscopic ($^1$H NMR and UV–vis) methods ........ 23
6. Details of solution speciation studies of 3–6 ........ 25
7. Electrochemistry and spectroelectrochemistry ........ 28
8. ROS generation ........ 30
9. Cell cycle analysis ........ 31
10. Molecular docking ........ 32

## 1. NMR Data

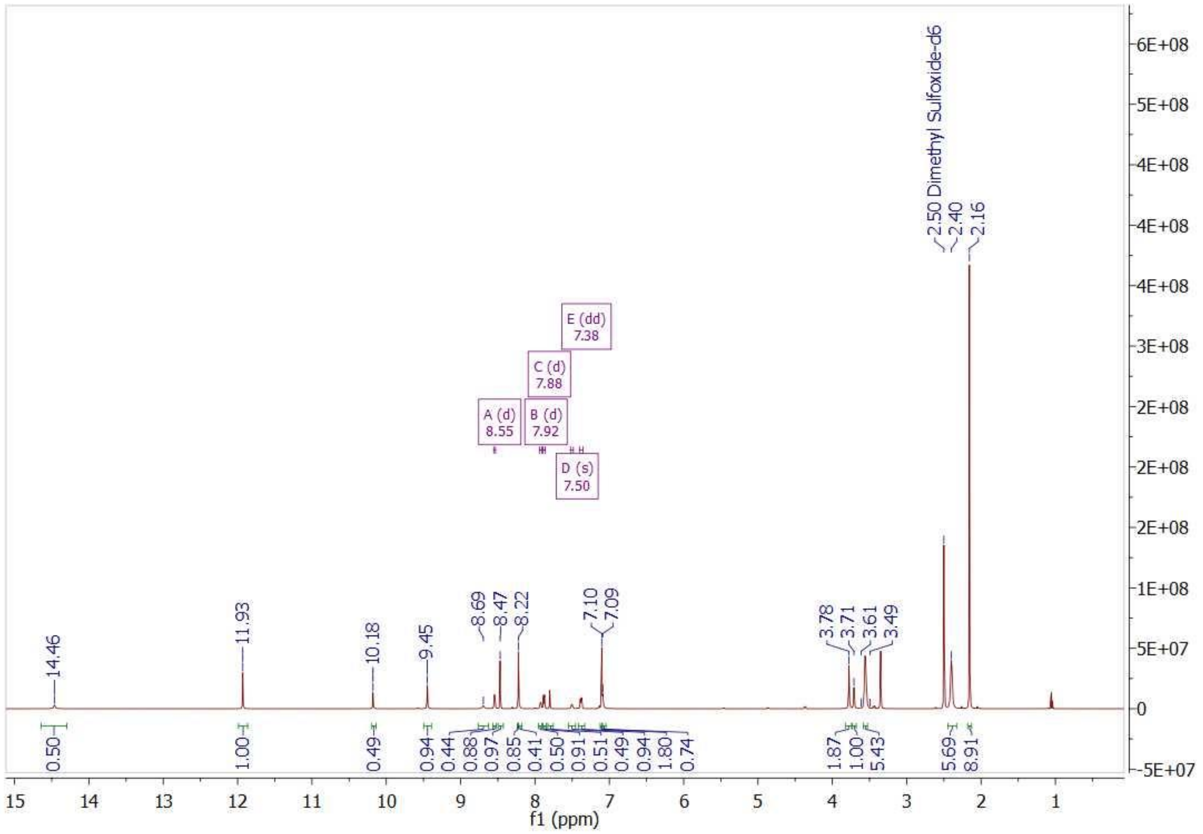


**Figure S1A**. $^{1}H$ NMR spectrum of $\mathbf{H_2L^3}$.

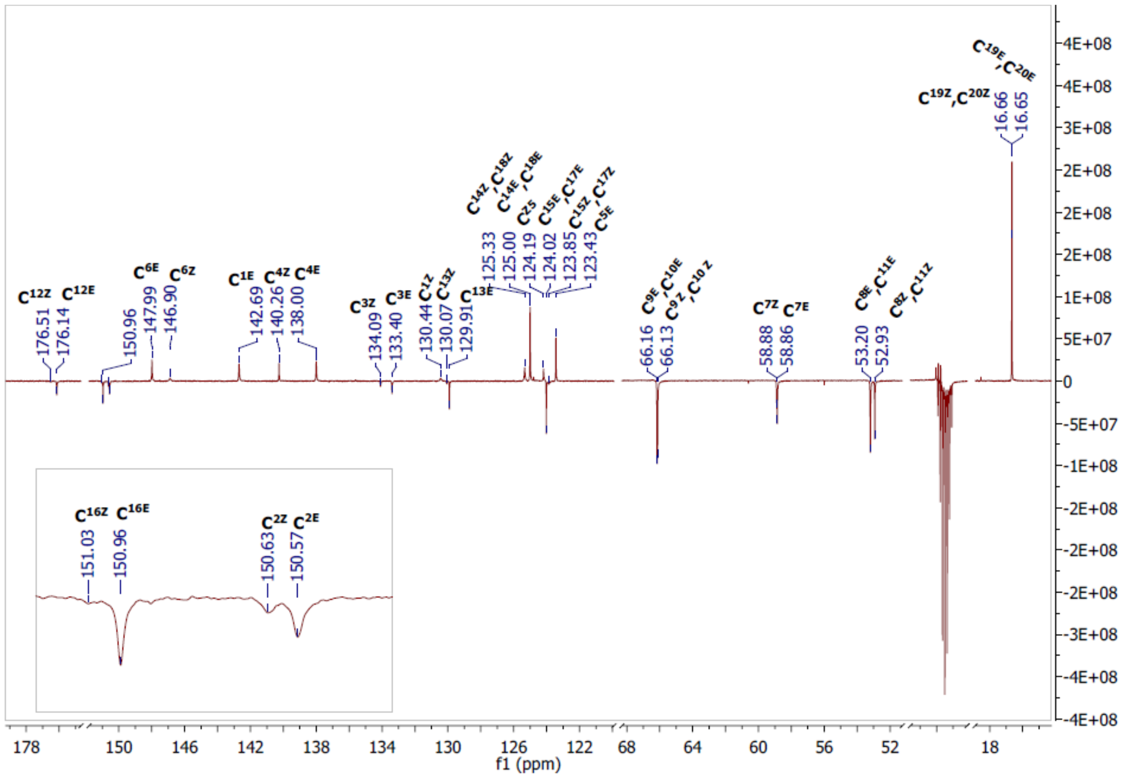


**Figure S1B**. $^{13}C$ NMR spectrum of $\mathbf{H_2L^3}$. The 13C atoms were assigned in agreement with $^{1}H$–$^{13}C$ HSQC, $^{1}H$–$^{13}C$ HMBC and $^{1}H$–$^{1}H$ COSY NMR spectra.

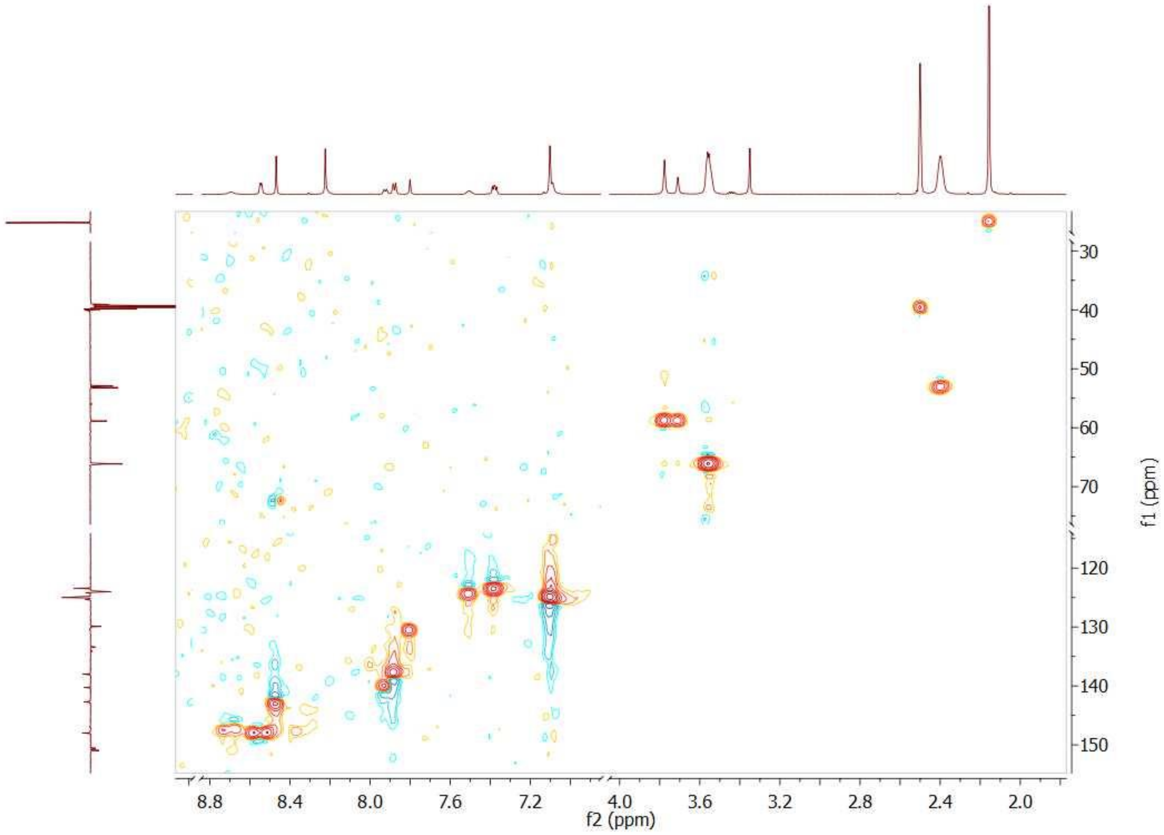


**Figure S1C**. $^{1}$H–$^{13}$C HSQC NMR spectrum of $\mathbf{H_2L^3}$.

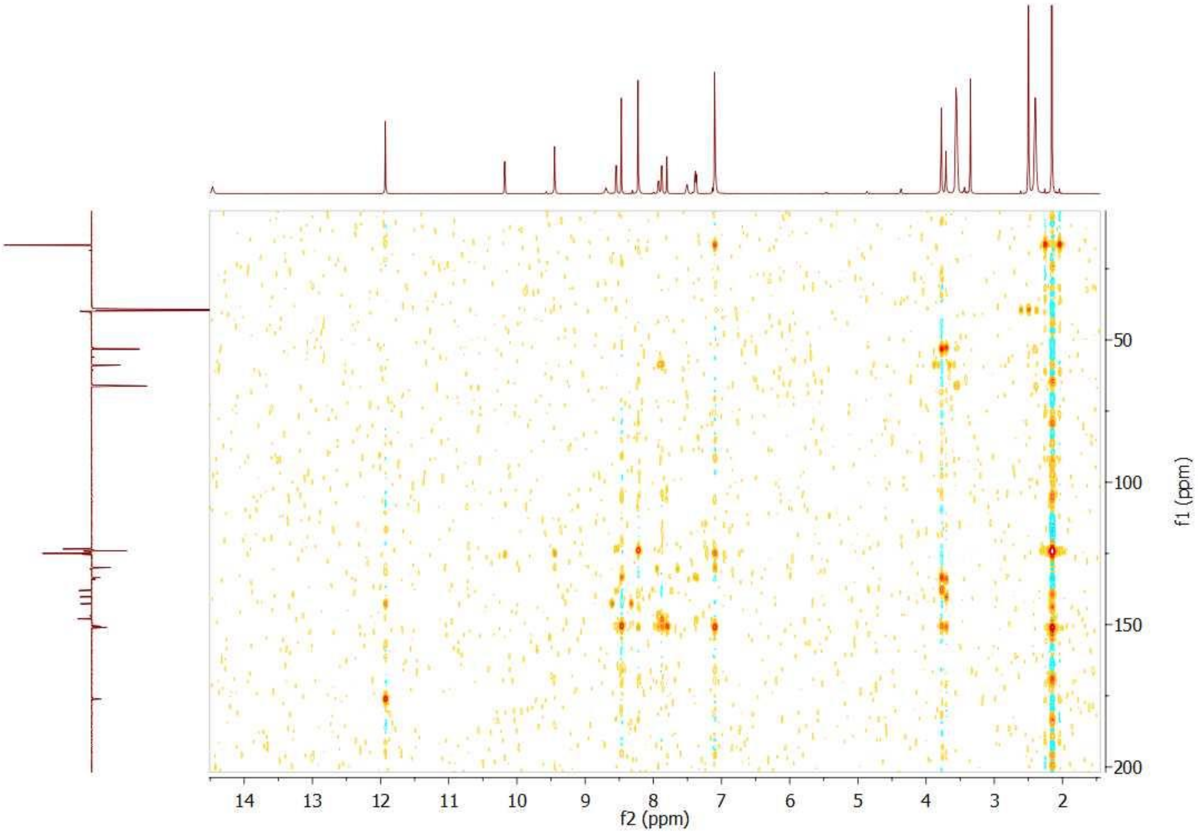


**Figure S1D**. $^{1}$H–$^{13}$C HMBC NMR spectrum of $\mathbf{H_2L^3}$.

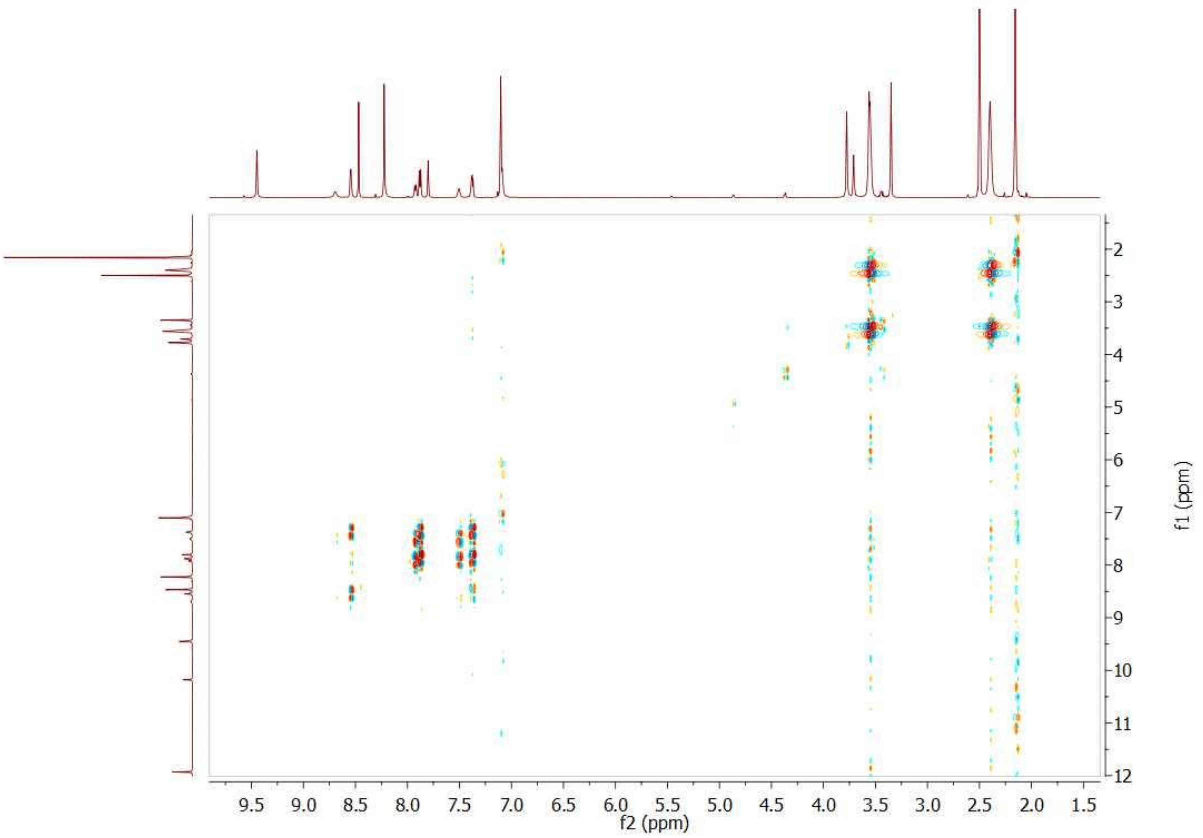


**Figure S1E**. $^{1}H–^{1}H$ COSY NMR spectrum of $\mathbf{H_2L^3}$.

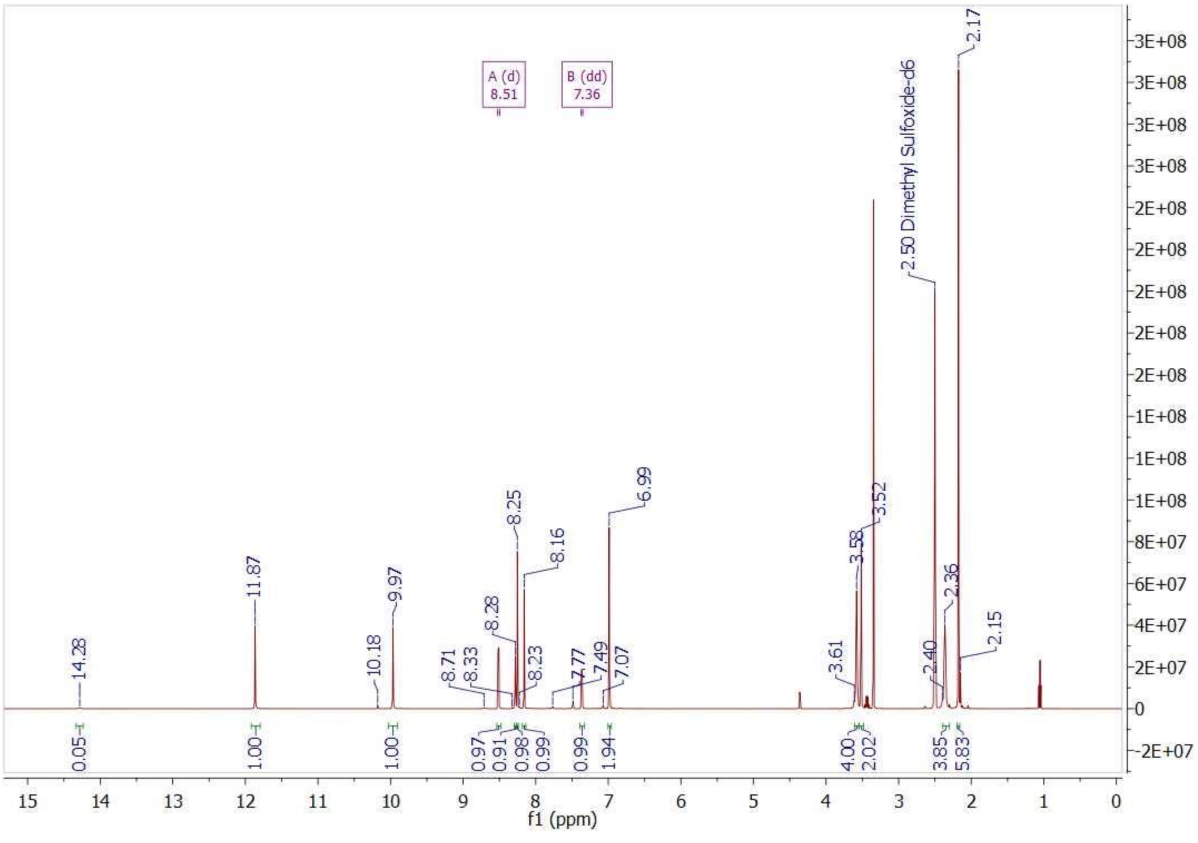


**Figure S2A**. $^{1}H$ NMR spectrum of $\mathbf{H_2L^4}$.

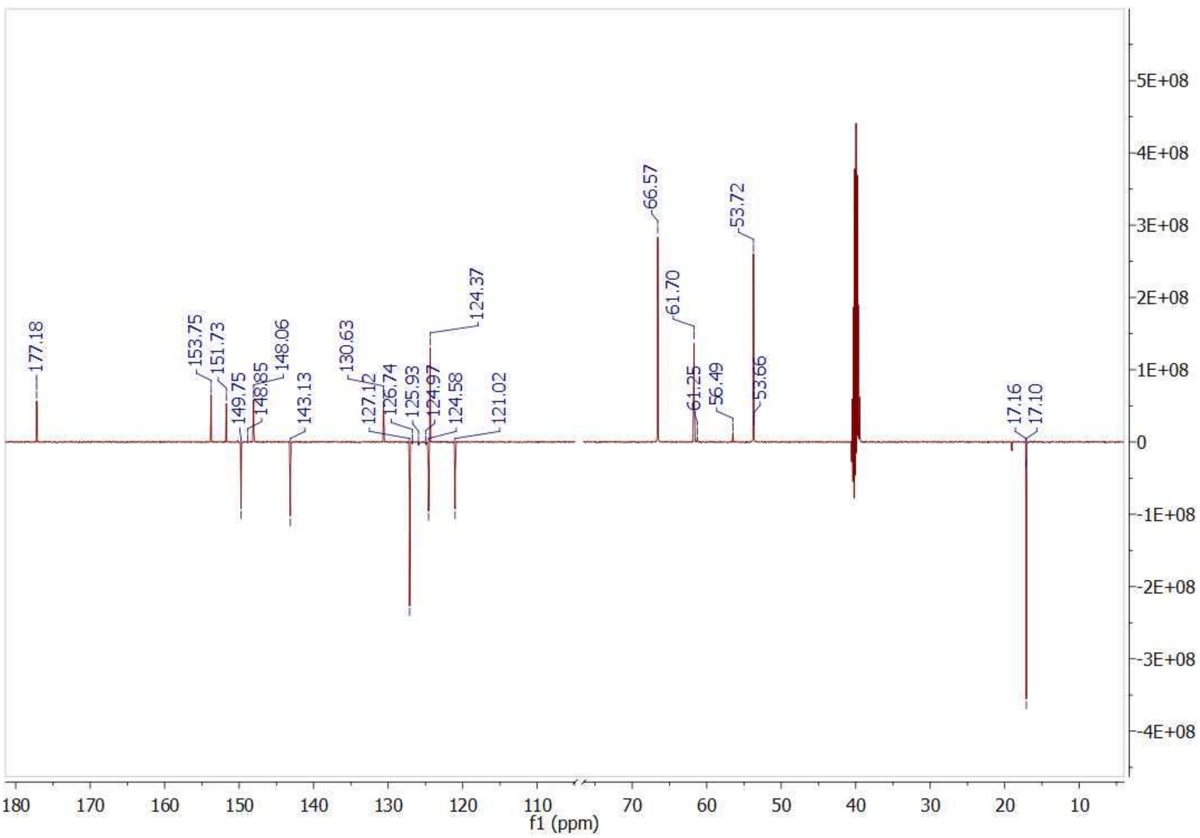


**Figure S2B**. $^{13}$C NMR spectrum of $\mathbf{H_2L^4}$.

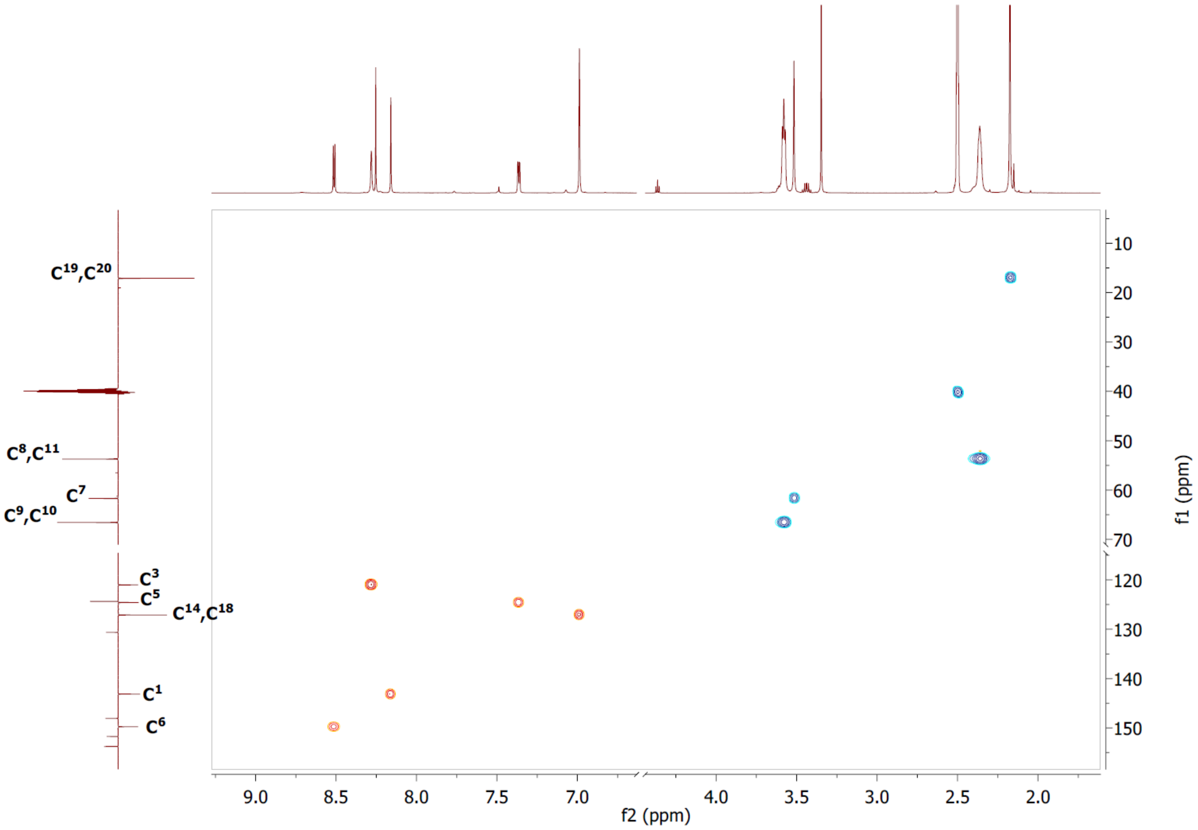


**Figure S2C**. $^{1}$H–$^{13}$C HSQC NMR spectrum of $\mathbf{H_2L^4}$.

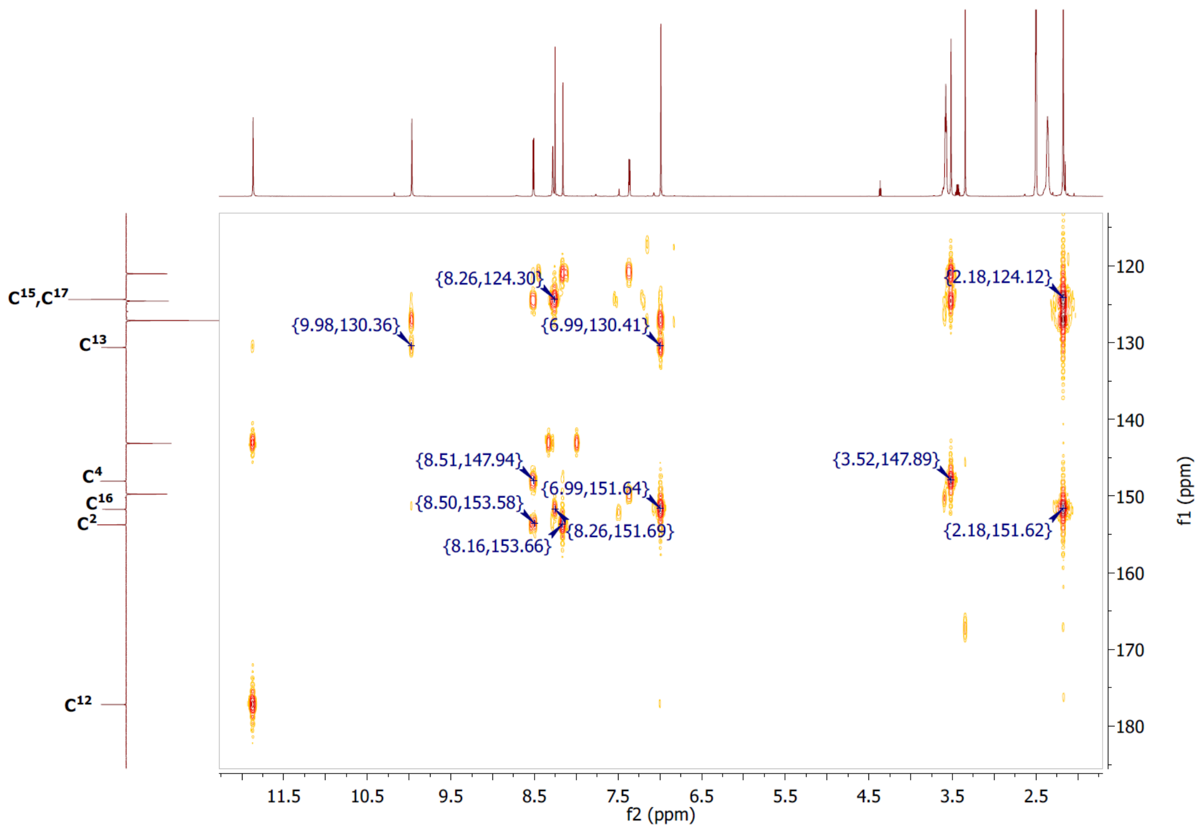


**Figure S2D**. $^{1}$H–$^{13}$C HMBC NMR spectrum of **H$_2$L$^4$**.

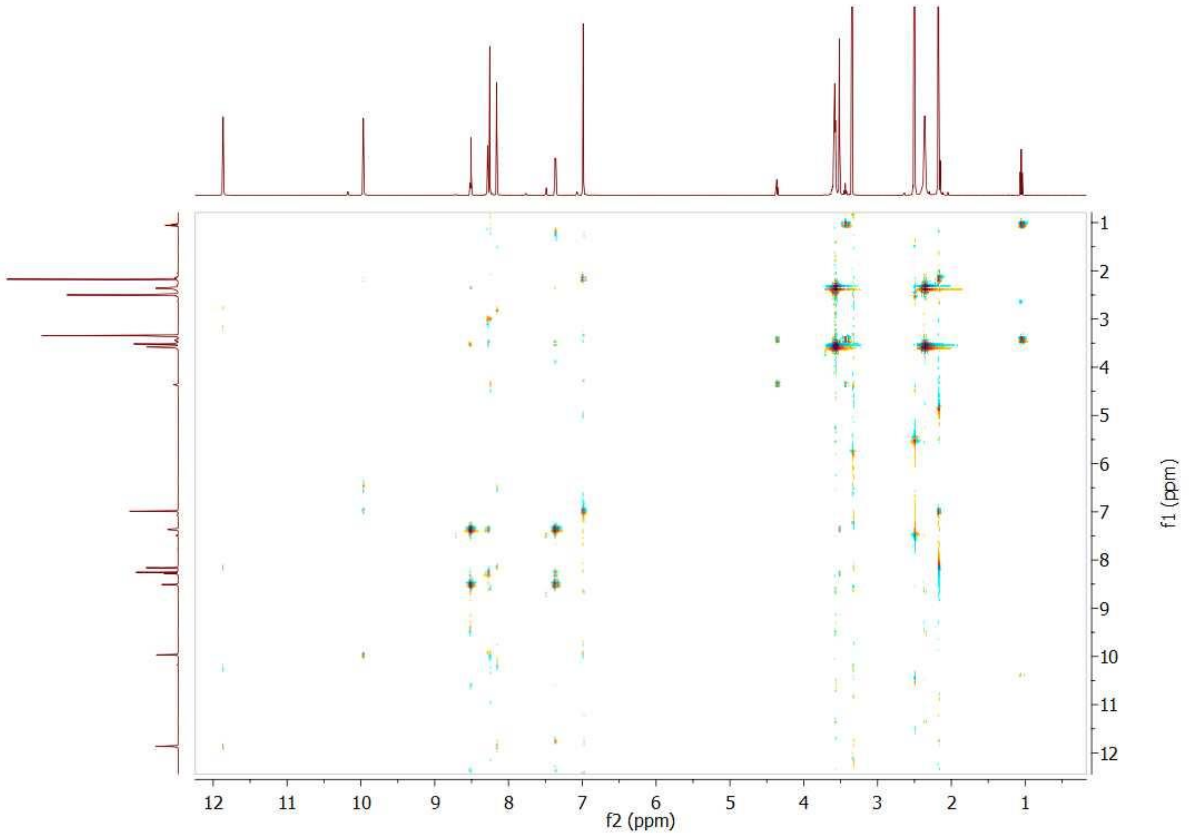


**Figure S2E**. $^{1}$H–$^{1}$H COSY NMR spectrum of **H$_2$L$^4$** (*E*-isomer).

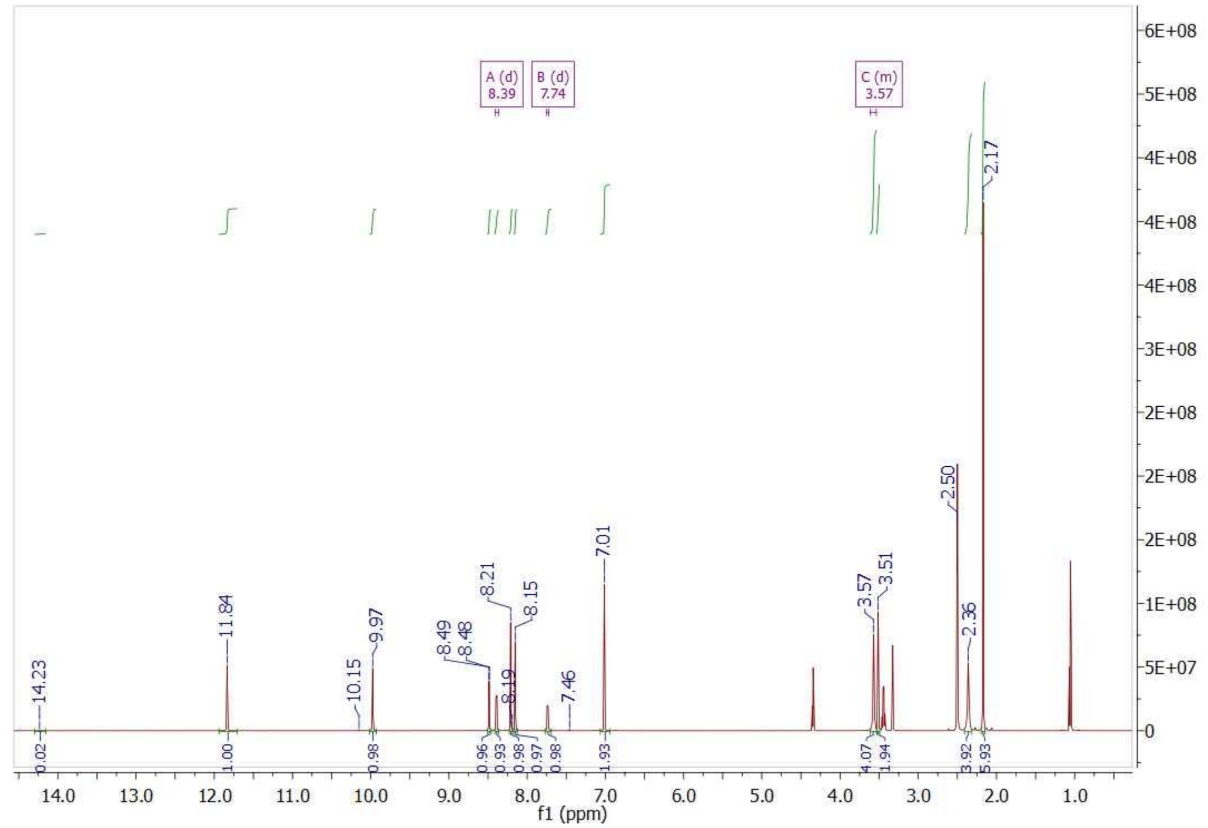


**Figure S3A**. $^{1}$H NMR spectrum of **$H_2L^5$**.

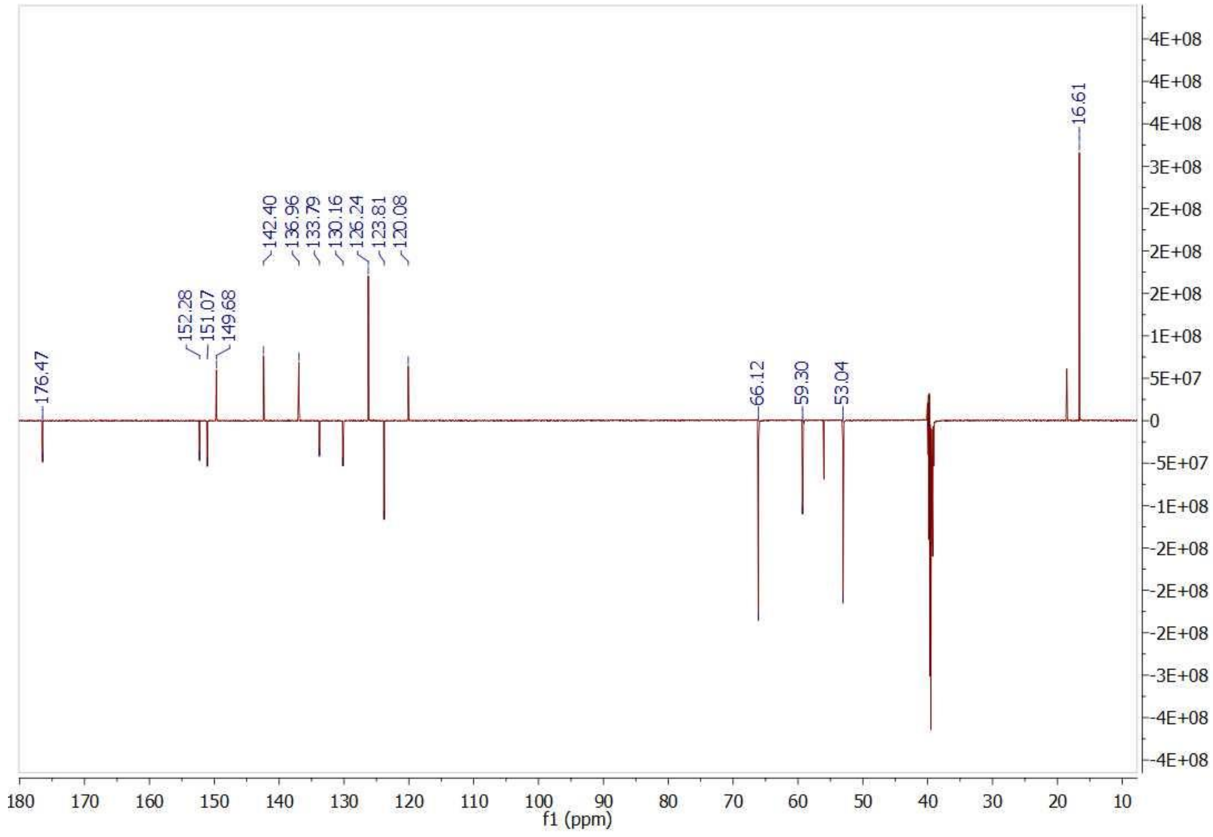


**Figure S3B**. $^{13}$C NMR spectrum of **$H_2L^5$**.

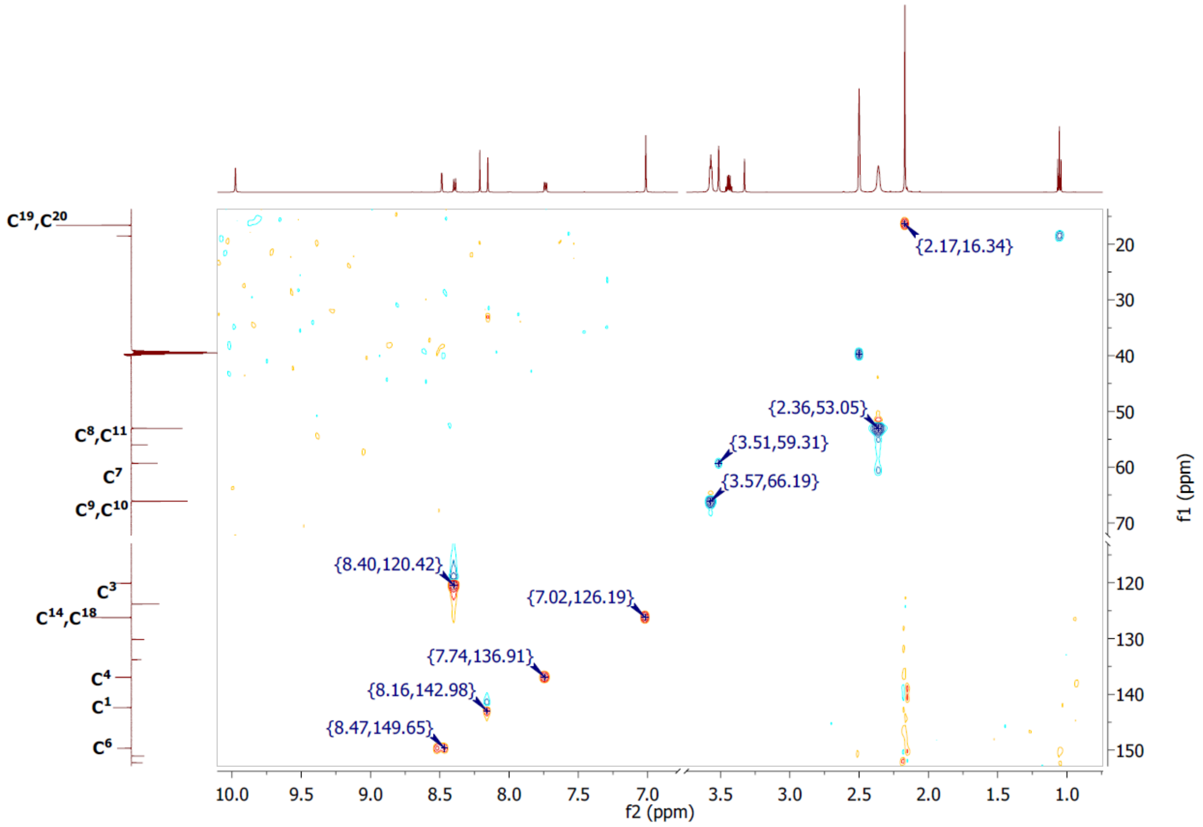


**Figure S3C**. $^{1}H$–$^{13}C$ HSQC NMR spectrum of **$H_2L^5$**.

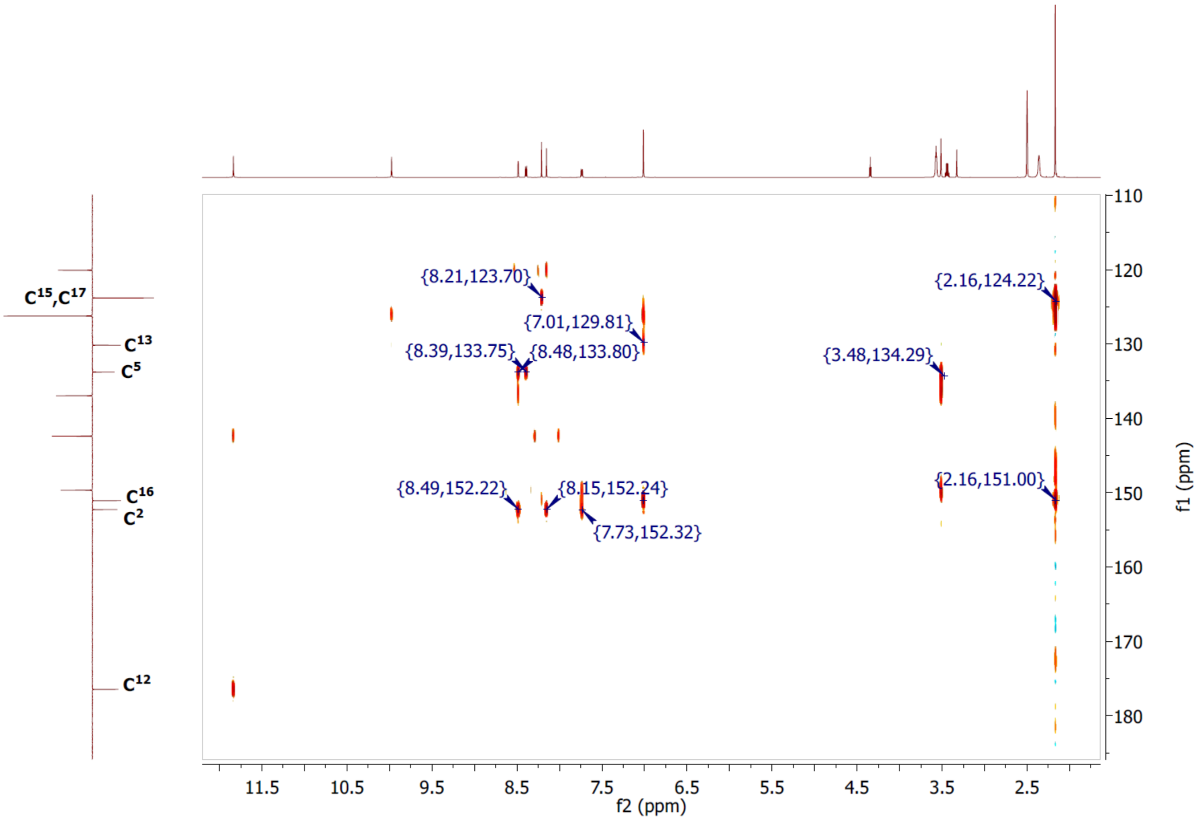


**Figure S3D**. $^{1}H$–$^{13}C$ HMBC NMR spectrum of **$H_2L^5$**.

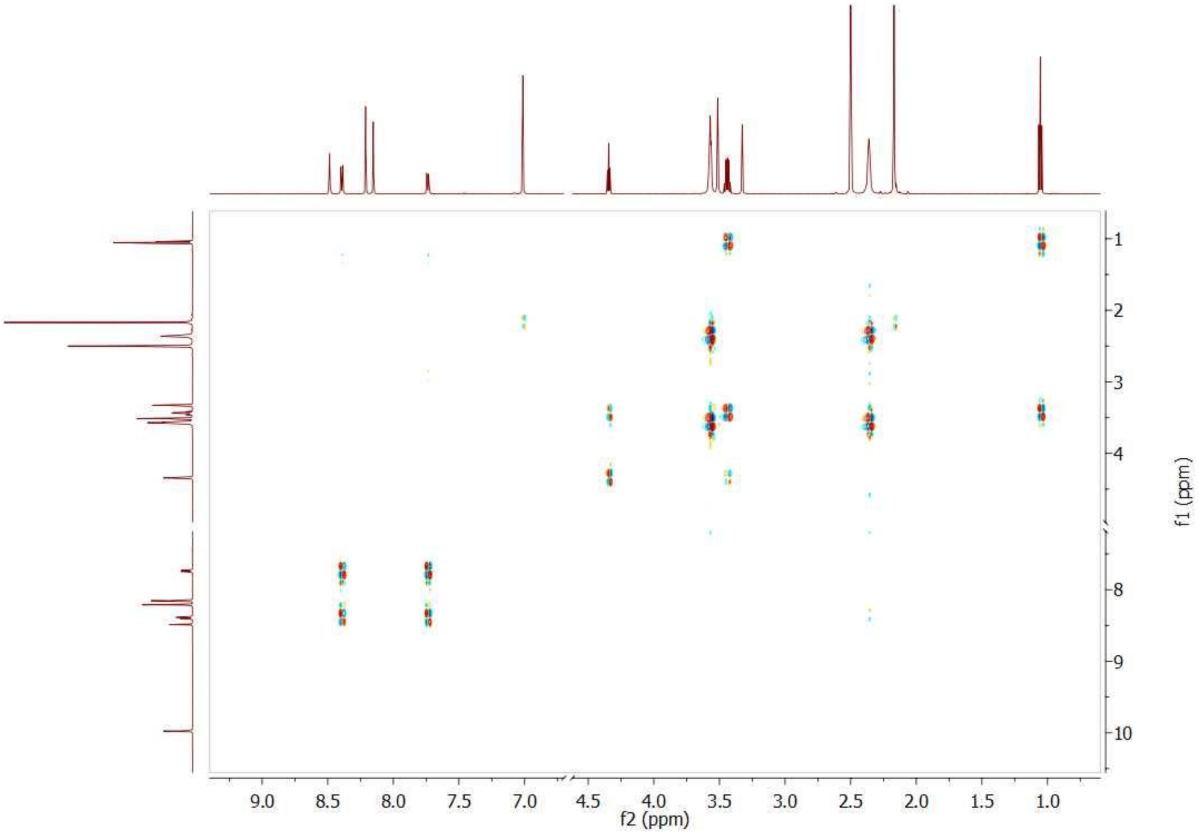


**Figure S3E**. $^{1}H$–$^{1}H$ COSY NMR spectrum of **$H_2L^5$**.

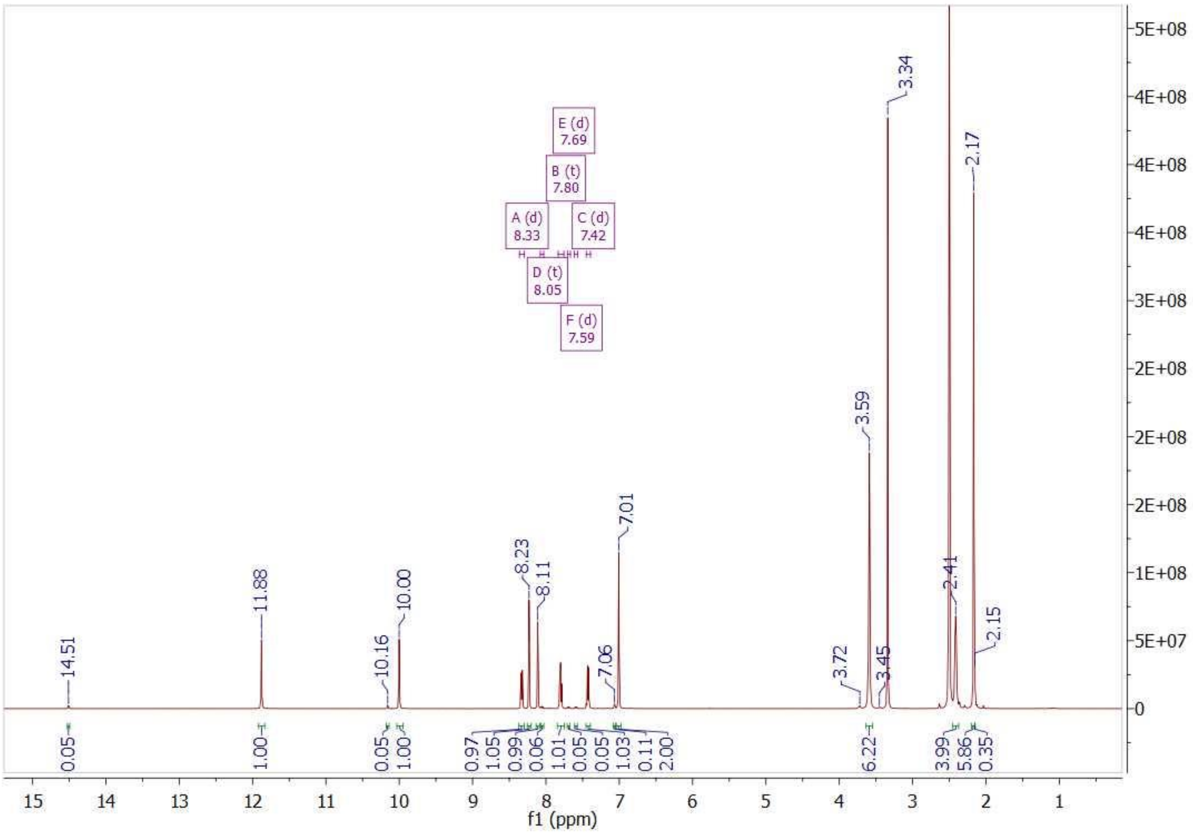


**Figure S4A**. $^{1}H$ NMR spectrum of **$H_2L^6$**.

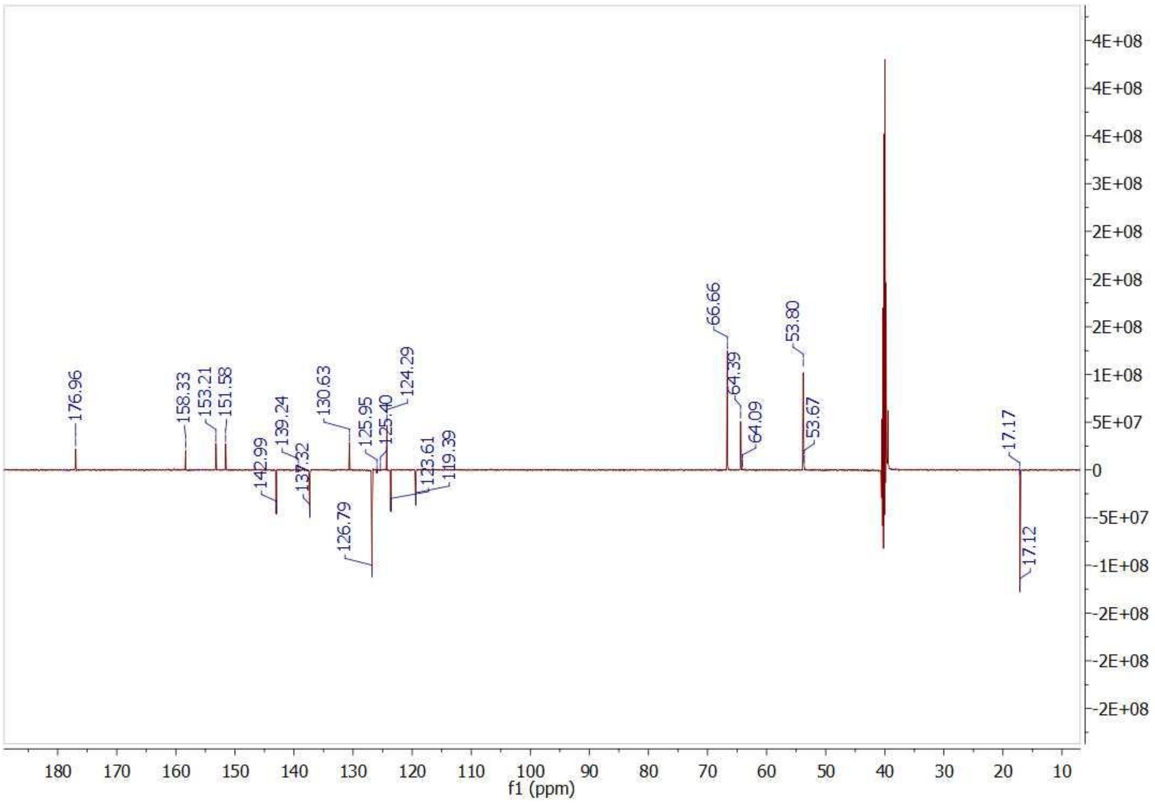


**Figure S4B**. $^{13}$C NMR spectrum of $\mathbf{H_2L^6}$.

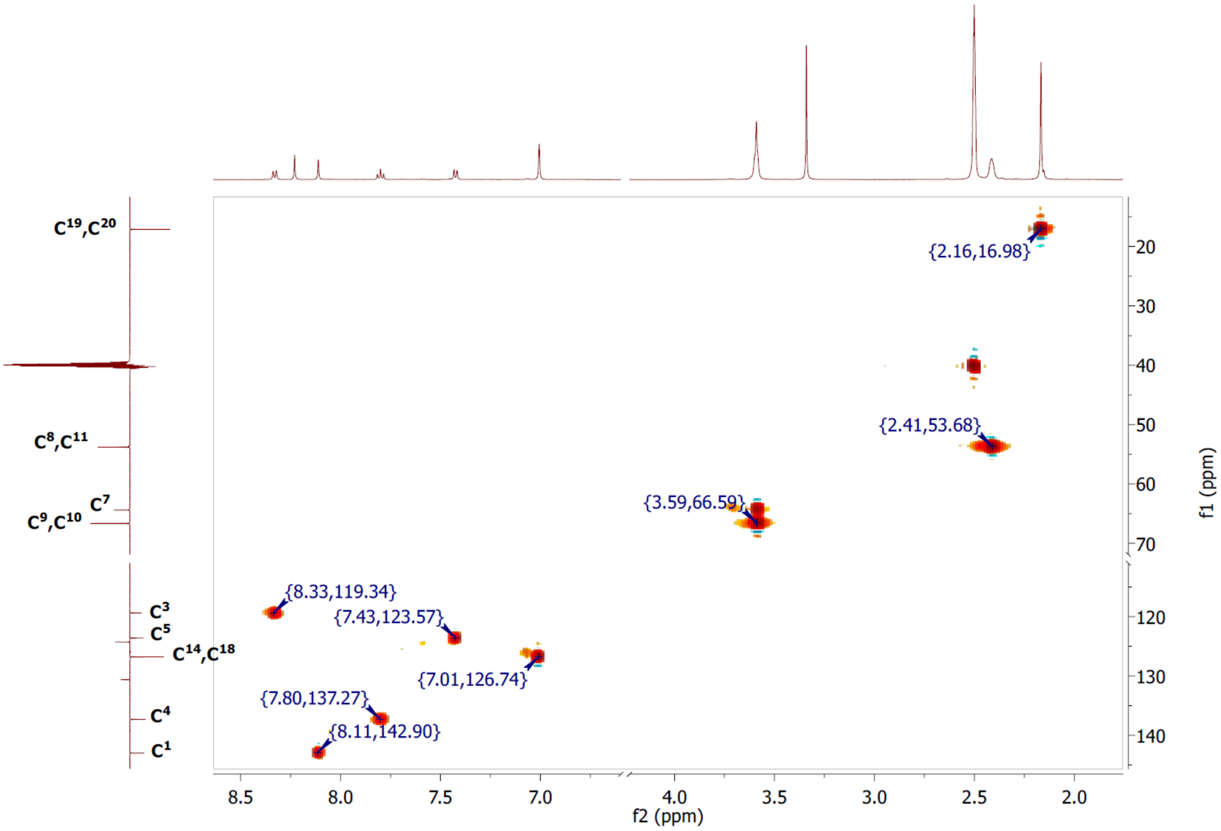


**Figure S4C**. $^{1}$H–$^{13}$C HSQC NMR spectrum of $\mathbf{H_2L^6}$.

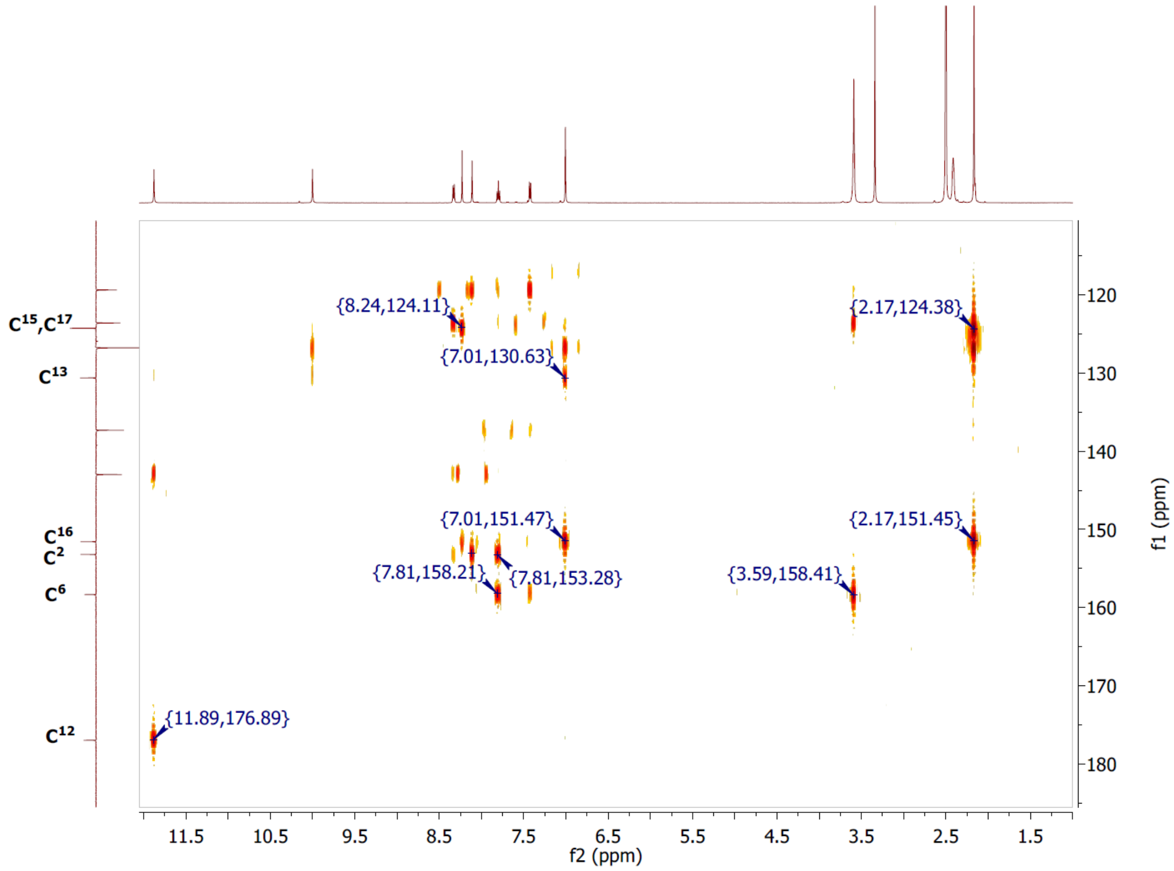


**Figure S4D**. $^{1}H$–$^{13}C$ HMBC NMR spectrum of $\mathbf{H_2L^6}$.

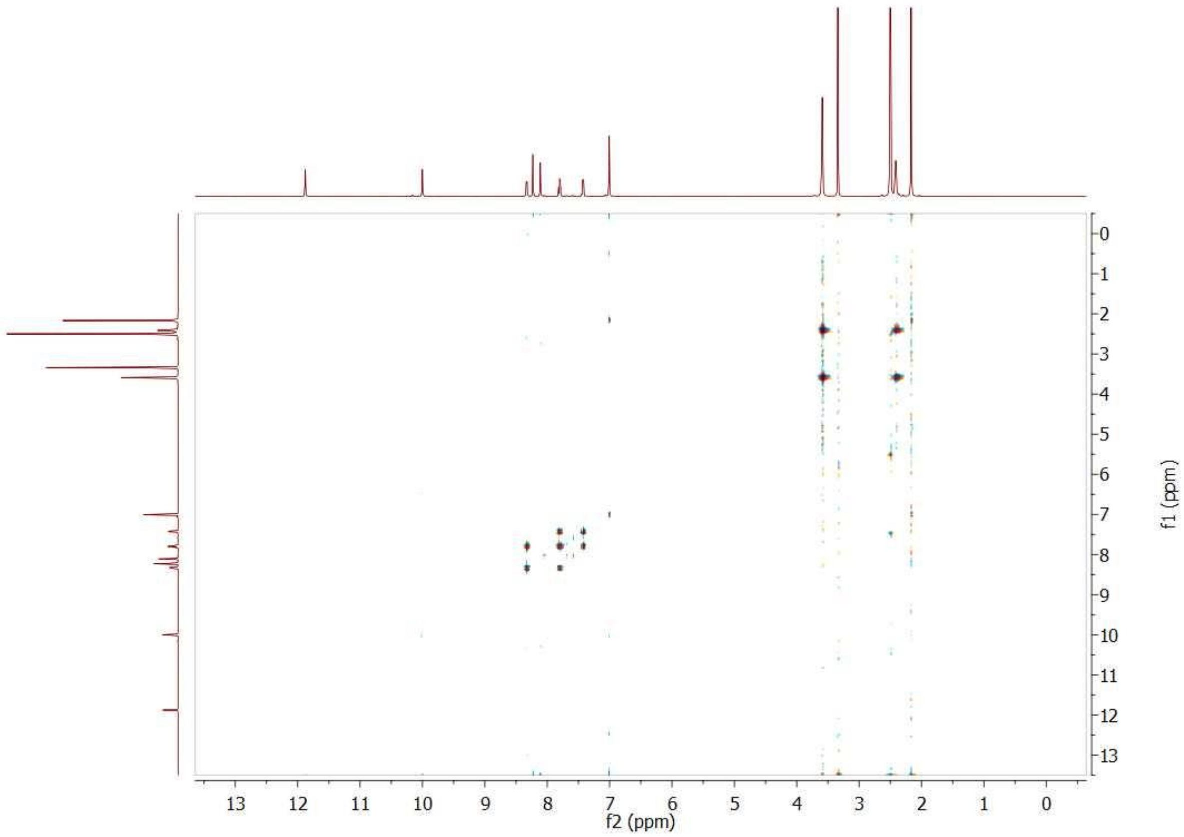


**Figure S4E**. $^{1}H$–$^{1}H$ COSY NMR spectrum of $\mathbf{H_2L^6}$.

## 2. HPLC-HRMS analysis

**Acquisition Parameter**

| | | | | | |
|---|---|---|---|---|---|
| Source Type | ESI | Ion Polarity | Positive | Set Nebulizer | 2.2 Bar |
| Focus | Not active | Set Capillary | 500 V | Set Dry Heater | 220 Â°C |
| Scan Begin | 80 m/z | Set End Plate Offset | -500 V | Set Dry Gas | 10.0 l/min |
| Scan End | 2000 m/z | Set Collision Cell RF | 500.0 Vpp | Set Divert Valve | Waste |

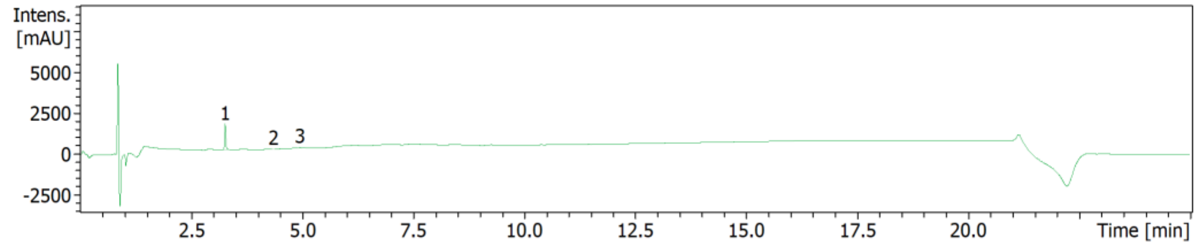


| # | RT [min] | Area | Trace | Area Frac. % |
|---|---|---|---|---|
| 1 | 3.3 | 2854.047 | UV Chromatogram, 190-402 nm | 97.81 |
| 2 | 4.4 | 36.680 | UV Chromatogram, 190-402 nm | 1.26 |
| 3 | 5.0 | 27.262 | UV Chromatogram, 190-402 nm | 0.93 |

**Figure S5A**. HPLC chromatogram of **4** (λ 190–402 nm).

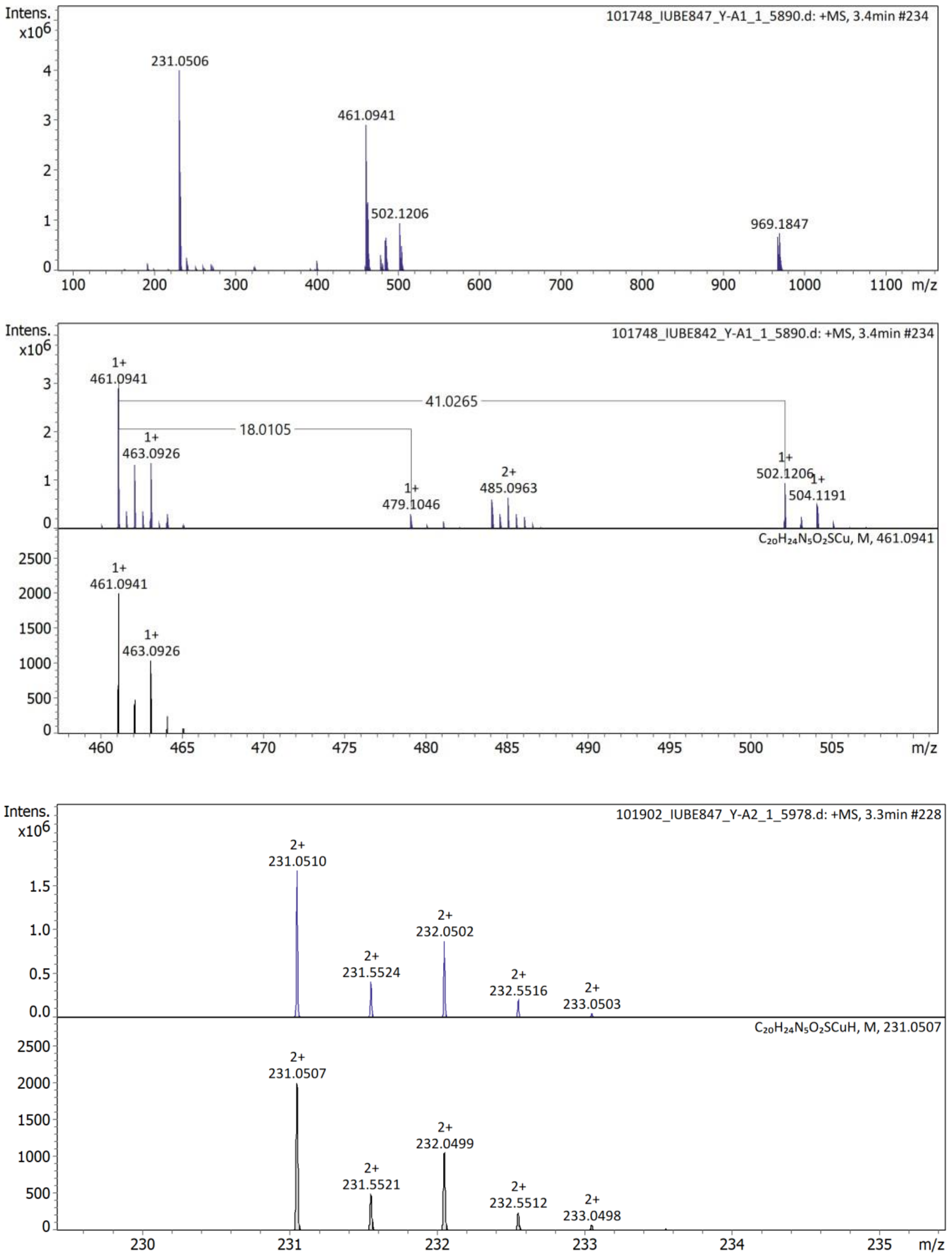


**Figure S5B**. HRMS of the main peak (Rt = 3.3 min) in HPLC chromatogram of **4**

**Acquisition Parameter**

| | | | | | |
|---|---|---|---|---|---|
| Source Type | ESI | Ion Polarity | Positive | Set Nebulizer | 2.2 Bar |
| Focus | Not active | Set Capillary | 500 V | Set Dry Heater | 220 °C |
| Scan Begin | 80 m/z | Set End Plate Offset | -500 V | Set Dry Gas | 10.0 l/min |
| Scan End | 2000 m/z | Set Collision Cell RF | 500.0 Vpp | Set Divert Valve | Waste |

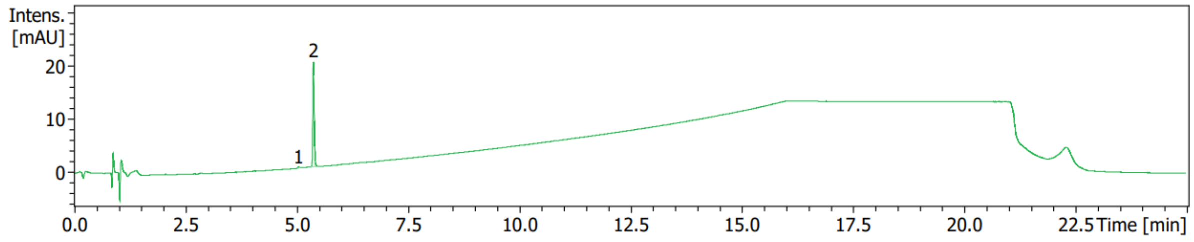


| # | RT [min] | Area | Trace | Area Frac. % |
|---|---|---|---|---|
| 1 | 5.0 | 0.4820 | UV Chromatogram, 254 nm | 1.08 |
| 2 | 5.4 | 44.3013 | UV Chromatogram, 254 nm | 98.92 |

**Figure S6A**. HPLC chromatogram of **6** at λ 254 nm.

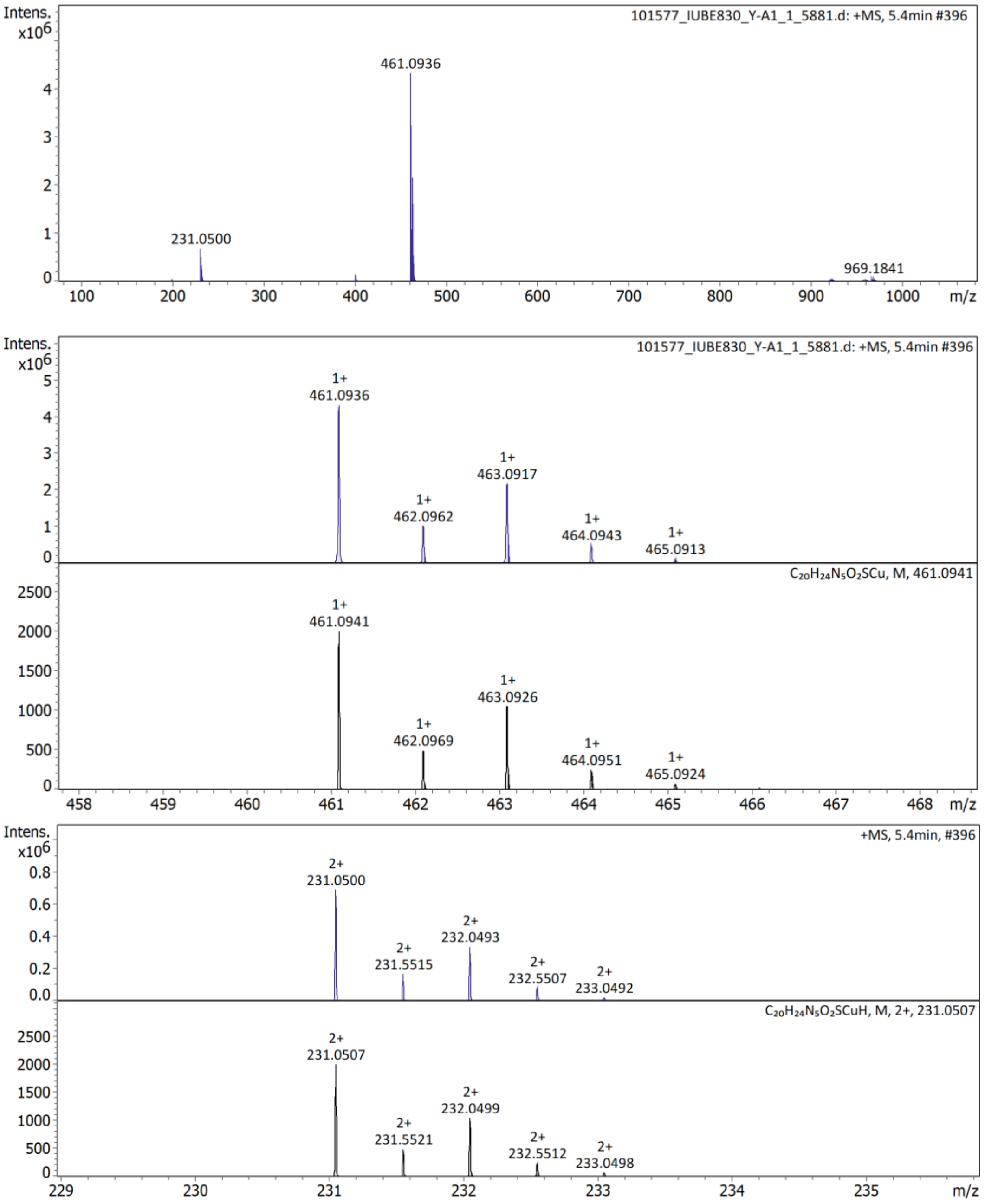


**Figure S6B**. HRMS of the main peak (Rt = 5.4 min) in HPLC chromatogram of **6**.

## 3. Crystallographic data

**Table S1.** Crystal Data and Details of Data Collection and Refinement for **$H_2L^3$·1.5EtOH**, **[$H_4L^4$]$Cl_2$·0.5EtOH·1.5$H_2O$**, **$H_2L^5$·EtOH**, **$H_2L^6$·0.75EtOH**.

| compound | **$H_2L^3$·1.5EtOH** | **[$H_4L^4$]$Cl_2$·0.5EtOH·1.5$H_2O$** | **$H_2L^5$·EtOH** | **$H_2L^6$·0.75EtOH** |
|---|---|---|---|---|
| empirical formula | $C_{23}H_{34}N_5O_{3.5}S$ | $C_{21}H_{33}Cl_2N_5O_4S$ | $C_{22}H_{31}N_5O_3S$ | $C_{21.5}H_{29.5}N_5O_{2.75}S$ |
| fw | 468.61 | 522.48 | 445.58 | 434.06 |
| space group | $P\bar{1}$ | $P\bar{1}$ | $P2_1/n$ | $P2_1/n$ |
| $a$, Å | 9.7366(3) | 10.2216(2) | 12.9352(14) | 12.2108(5) |
| $b$, Å | 16.0139(6) | 14.1219(3) | 9.3015(12) | 8.9891(4) |
| $c$, Å | 17.4120(7) | 19.0545(3) | 19.932(2) | 20.9978(8) |
| $\alpha$, ° | 107.5669(14) | 90.5359(8) | | |
| $\beta$, ° | 103.8625(13) | 98.6623(8) | 98.112(5) | 96.6348(17) |
| $\gamma$, ° | 98.7243(14) | 108.6918(7) | | |
| $V$ [Å$^3$] | 2438.20(16) | 2570.81(9) | 2374.1(5) | 2289.37(16) |
| $Z$ | 4 | 4 | 4 | 4 |
| $\lambda$ [Å] | 0.71073 | 0.71073 | 0.71073 | 0.71073 |
| $\rho_{calcd}$, g cm$^{-3}$ | 1.277 | 1.350 | 1.247 | 1.259 |
| cryst size, mm$^3$ | 0.32 × 0.09 × 0.02 | 0.20 × 0.08 × 0.06 | 0.25 × 0.165 × 0.026 | 0.16 × 0.11 × 0.02 |
| $T$ [K] | 100(2) | 100(2) | 100(2) | 100(2) |
| $\mu$, mm$^{-1}$ | 0.169 | 0.370 | 0.168 | 0.172 |
| $R_1{}^a$ | 0.0432 | 0.0370 | 0.0429 | 0.0578 |
| $wR_2{}^b$ | 0.1065 | 0.1005 | 0.1005 | 0.1576 |
| GOF$^c$ | 1.014 | 1.045 | 1.029 | 1.021 |
| CCDC no. | 2322653 | 2322654 | 2322655 | 2322656 |

$^aR_1 = \Sigma||F_o| - |F_c||/\Sigma|F_o|$. $^b wR_2 = \{\Sigma[w(F_o^2 - F_c^2)^2]/\Sigma[w(F_o^2)^2]\}^{1/2}$. $^c GOF = \{\Sigma[w(F_o^2 - F_c^2)^2]/(n - p)\}^{1/2}$, where n is the number of reflections and p is the total number of parameters refined.

**Table S2.** Crystal Data and Details of Data Collection and Refinement for **[Cu(HL$^3$)Cl]·0.5DMF**, **[Cu(HL$^4$)Cl]**, **[Cu(HL$^5$)Cl]·MeOH·0.4H$_2$O** and **[Cu(HL$^6$)Cl]·1.87MeOH·0.13H$_2$O**.

| compound | **[Cu(HL$^3$)Cl]** | **[Cu(HL$^4$)Cl]** | **[Cu(HL$^5$)Cl]·MeOH·0.4H$_2$O** | **[Cu(HL$^6$)Cl]·1.87MeOH·0.13H$_2$O** |
|---|---|---|---|---|
| empirical formula | $C_{43}H_{55}Cl_2Cu_2N_{11}O_5S_2$ | $C_{20}H_{24}ClCuN_5O_2S$ | $C_{21}H_{28}ClCuN_5O_3S$ | $C_{21.87}H_{31.74}ClCuN_5O_4S$ |
| fw | 1068.08 | 497.49 | 536.74 | 559.75 |
| space group | $P\bar{1}$ | $P\bar{1}$ | $P2_1/n$ | $P\bar{1}$ |
| $a$, Å | 9.5954(7) | 10.0025(12) | 8.082(2) | 13.4360(12) |
| $b$, Å | 16.1126(12) | 13.1949(16) | 16.259(6) | 13.9058(13) |
| $c$, Å | 16.2333(13) | 17.489(2) | 18.049(9) | 14.0308(15) |
| $\alpha$, ° | 80.580(3) | 70.673(4) | | 100.695(4) |
| $\beta$, ° | 84.525(3) | 85.938(4) | 99.172(16) | 96.549(4) |
| $\gamma$, ° | 74.126(3) | 88.204(4) | | 107.412(3) |
| $V$ [Å$^3$] | 2386.5(4) | 2172.6(5) | 2341.5(16) | 2417.4(4) |
| $Z$ | 2 | 4 | 4 | 4 |
| $\lambda$ [Å] | 0.71073 | 0.71073 | 0.71073 | 0.71073 |
| $\rho_{calcd}$, g cm$^{-3}$ | 1.486 | 1.521 | 1.502 | 1.538 |
| cryst size, mm$^3$ | 0.22 × 0.20 × 0.01 | 0.15 × 0.12 × 0.02 | 0.07 × 0.05 × 0.03 | 0.18 × 0.10 × 0.07 |
| $T$ [K] | 200(2) | 100(2) | 100(2) | 100(2) |
| $\mu$, mm$^{-1}$ | 1.147 | 1.252 | 1.172 | 1.140 |
| $R_1{}^a$ | 0.0341 | 0.0553 | 0.0467 | 0.0294 |
| $wR_2{}^b$ | 0.0974 | 0.1339 | 0.1150 | 0.0716 |
| GOF$^c$ | 1.022 | 1.015 | 1.038 | 1.020 |
| CCDC no. | 2322657 | 2322658 | 2322659 | 2322660 |

$^aR_1 = \Sigma||F_o| - |F_c||/\Sigma|F_o|$. $^bwR_2 = \{\Sigma[w(F_o^2 - F_c^2)^2]/\Sigma[w(F_o^2)^2]\}^{1/2}$. $^c$GOF $= \{\Sigma[w(F_o^2 - F_c^2)^2]/(n - p)\}^{1/2}$, where $n$ is the number of reflections and $p$ is the total number of parameters refined.

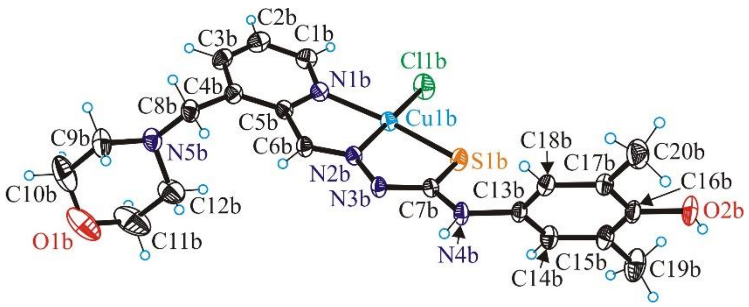


**Figure S7**. ORTEP view of one of the two crystallographically independent molecules of [Cu(L$^3$)Cl)] **(3B)** with thermal ellipsoids at the 50% probability level. Interstitial solvent (DMF) was omitted for clarity.

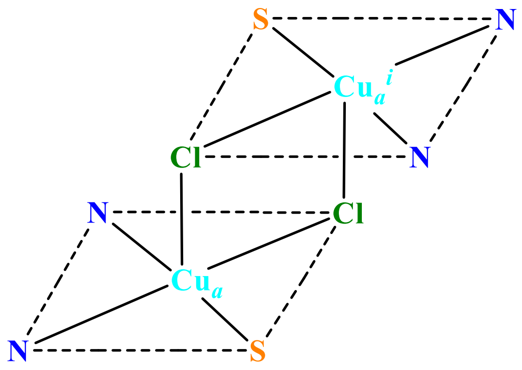


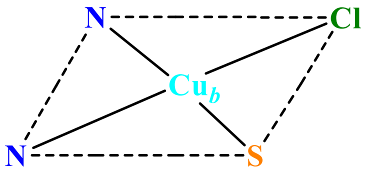


Molecule B

Molecule A

**Figure S8**. The crystallographically independent molecule A of complex **3** is associated in a centrosymmetric dimer, while molecule B is discrete in the crystal and is not involved in any binding intermolecular interactions.

## 4. UV-vis and EPR spectra of copper(II) complexes

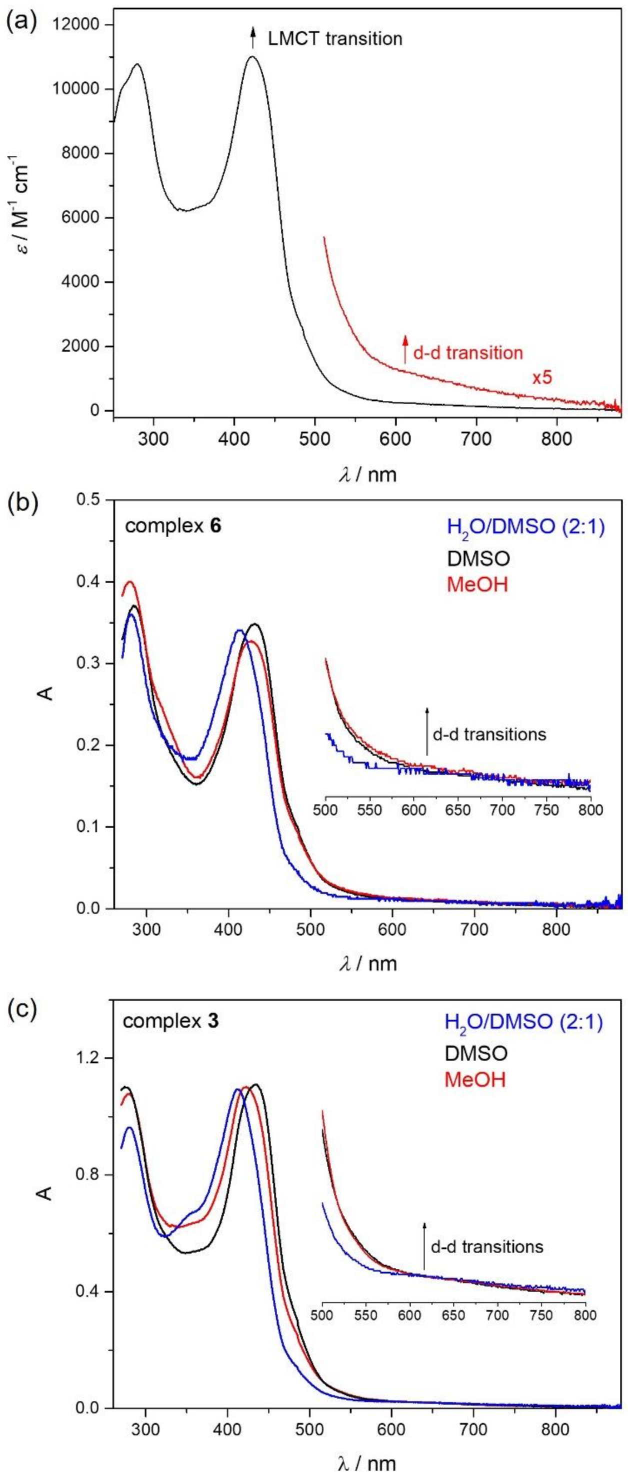


**Figure S9.** (a) UV–vis absorption spectrum of **6** in methanol (inset: expanded region with d-d transitions) and comparison of UV–vis spectra of **6** (b) and **3** (c) in different solvents (30% DMSO/$H_2O$ solvent mixture – blue traces, DMSO – black traces, methanol – red traces).

**Table S3.** Spin–Hamiltonian parameters for **3–6**

| Complex | $g_{\|}$ | $g_{\perp}$ | $A_{\|}$ (G) | $A_{\perp}$ (G) |
|---|---|---|---|---|
| **3** | 2.195±0.003 | 2.053±0.003 | 175 | 20 |
| **4** | 2.195±0.003 | 2.053±0.003 | 175 | 20 |
| **5** | 2.195±0.003 | 2.053±0.003 | 175 | 20 |
| **6** | 2.176±0.003 | 2.048±0.003 | 157 | 14 |

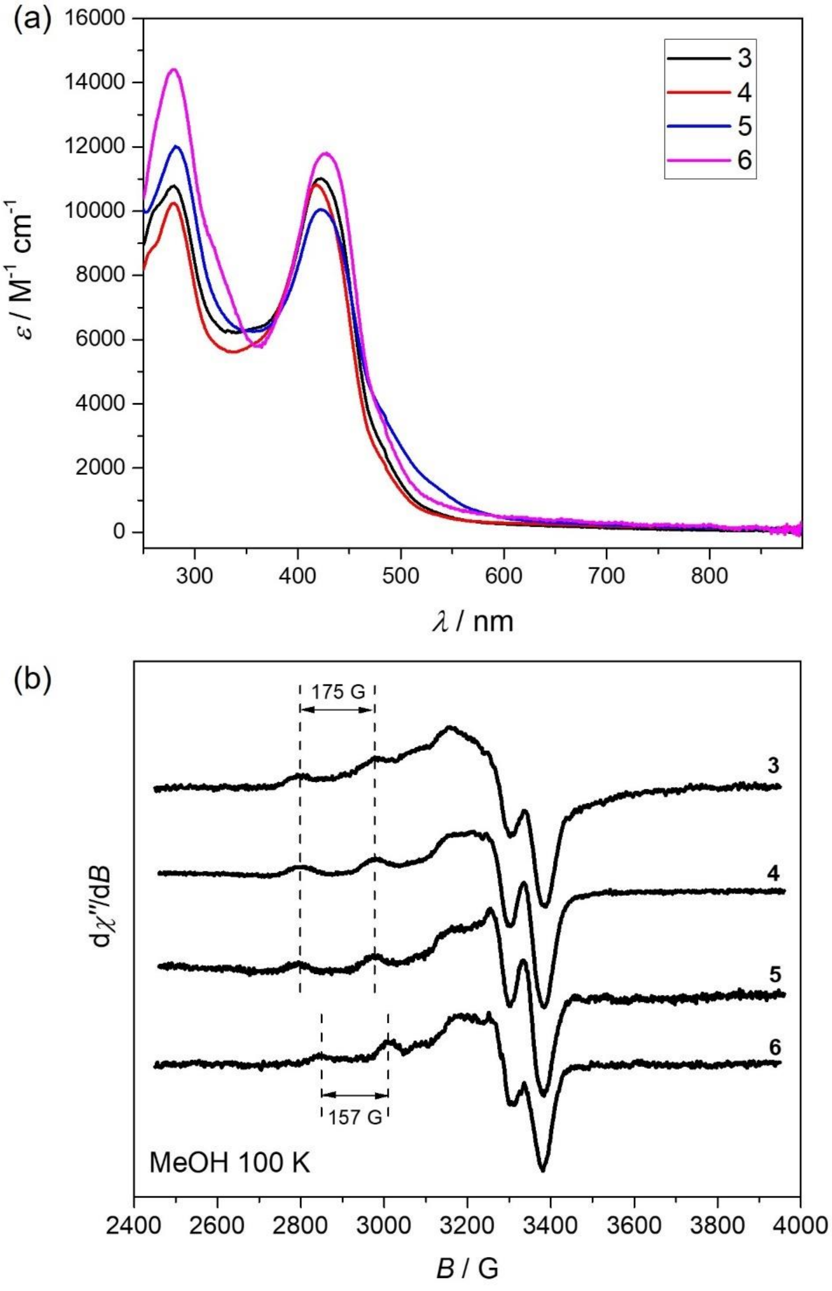


**Figure S10**. (a) UV–vis spectra of **3**–**6** in methanol and (b) EPR spectra of frozen solutions of **3**–**6** in methanol at 100 K. Experimental settings: modulation amplitude – 5 G; microwave power – 2 mW.

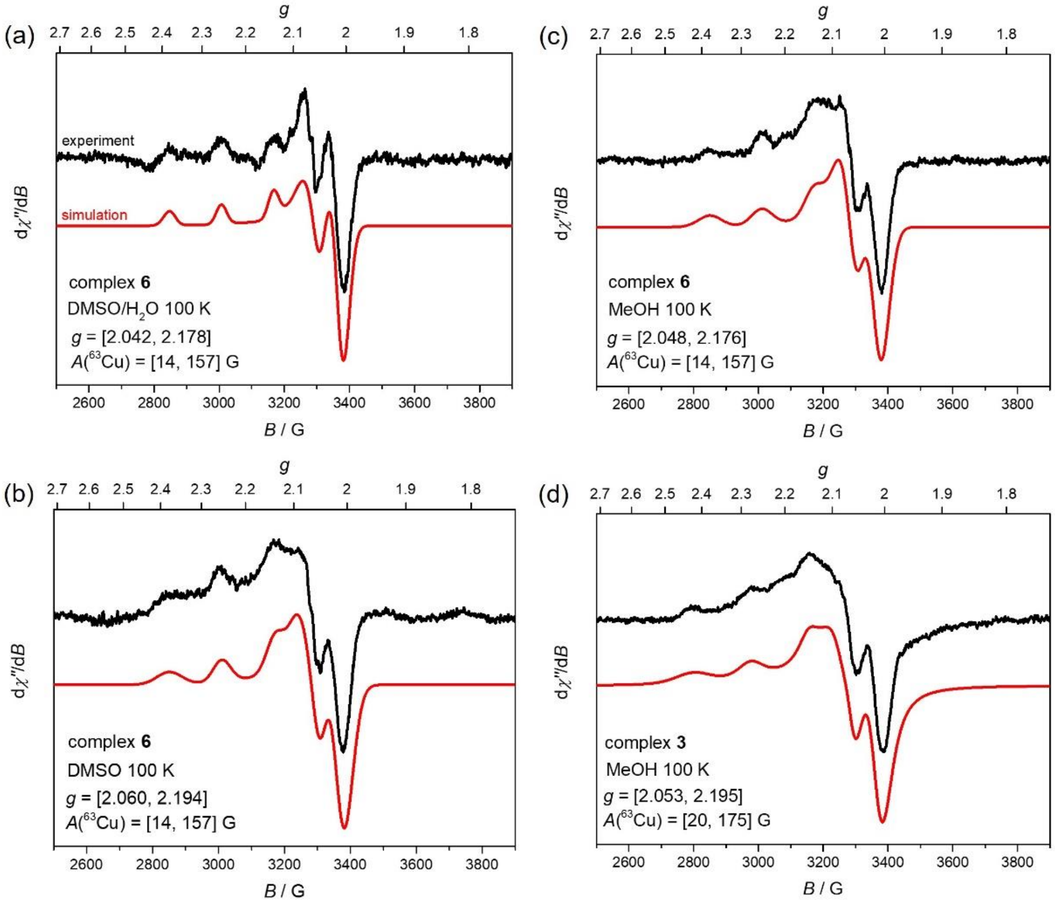


**Figure S11.** Experimental (black traces) and simulated (red traces) EPR spectra of frozen solutions of **6** in (a) 30% DMSO/$H_2O$ solvent mixture, (b) in DMSO and in (c) methanol (MeOH) as well as of frozen solutions of **3** in (d) MeOH measured at 100 K (the EPR simulation parameters are shown in the insets of the corresponding figures). Experimental settings: modulation amplitude – 5 G; microwave power – 2 mW.

## 5. Details of the investigation of proton dissociation processes of $H_2L^3$–$H_2L^6$ by pH potentiometric and spectroscopic ($^1$H NMR and UV–vis) methods

Proton dissociation processes were first determined by pH-potentiometric titrations of the proligands in the 30% (v/v) DMSO/$H_2O$ solvent mixture. Moderate water solubility of the hybrids precluded the use of neat water as solvent, and DMSO was added to decrease the polarity of the medium. Only one p$K_a$ value could be determined reliably from the recorded titration curves in the pH range of 5.16 – 5.64 (Table 1). However, two other deprotonation steps were also observed at pH<2.5 and pH>10. Therefore, the deprotonation steps were further studied by $^1$H NMR spectroscopy by monitoring the spectra of $\mathbf{H_2L^4}$ in 30% (v/v) DMSO-$d_6$/$H_2O$ in the pH range from 0.7 to 10.1 (Figure S11a).

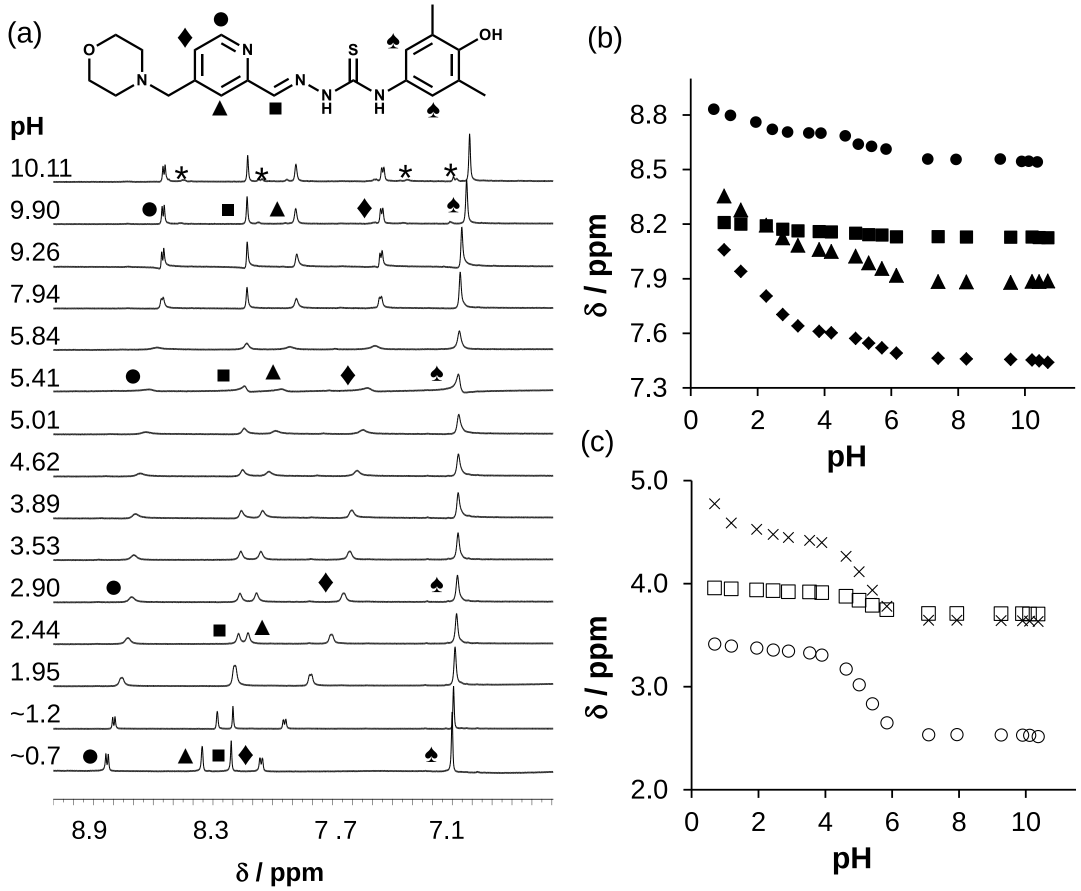


**Figure S12.** (a) $^1$H NMR spectra for $\mathbf{H_2L^4}$ in the low-field range at various pH values with symbols used for proton resonance assignment, the symbol * denotes the new peaks appearing upon oxidation; (b) pH-dependence of the chemical shifts of peaks belonging to the pyridine-ring protons (●,▲,♦) and the protons (■) of the C$H$=N moiety and (c) to the methylene-

morpholine protons: N-C$H_2$-C$_{morpholine}$ (×), C$H_2$-CH$_2$-N (□), CH$_2$-C$H_2$-N (○); ($c_L$ = 1 mM; $I$ = 0.1 M (KCl); $T$ = 25 °C; 30% (v/v) DMSO-$d_6$/$H_2O$).

Broad signals appearing in the spectra between pH 2.0 and 7.9 may indicate isomerization. It is worth mentioning that the peaks of the methyl and C$H$ protons of the dimethylphenol ring were markedly shifted only at pH > 9.5, implying the deprotonation of the phenolic-OH in this pH range, but these peaks can also be sensitive to the protonation state of the hydrazinic-NH moiety. Additional peaks also appeared at pH > 10, most likely due to oxidation processes involving the potentially redox active 2,6-dimethyl-4-aminophenol unit of the proligand. The methylene-morpholine protons were sensitive to deprotonation in the pH range 4 – 6 (Figure S11a,c) and a p$K_a$ = 5.2 ± 0.1 could be calculated on the basis of these changes. Therefore, this p$K_a$ belongs to the deprotonation of the morpholinium-$NH^+$ group. The peaks in the low-field region of the spectra (Figure S11a,b) (C$H$ aromatic protons of the pyridine ring and C$H$=N) were high-field shifted upon the first and second deprotonation step, but remain unchanged between pH ~7 and ~10. This suggests that the first deprotonation step can be attributed to the pyridinium-$NH^+$ moiety, while the deprotonation of the hydrazonic-NH was not observed up to pH 10. At pH > 10 the overlapping deprotonation of the phenolic-OH and hydrazonic-NH is possible, but in this pH range the oxygen-sensitivity of the proligand was strongly increased.

UV–vis electronic absorption spectra were also measured at different pH values, and representative spectra for $\mathbf{H_2L^4}$ are shown in Figure S12. For the determination of the lowest p$K_a$ individual samples were prepared in which KCl was partially or completely replaced by HCl, and pH values, varying in the range of *ca*. 1.0 – 2.0, were calculated from the HCl content of the solution. Characteristic spectral changes were observed in the pH range 1.0 – 3.4 (Figure S12a) accompanied by the appearance of the isosbestic point at 350 nm. The other proligands behaved similarly. Therefore, p$K_a$ value for the deprotonation of the pyridinium-$NH^+$ was determined for each proligand by the deconvolution of the spectra recorded at pH < 3.4 (Table 1), except for $\mathbf{H_2L^6}$, where this deprotonation step took place even at a more acidic pH. On the contrary, deprotonation of the morpholinium-$NH^+$ resulted in only minor changes in the UV–vis spectra, as expected for a non-chromophoric unit. On the other hand, the development of an overlapping less intensive band was seen between 370 and 450 nm, most probably due to the concomitant existence of *Z* and *E* isomers in the pH range between 2 and 8 (similarly to related TSCs in ref. 1). As the 2,6-dimethylphenolic group deprotonates in the basic pH range, the development of a strong band with $\lambda_{max}$ = 394 nm and a decrease of the absorption maximum

at 324 nm was seen (Figure S12b). Irreversible alteration of the spectra was observed at pH > 10.9, most likely due to the oxidation of the 4-aminophenolate unit to 4-aminophenoxyl radical ($1e^-$ oxidation) and/or to 4-benzoquinone imine ($2e^-$ oxidation with release of two protons in total) by the oxygen traces. These side reactions hindered the accurate determination of p$K_a$ of the proligands for deprotonation processes in the basic pH range. The spectral changes were even more obvious when oxygen was bubbling through the solutions of the samples in this pH range.

The reference aminophenol compound without morpholine moiety (2-formylpyridine 4-(4-hydroxy-3,5-dimethylphenyl)thiosemicarbazone) was also characterized, and two p$K_a$ values (3.01 and 10.55) could be determined by UV−vis spectrophotometry.[2] Based on these data, this compound is neutral at pH 7.4 and also found to be air-sensitive in the strongly basic pH range (pH > 11).

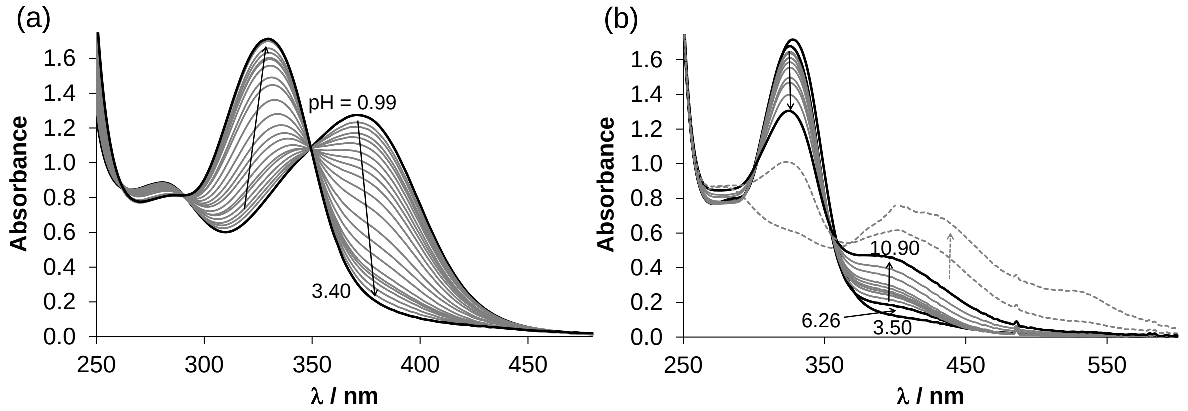


**Figure S13.** UV–vis absorption spectra recorded for **$H_2L^4$** at various pH values (a) in the pH range 0.99 – 3.40 and (b) 3.50 – 11.61. ($c_L$ = 73.7 μM; 30% (v/v) DMSO/$H_2O$; $I$ = 0.1 M (KCl); $T$ = 25 °C).

## 6. Details of solution speciation studies of 3–6

Representative UV–vis spectra and absorbance changes as a function of pH are shown for the copper(II) – **$H_2L^3$** system in Figure S13. Comparison of the spectrum of the **$H_2L^3$** and **3** at pH ~1 showed a low extent of dissociation of the complex under this condition, and this made the direct determination of the formation constants of the complexes difficult. The complex formation, enhanced upon increase of the pH, is supposed to be complete at pH ~ 3. The ligand binds to the metal ion via the ($N_{pyridine}$,$N_{aldimine}$,$S^-$) donor set in agreement with SC-XRD studies. The thiolato group likely resulted from deprotonation of the hydrazonic-NH and redistribution of electron density, as already reported for other α-*N*-pyridyl thiosemicarbazones.[3] Small but remarkable spectral changes were seen in the pH range 3–6. The $\lambda_{max}$ shifted from 419 to 413

nm, and isosbestic points at 297 and 334 nm also appeared, suggesting an equilibrium process between two species. p$K_a$ values of the complexes between 4.2 and 4.7 (Table 2 in the main text) were computed by the deconvolution of the absorbance spectra recorded in this pH range. Notably, there were no changes detected in the case of the Cu(II) – $\mathbf{H_2L^6}$ system, and a p$K_a$ could not be determined. An X-ray diffraction study of **6** revealed the involvement of the morpholine moiety in coordination to copper(II) (Figure 2d in the main text). In contrast, the p$K_a$ values for copper(II) complexes of $\mathbf{H_2L^3}$ – $\mathbf{H_2L^5}$ could be determined and assigned to the deprotonation of the non-coordinating morpholinium-$NH^+$ group in the complexes $[Cu(H_2L)]^{2+}$. The complex formed was $[Cu(HL)]^+$, in which, in addition, the pyrininium-$NH^+$ and the hydrazonic-NH were deprotonated, while the phenolic-OH remained intact. The suggested structures for the $[Cu(H_2L)]^{2+}$ and $[Cu(HL)]^+$ complexes of $\mathbf{H_2L^3}$ are shown in Chart S1. pH-potentiometric titrations were performed for the copper(II) complexes of $\mathbf{H_2L^4}$ and $\mathbf{H_2L^5}$, and the p$K_a$ values of $[Cu(H_2L)]^{2+}$ obtained by the two different methods were in acceptable agreement (Table 2).

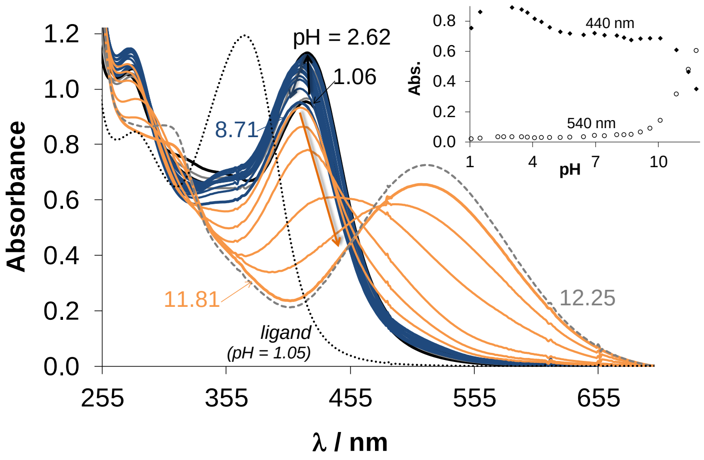


**Figure S14.** UV–vis absorption spectra recorded for the copper(II) – $\mathbf{H_2L^3}$ (1:1) system at various pH values (solid and dashed lines) together with the spectrum of the $\mathbf{H_2L^3}$ at pH 1.05 (dotted black line). The inserted figure shows the absorbance changes at 440 (♦) and 540 nm (○) as a function of pH ($c_L$ = $c_{Cu(II)}$ = 67 μM; 30% (v/v) DMSO/$H_2O$; $I$ = 0.1 M (KCl); $t$ = 25 °C).

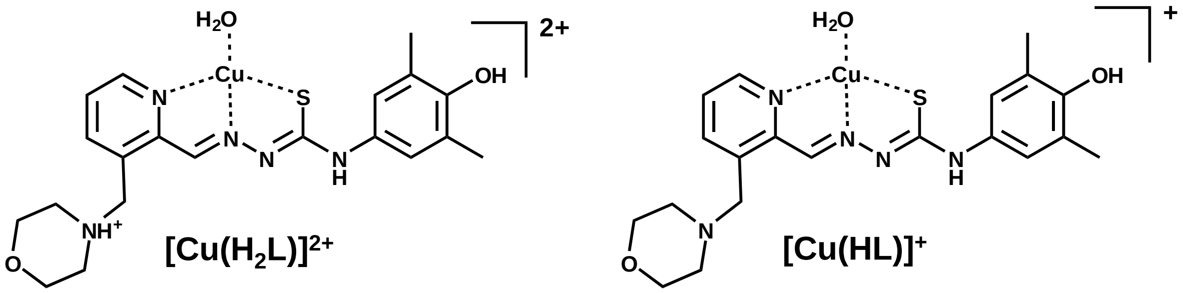


**Chart S1.** Suggested structures for $[Cu(H_2L)]^{2+}$ and $[Cu(HL)]^{+}$ complexes of $\mathbf{H_2L^3}$.

Upon increasing the pH, more obvious changes in the UV–vis spectra were observed. In particular, at pH > 8.7 (Figure S13), the $\lambda_{max}$ was shifted from 414 to 516 nm for $\mathbf{H_2L^3}$, most likely due to the deprotonation of the phenol group. This process resulted in the formation of the neutral complex [CuL]. The formation of phenolate increases the sensitivity of the complex to oxidation. In the case of $\mathbf{H_2L^4}$–$\mathbf{H_2L^6}$, the absorbance values decreased in this pH range at $\lambda$ > 340 nm, while an increase of absorbance was seen at ~310 nm, most likely (at least in part) due to precipitate formation, but the oxidation of the phenolate function also occurred. Both of these processes precluded the evaluation of spectral data collected in the basic pH range. However, the spectra remained unchanged between pH 5.5 and 8.7 (copper(II)–$\mathbf{H_2L^3}$ system) or ~7 (in the cases of the other three proligands) due to the predominant formation of the $[Cu(HL)]^{+}$ complexes in this pH range.

## 7. Electrochemistry and spectroelectrochemistry

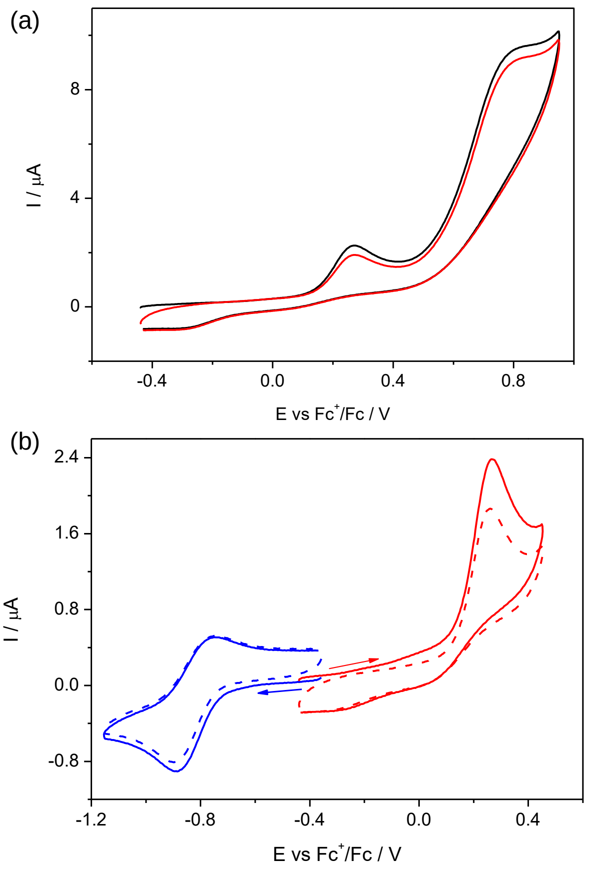


**Figure S15**. (a) CVs of **3** in DMSO/*n*-$Bu_4NPF_6$ at Pt-working electrode at scan rate of 100 mV $s^{-1}$ (black trace – 1st scan, red trace – 2nd scan) in the anodic part and (b) CVs of **3** in the anodic part going to the first electron transfer (red traces) and in the cathodic part (blue traces) in DMSO/*n*-$Bu_4NPF_6$ at a Pt-working electrode at a scan rate of 100 mV $s^{-1}$ (solid lines – 1st scan, dashed lines – 2nd scan).

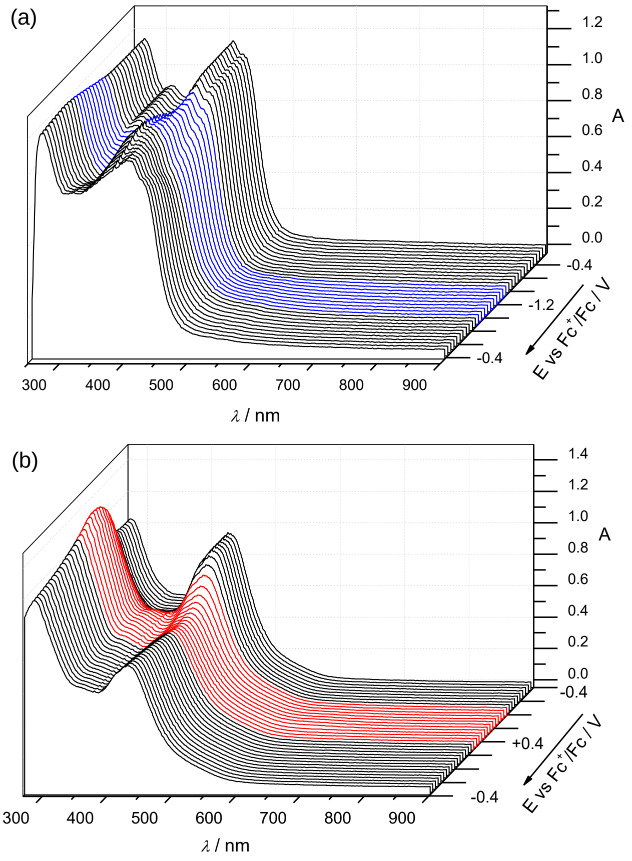


**Figure S16**. UV−vis spectra measured (a) upon cyclic voltammetry of **6** at the first reduction peak and (b) upon anodic oxidation of **3** at the first oxidation peak by using a honeycomb Pt working electrode (scan rate 10 mV $s^{-1}$).

## 8. ROS generation

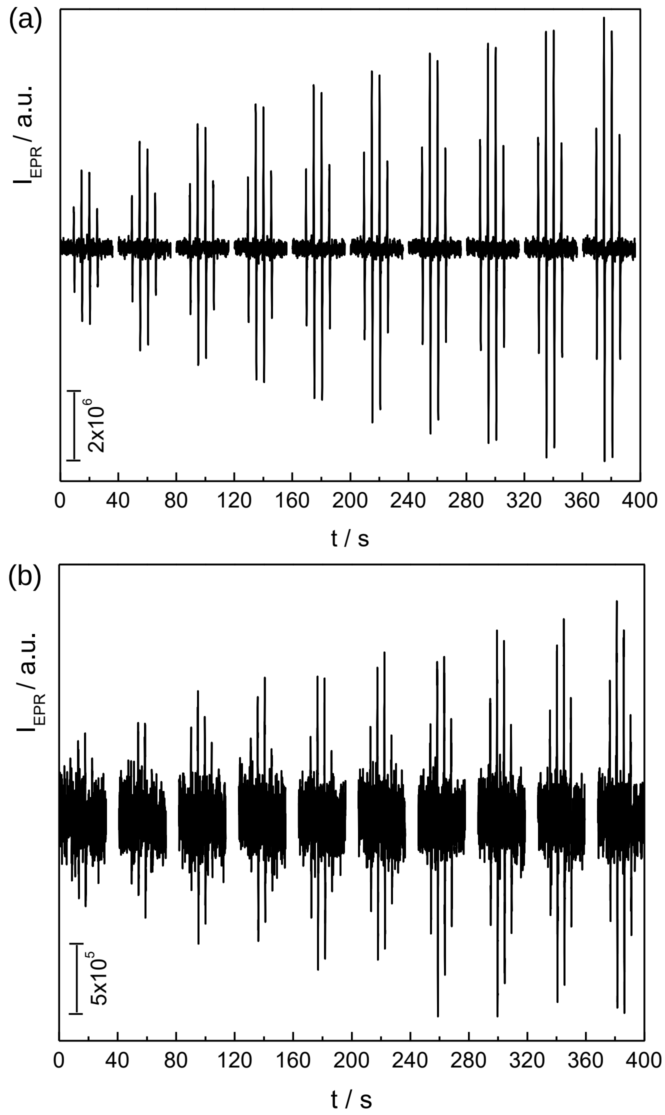


**Figure S17.** Time course of the EPR spectra monitored 2 min after the addition of $H_2O_2$ into the water solution of **6** under air in the presence of the spin trapping agent DMPO. Initial concentrations: (a) $c_0$(**6**) = 32 μM and (b) $c_0$(**6**) = 8 μM ($c_0$(DMPO) = 0.02 M, $c_0$($H_2O_2$) = 0.01 M.

## 9. Cell cycle analysis

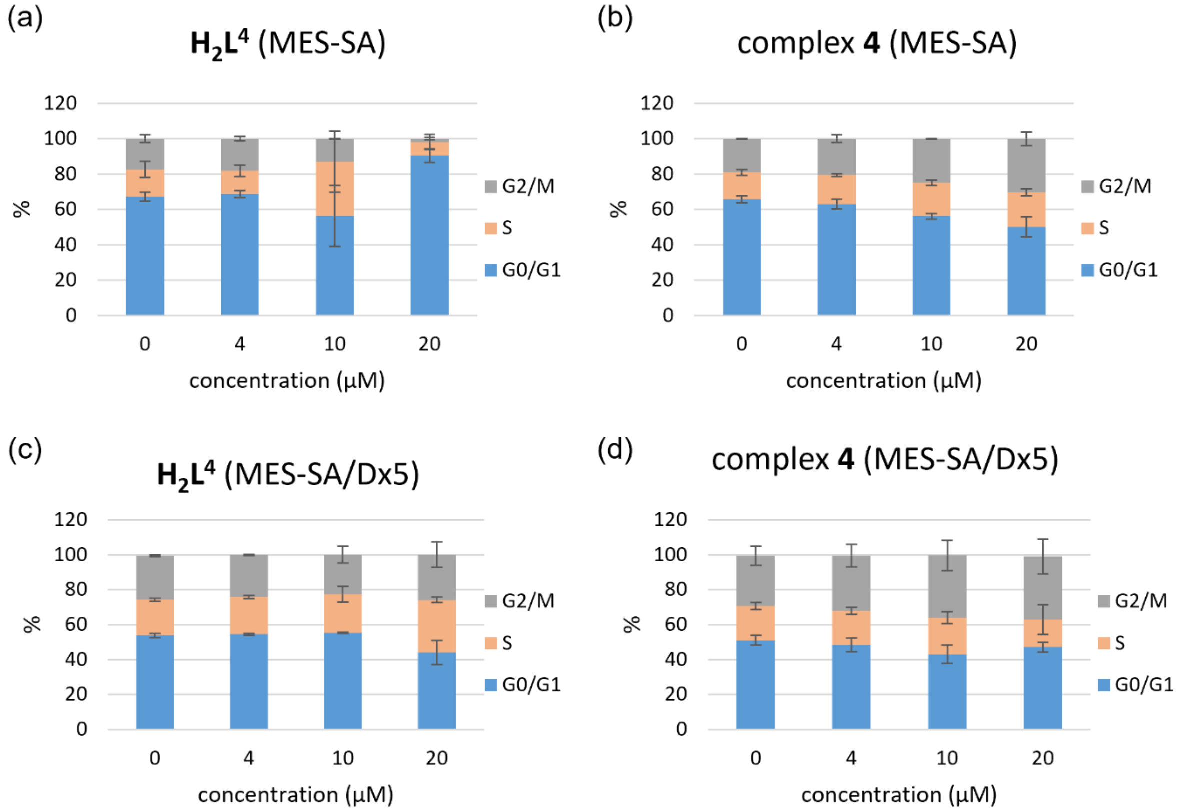


**Figure S18**. Cell cycle arrest for (a) proligand **$H_2L^4$** and (b) complex **4** in MES-SA cells, as well as for (c) proligand **$H_2L^4$** and (d) complex **4** in the MES-SA/Dx5 cancer cell line.

**Table S4**. Cell cycle arrest for proligand **$H_2L^4$** and complex **4** in the MES-SA and MES-SA/Dx5 cancer cell lines.

| | **$H_2L^4$** (MES-SA) | | | **$H_2L^4$** (MES-SA/Dx5) | | | complex **4** (MES-SA) | | | complex **4** (MES-SA/Dx5) | | |
|---|---|---|---|---|---|---|---|---|---|---|---|---|
| **c [μM]** | G0/G1 | S | G2/M | G0/G1 | S | G2/M | G0/G1 | S | G2/M | G0/G1 | S | G2/M |
| 0 μM | 67% | 15% | 18% | 54% | 20% | 25% | 66% | 15% | 19% | 51% | 20% | 29% |
| 2 μM | 69% | 13% | 18% | 54% | 21% | 24% | 63% | 16% | 21% | 48% | 20% | 32% |
| 4 μM | 56% | 31% | 13% | 55% | 22% | 23% | 56% | 19% | 25% | 43% | 21% | 36% |
| 8 μM | 90% | 8% | 2% | 44% | 30% | 26% | 50% | 20% | 30% | 47% | 16% | 36% |

**Table S5**. Cell cycle arrest for proligand **$H_2L^6$** and complex **6** in the MES-SA and MES-SA/Dx5 cancer cell lines.

| | **$H_2L^6$** (MES-SA) | | | **$H_2L^6$** (MES-SA/Dx5) | | | complex **6** (MES-SA) | | | complex **6** (MES-SA/Dx5) | | |
|---|---|---|---|---|---|---|---|---|---|---|---|---|
| **c [μM]** | G0/G1 | S | G2/M | G0/G1 | S | G2/M | G0/G1 | S | G2/M | G0/G1 | S | G2/M |
| 0 μM | 66% | 16% | 17% | 43% | 21% | 36% | 67% | 16% | 17% | 53% | 18% | 30% |
| 2 μM | 71% | 15% | 14% | 45% | 21% | 34% | 74% | 9% | 17% | 53% | 12% | 35% |
| 4 μM | 72% | 14% | 14% | 46% | 20% | 33% | 35% | 11% | 55% | 39% | 12% | 48% |
| 8 μM | 77% | 12% | 10% | 47% | 20% | 33% | 24% | 8% | 68% | 21% | 11% | 68% |

## 10. Molecular docking

**Table S6.** The results of the scoring functions for the ligands **$H_2L^3$–$H_2L^6$** and their Cu(II) complexes **3–6** against the R2 RNR crystal structure.

| Sr. No. | Compound | GS | ChemPLP | CS | ASP | H-Bonding(HB)/ Lipophilic Contact(LC) |
|---|---|---|---|---|---|---|
| 1 | **$H_2L^6$** | 54.6 | 55.7 | 26.4 | 31.5 | Asp272(HB), Glu335(HB), Phe237(LC), Phe241(LC), Leu334(LC), Val328(LC) |
| 2 | **$H_2L^5$** | 52.0 | 60.9 | 28.6 | 28.4 | Phe241(LC), Leu332(LC), Cys271(LC) Val328(LC), Leu334(LC), |
| **3** | **$H_2L^4$** | 54.4 | 57.6 | 26.9 | 32.5 | Tyr324(HB), Phe237(LC), Phe241(LC), Leu334(LC), Val328(LC) |
| 4 | **$H_2L^3$** | 53.6 | 60.8 | 27.4 | 33.1 | Gly268(HB), Asp272(HB), Arg265(LC), Phe237(LC), Phe241(LC), Val328(LC), Leu332(LC), Arg331(LC), Cys271(LC) |
| 5 | **6** | 50.4 | | | | Arg331(LC), Val328(LC), Phe241(LC), Phe245(LC) Phe237(LC), |
| 6 | **5** | 50.2 | | | | Arg331(LC), Phe237(LC), Leu334(LC), Phe241(LC), Val328(LC) |
| 7 | **4** | 50.2 | | | | Tyr324(HB), Val328(LC), Leu334(LC), Phe237(LC), Phe241(LC), Cys271(LC), |
| 8 | **3** | 51.8 | | | | Arg265(LC), Leu269(LC), Val328(LC), Arg331(LC), Le,u332(LC) |

**Table S7**. Molecular descriptors for the proligands **$H_2L^3$–$H_2L^6$** and and their Cu(II) complexes **3–6**.

| Sr. No | Molecule | MW | Donor HB | Accpt. HB | Log P | PSA | Rotatable Bonds |
|---|---|---|---|---|---|---|---|
| **1** | **$H_2L^6$** | 399.5 | 3 | 9.4 | 2.3 | 87.5 | 7 |
| **2** | **$H_2L^5$** | 398.5 | 2 | 9.4 | 2.2 | 89.2 | 7 |
| **3** | **$H_2L^4$** | 399.5 | 3 | 9.4 | 2.3 | 87.6 | 7 |
| **4** | **$H_2L^3$** | 399.5 | 3 | 9.4 | 2.3 | 86.6 | 7 |
| **5** | **6** | 497.5 | 3 | 5 | 4.0 | 77.9 | 1 |
| **6** | **5** | 497.5 | 0 | 6 | 3.0 | 78.4 | 2 |
| **7** | **4** | 497.5 | 2 | 6 | 3.6 | 81.2 | 3 |
| **8** | **3** | 497.5 | 2 | 6 | 3.6 | 81.2 | 3 |
| **Reference** | **Triapine** | 195.2 | 5 | 3 | 0.5 | 89.3 | 2 |

**Table S8.** The binding affinities as predicted by the scoring functions for the colchicine site of tubulin. DAMA-colchicine is the co-crystallized ligand. Root-mean-square deviation – RMSD from the co-crystallized ligand (heavy atoms) in Å.

| Complexes | GS | Proligands | GS | CS | ChemPLP | ASP |
|---|---|---|---|---|---|---|
| **3** | 59.2 | **$H_2L^3$** | 66.9 | 24.9 | 66.8 | 27.7 |
| **4** | 57.3 | **$H_2L^4$** | 63.0 | 26.1 | 64.3 | 27.7 |
| **5** | 65.4 | **$H_2L^5$** | 64.2 | 28.3 | 63.1 | 26.4 |
| **6** | 58.4 | **$H_2L^6$** | 64.4 | 28.2 | 65.1 | 28.2 |
| | | DAMA-colchicine | 61.9 | 21.6 | 60.1 | 17.4 |
| | | RMSD: | 7.5155 | 2.8032 | 1.0908 | 7.1644 |

## References


(1) Dömötör, O.; May, N. V.; Pelivan, K.; Kiss, T.; Keppler, B. K.; Kowol, C. R.; Enyedy, É. A. A comparative study of α-*N*-pyridyl thiosemicarbazones: spectroscopic properties, solution stability and copper(II) complexation. *Inorg. Chim. Acta* **2018**, *472*, 264–275.

(2) Besleaga, I.; Stepanenko, I.; Petrasheuskaya, T. V.; Darvasiova, D.; Breza, M.; Hammerstad, M.; Marć, M. A.; Prado-Roller, A.; Spengler, G.; Popović-Bijelić, A.; Enyedy, E. A.; Rapta, P.; Shutalev, A. D.; Arion, V. B. Triapine analogues and their copper(II) complexes: synthesis, characterization, solution speciation, redox activity, cytotoxicity, and mR2 RNR inhibition. *Inorg. Chem.* **2021**, *60*, 11297–11319.

(3) Enyedy, É. A.; Nagy, N. V.; Zsigó, É.; Kowol, C. R.; Arion, V. B.; Keppler, B. K.; Kiss, T. Comparative solution equilibrium study of the interactions of copper(II), iron(II) and zinc(II) with triapine (3-aminopyridine-2-carbaldehyde thiosemicarbazone) and related ligands. *Eur. J. Inorg. Chem.* **2010**, 1717–1728.
